# Design, synthesis, and antibacterial assessment of a new series of ciprofloxacin-based compounds as possible dual DNA gyrase/topoisomerase IV inhibitors

**DOI:** 10.1038/s41598-026-50106-z

**Published:** 2026-04-30

**Authors:** Lamya H. Al-Wahaibi, Hayat Ali Alzahrani, Stefan Bräse, Bahaa G. M. Youssif, Mohamed Hisham

**Affiliations:** 1 Department of Chemistry, College of Sciences, Princess Nourah bint Abdulrahman University, Riyadh, 11671 Saudi Arabia; 2 Medical Laboratory Technology Department, Applied Medical Science College, Northern Border University, Arar, Saudi Arabia; 3 Institute of Biological and Chemical Systems, IBCS-FMS, Karlsruhe Institute of Technology, 76131 Karlsruhe, Germany; 4 Pharmaceutical Organic Chemistry Department, Faculty of Pharmacy, Assiut University, Assiut, 71526 Egypt; 5 Pharmaceutical Chemistry Department, Faculty of Pharmacy, Deraya University, Minia, Egypt

**Keywords:** Fluoroquinolone, Ciprofloxacin, Bacterial resistance, DNA gyrase, Topo IV, Anti-biofilm, Biochemistry, Computational biology and bioinformatics, Drug discovery, Microbiology

## Abstract

Simultaneous inhibition of DNA gyrase and topoisomerase IV (Topo IV) is a primary pharmacological strategy to enhance antibacterial efficacy and markedly reduce the emergence of antibiotic resistance. In this regard, a new set of twelve ciprofloxacin-based derivatives was rationally developed, synthesized, and structurally verified. The DNA gyrase and Topo IV inhibitory actions of the developed Compounds **6a-l** were investigated. Compound **6 g** showed the most promising results, with IC_50_ values of 1.75 ± 0.05 and 03.47 ± 0.14 µM against DNA gyrase and Topo IV, respectively, compared to ciprofloxacin at 02.13 ± 0.06 and 25.22 ± 1.27 µM, respectively. Compound **6 g** demonstrated the highest antibacterial activity, with MIC values of 0.025, 0.025, and 0.125 µg/mL against *E. coli*, *P. aeruginosa*, and *S. aureus*, respectively. It exhibits comparable efficacy to ciprofloxacin against *E. coli*, a gram-negative bacterium, although it possesses only half the potency against *P. aeruginosa* and the gram-positive *S. aureus*. Compound **6 g** exhibits a significant antibiofilm action; at the MIC level, the biofilm inhibition percentage was 96%. Docking analyses revealed that Compound **6 g** displays enhanced binding affinity for *E. coli* DNA gyrase B and Topo IV compared to ciprofloxacin. Molecular dynamics simulations validated the exceptional stability of the **6 g**–DNA gyrase B complex. In silico ADMET studies demonstrated satisfactory lipophilicity and metabolic characteristics. These findings collectively underscore **6 g** as a viable antibacterial candidate.

**Supplementary Information:**

The online version contains supplementary material available at 10.1038/s41598-026-50106-z.

## Introduction

**Fig. 1:**
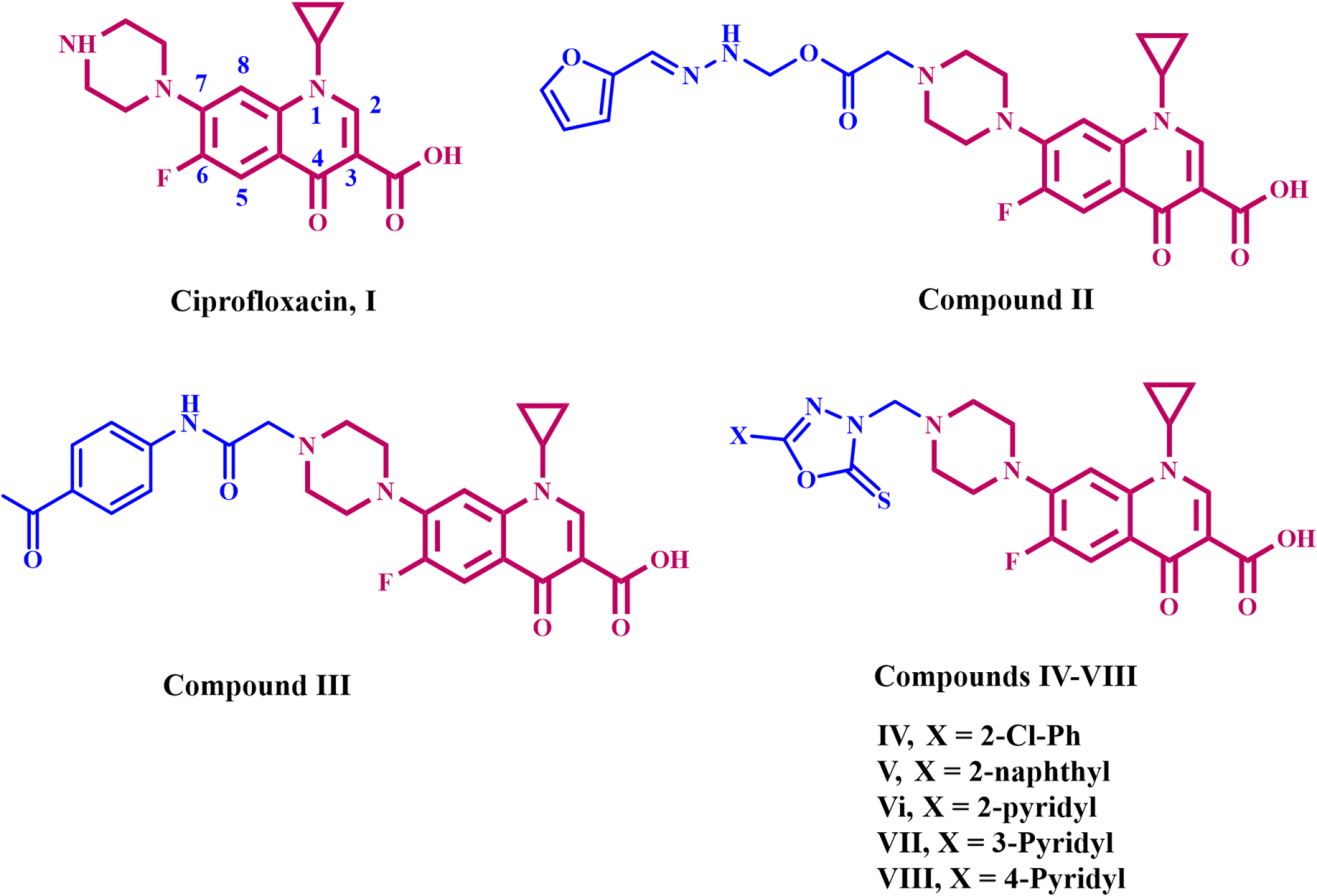
Structure of ciprofloxacin (**I**) and some reported ciprofloxacin-based derivatives **II-VIII**.

Ciprofloxacin (**I**, Fig. 1) is a quinolone antibiotic and one of the top five most commonly produced generic antibiotics globally^1,2^. It is one of the most frequently employed antibiotics for the treatment of a variety of bacterial infections affecting the gastrointestinal, urinary, respiratory, and abdominal systems^3–7^. Fluoroquinolones have good tolerability, a robust safety profile, and helpful pharmacokinetic properties, in addition to their broad range of antibacterial activity^8,9^. They constitute a fundamental category of pharmacophores, essential to drug discovery and development. The discovery of fluoroquinolone-based drugs has attracted significant interest due to their extensive medicinal properties^10,11^. Ciprofloxacin might decrease bacterial antibiotic resistance when administered at reduced doses alongside other antibiotics^12,13^. The antibacterial mechanism of ciprofloxacin involves the inhibition of type II topoisomerase enzymes, particularly DNA gyrase, which plays a crucial role in DNA replication, recombination, and repair^14,15^. Moreover, it can bind to topoisomerase IV. Topoisomerase protein–DNA complexes become weakened, resulting in DNA damage, triggering cell death pathways, and disrupting normal DNA replication^16–18^.

The discovery and development of new drugs with potential clinical applications and novel mechanisms of action are difficult, expensive, and time-consuming^19,20^. Many researchers choose a more cost-effective approach to improving treatment efficacy by modifying existing drugs rather than inventing new therapies and conducting clinical trials^21,22^. The primary technique in drug development involves using the structure-activity relationships of lead compounds, such as existing medications, to formulate analogues with improved therapeutic properties^23,24^.

Ciprofloxacin derivatives have recently attracted significant attention due to their exceptional pharmacological properties^25^. Ciprofloxacin’s molecular structure features a central core with a quinolone nucleus, a fluorine atom at position C-6, and a piperazine ring at position C-7^3,26^. Researchers have employed various synthetic methodologies to modify the chemical structure of ciprofloxacin, aiming at generating derivatives with improved antibacterial efficacy^27^. This may entail modifications to the molecule’s fundamental structure or the incorporation of functional groups to enhance its efficacy against specific microbial infections. Nonetheless, most research has focused on modifying two key active sites in the ciprofloxacin structure: the carboxylic acid group at C-3 and the piperazine group at C-7^28,29^. The two sites are frequently modified because changes in these regions can significantly affect the drug’s pharmacological properties. The incorporation of various groups at the C-7 position of the quinolone nucleus affects the compound’s potency, bioavailability, physicochemical properties, and its affinity for DNA gyrase and/or Topoisomerase IV^3,30^.

Yang et al.^31^ efficiently developed twelve new fluoroquinolone derivatives by conjugating *N*-acyl arylhydrazone to ciprofloxacin at the C-7 position and tested their efficiency in preventing the growth of particular Gram-negative and Gram-positive bacteria. The antibacterial study revealed that introducing acyl hydrazone derivatives at the C-7 position of ciprofloxacin yielded compounds with higher activity against *S. aureus* than ciprofloxacin itself. Compound **II** (Fig. 1) was the most efficient against the pathogens tested, with MIC values of 1 µg/mL for *S. aureus* and *E. coli* and 16 µg/mL for *P. aeruginosa*. Furthermore, it demonstrated anti-MRSA activity with a MIC of 32 µg/mL. Time-killing studies revealed that Compound **II** is a promising option, maintaining ciprofloxacin’s rapid bactericidal activity against *E. coli* (within 2 h) and *S. aureus* (within 4 h). Furthermore, cytotoxicity and hemolysis studies revealed that the compounds were relatively safe in vitro. Molecular docking investigations revealed that Compound **II** has a higher affinity for topoisomerase IV than ciprofloxacin, with a binding energy of − 9.9 kcal/mol.

Elgedamy et al.^32^ developed and synthesised a novel group of ciprofloxacin hybrids that function as antibacterial and antifungal agents. Compound **III** (Fig. 1) was tested for antibacterial activity against many bacterial strains, including *S. aureus*, *MRSA*, and *E. coli*. Antifungal effectiveness was also tested against *Candida albicans*. Compared with the original antibiotic, ciprofloxacin, the amide derivative **III** showed strong antibacterial activity against all strains tested. The addition of a bulky functional group to the C-7 position of ciprofloxacin has a substantial impact on its antibacterial activity, spectrum, and safety profile. The inclusion of an amide group significantly improved the antibacterial efficacy.

In 2021^33^, we synthesised a novel class of ciprofloxacin hybrid molecules comprising several heterocyclic derivatives, which were evaluated in vitro against Gram-negative and Gram-positive bacteria, including *E. coli*, *P. aeruginosa*, *S. aureus*, and *B. subtilis*. Compounds **IV-VIII (**Fig. 1**)**, oxadiazole derivatives, demonstrated antibacterial efficacy between 88% and 120% relative to ciprofloxacin against both Gram-positive and Gram-negative microorganisms. Compound **VIII** had superior effectiveness (120%) against *S. aureus*, compared to ciprofloxacin. Oxadiazoles **VI** and **VII** have demonstrated efficacy similar to that of ciprofloxacin against *S. aureus* and *E. coli*. Prior research reveals that hybridizing oxadiazole and thiadiazole moieties with a ciprofloxacin compound improves its efficiency^34,35^.

**Fig. 2:**
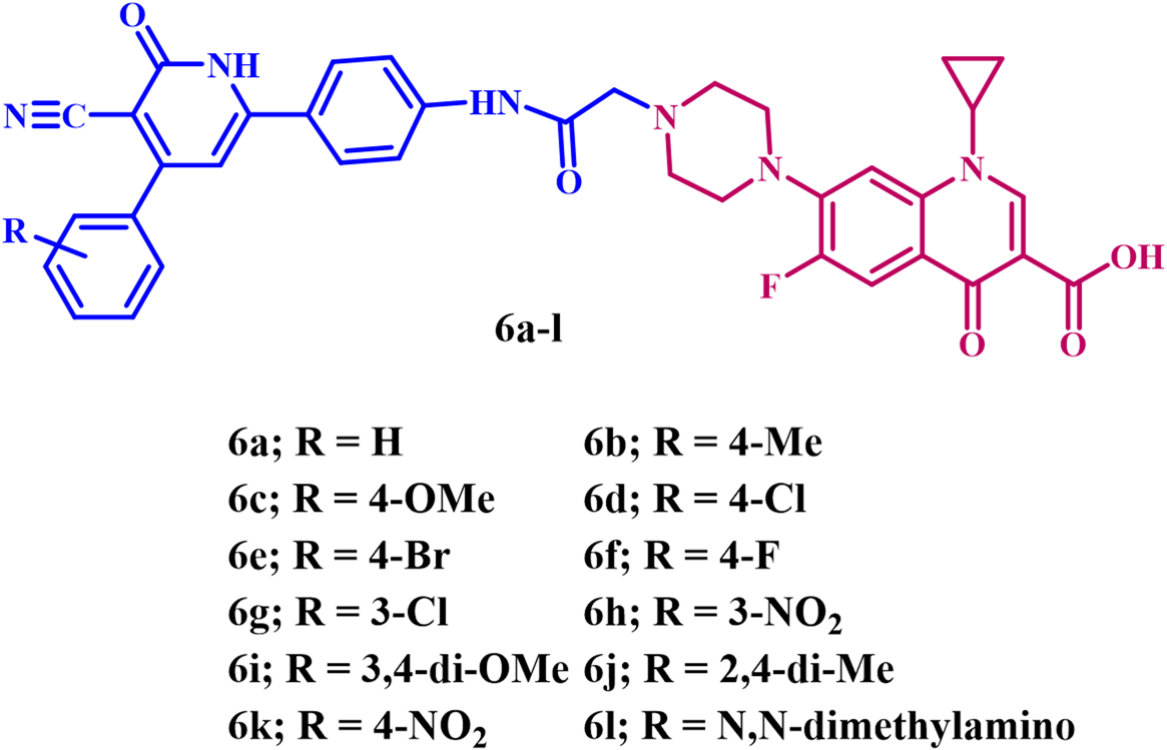
Structures of new Compounds **6a-l**.

Based on the prior data, and in continuation of our effort to develop a dual DNA gyrase and Topo IV inhibitor^33,36–40^, we report herein the design, synthesis, and antibacterial evaluation of a novel series of ciprofloxacin-based derivatives **6a-l** (Fig. 2**)**. The novel compounds are fluoroquinolone derivatives formed by conjugating aryl pyridine moiety to ciprofloxacin at the C-7 position, and their efficacy as potential dual inhibitors of DNA gyrase and Topo IV, as well as their ability to inhibit the growth of specific Gram-negative and Gram-positive bacteria, has been evaluated.

## Result and discussion

### Chemistry

**Scheme 1:**
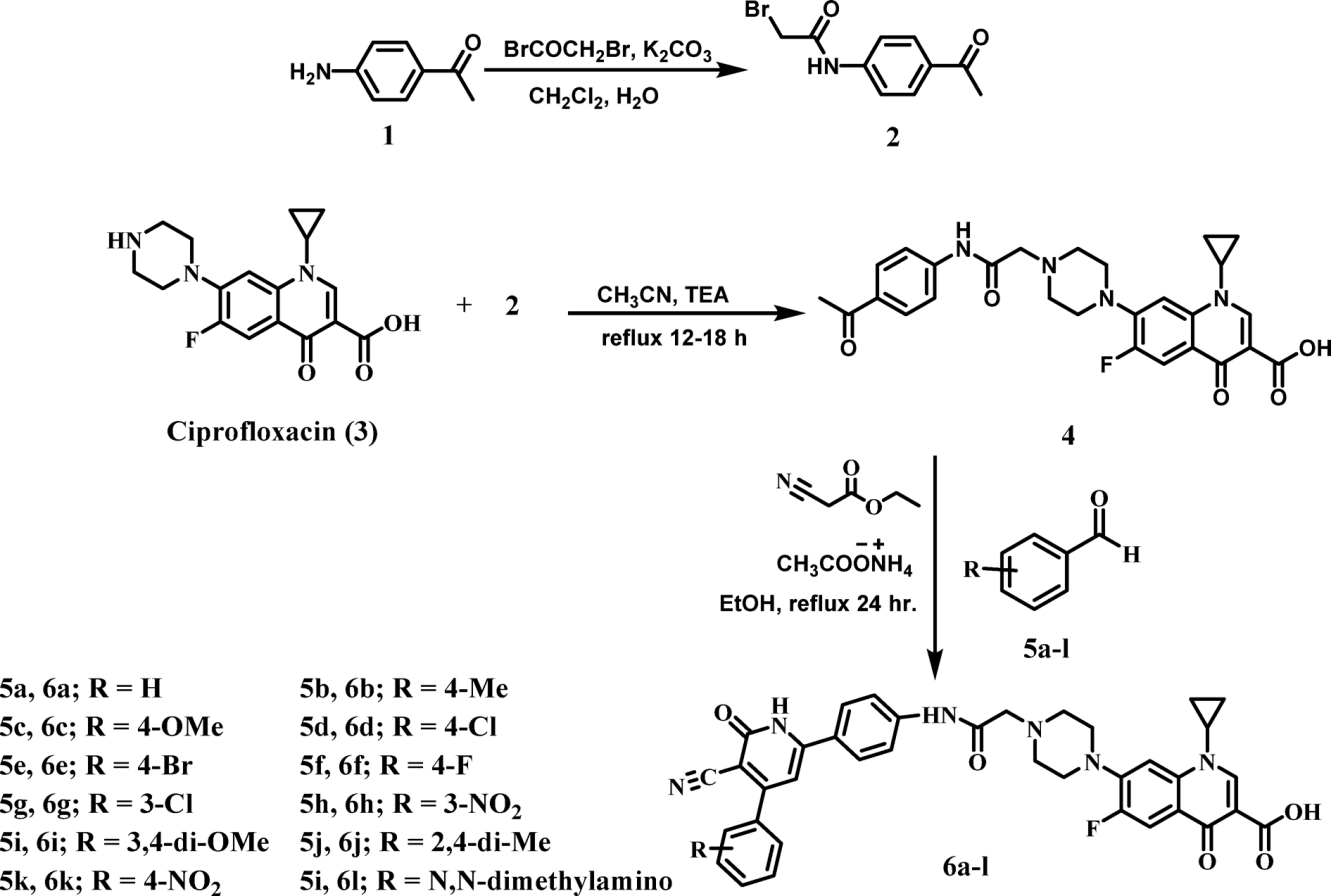
Synthesis of target Compounds **6a-l**.

The synthesis of the target Compounds (**6a**-**l**) proceeded via the route outlined in Scheme 1. The initial step, following a literature procedure^41^, involved the reaction of *p*-aminoacetophenone (**1**) with bromoacetyl bromide. This reaction was conducted under biphasic conditions using water and methylene chloride with potassium carbonate as a base, yielding the key intermediate *N*-(4-acetylphenyl)−2-bromoacetamide (**2**) in high yield. In the subsequent step, alkylation of ciprofloxacin (**3**) with intermediate **2** successfully provided the corresponding ketone derivative (**4**) in good yield.

The final ciprofloxacin/3-cyano-2-pyridone conjugates **6a–l** were then synthesized from this intermediate using a one-pot, four-component reaction, adapting a reported methodology^42^. This was achieved by reacting Compound **4** with equimolar amounts of ethyl cyanoacetate and the appropriate benzaldehydes **5a–l** in the presence of an excess of ammonium acetate. The reaction was performed in absolute ethanol under vigorous stirring at 120–130 °C for 1.5 h, affording the target hybrids **6a–l**.

The ¹H NMR spectral data for the synthesized hybrids **6a–l** confirmed the presence of characteristic signals from both the ciprofloxacin and 3-cyano-2-pyridone components. For example, the spectrum of Compound **6d** featured a singlet at δ 3.30 ppm, assigned to the methylene protons of the COC***H***₂–N group. A distinct singlet at δ 6.81 ppm was attributed to the C_5_–H proton of the 3-cyano-2-pyridone ring. Key amidic and heterocyclic NH signals were also observed, including a singlet at δ 10.14 ppm for the amidic NH and a singlet at δ 12.64 ppm for the NH proton of the 3-cyano-2-pyridone ring. Furthermore, the characteristic acidic proton of the ciprofloxacin moiety appeared as a strongly downfield-shifted singlet at δ 15.22 ppm.

The ¹³C NMR spectrum of Compound **6d** further corroborated the hybrid structure, displaying characteristic resonances consistent with its molecular framework. The methylene carbon linking the ciprofloxacin piperazinyl moiety was observed at δ 36.72 ppm (CO**CH₂**–N). Signals at δ 49.79 and 52.76 ppm were assigned to the carbons of the piperazinyl ring within the ciprofloxacin core. The nitrile carbon appeared at δ 117.00 ppm. Furthermore, the carbonyl carbons were clearly distinguished, with the pyrid-2-one carbonyl resonating at δ 169.28 ppm and the amidic carbonyl at δ 176.79 ppm. These distinguishing spectral characteristics were consistently identified across all synthesized derivatives (**6a-l**), indicating that the target structures were successfully developed.

### Biology

#### DNA gyrase B inhibitory assay

Using the *E. coli* DNA gyrase assay^31,32^, the newly synthesized compounds were examined for potential DNA gyrase inhibition. Ciprofloxacin was used as the reference compound (Table 1**)**.

Compounds **6a-l** showed substantial DNA gyrase inhibitory activity, with IC_50_ values ranging from 1.75 to 160.70 µM, compared with the reference Ciprofloxacin, which exhibited an IC_50_ of 2.13 µM. Compounds **6a**, **6b**, **6d-6f**, **6i**, and **6 L** exhibited the most pronounced DNA gyrase inhibitory action, with IC_50_ values ranging from 1.75 to 14 µM. Compounds **6d** and **6 g** displayed greater activity than Ciprofloxacin against DNA gyrase B, with IC_50_ values of 1.75 and 1.87 µM, respectively, compared to 2.13 µM for Ciprofloxacin.

Compound **6 g** (*R* = 3-Cl) surpassed all other examined compounds. The compound had an IC_50_ of 1.75 ± 0.05 µM, rendering it 1.2-fold more potent than ciprofloxacin (IC_50_ = 2.13 ± 0.06 µM). Compound **6d** (*R* = 4-Cl) exhibited the second-highest DNA gyrase inhibitory activity, with an IC_50_ value of 1.87 ± 0.06 µM, demonstrating equivalent potency to Compound **6 g**. Prior data indicated that chlorine atoms at positions 3 and 4 on the phenyl group of the pyridine moiety are appropriate for inhibitory activity^43^.

The type of substituents on the phenyl group at the fourth position of the cyanopyridine moiety greatly influences the inhibitory activity of the new compounds. Compounds **6e** (*R* = 4-Br) and **6f** (*R* = 4-F), which contain 4-bromo and 4-fluoro atoms at the four positions of the phenyl group of the 3-cyanopyridine moiety, had lower DNA gyrase inhibitory efficiency than the 3-chloro derivative, **6 g** (*R* = 3-Cl), and the 4-chloro derivative, Compound **6d** (*R* = 4-Cl). Compound **6e** had an IC_50_ of 11.40 µM, making it 6.5-fold less potent than **6 g** (IC_50_ = 1.75 µM), and Compound **6f** had an IC_50_ of 2.70 µM, making it 1.6-fold less effective than Compound **6 g**. The data show that both fluorine and chlorine atoms at the 4-position on the phenyl ring of the 3-cyanopyridine moiety are more effective at inhibiting DNA gyrase than the bromine atom, with activity increasing in the pattern 3-Cl > 4-Cl > 4-F > 4-Br.

Compounds **6b** (*R* = 4-Me), **6i** (*R* = 3,4-di-OMe), and **6 L** (*R* = 4-*N*,* N*-dimethylamino) significantly inhibit DNA gyrase, with IC_50_ values of 6.15, 5.72, and 3.89 µM, respectively. Compound **6 L** (IC_50_ = 3.89 µM) had the fourth greatest activity and was twice as potent as **6 g**. These data revealed that the *N*,* N*-dimethylamino group on the phenyl group of the 3-cyanopyridine moiety is tolerated for its inhibitory effect on DNA gyrase, following the chlorine and fluorine atoms in the fourth position. Finally, Compounds **6c** (*R* = 4-OMe), **6k** (*R* = 4-NO_2_), and **6j** (*R* = 2,4-di-Me) demonstrated the lowest potency, with IC_50_ values of 57.73, 160.70, and 29.67 µM, respectively, representing at least 17-fold lower potency than Compound **6d**. The results indicated that neither methoxy, nitro, nor methyl groups are compatible with inhibitory activity against DNA gyrase.

#### Topoisomerase IV inhibitory assay

Using the topoisomerase IV assay^37,38^, the newly synthesized compounds were examined for possible Topo IV inhibition. Ciprofloxacin was used as the reference compound **(**Table 1**)**. Compounds **6a-l** exhibited prominent topoisomerase IV (Topo IV) inhibitory activity, with IC_50_ values ranging from 3.47 to 83.44 µM, compared with the reference Ciprofloxacin, which had an IC_50_ of 25.22 µM. Compounds **6a**, **6c**, **6d**, **6f**, **6 g**, **6i**, and **6 L** exhibited the highest potency as Topo IV inhibitors, with IC_50_ values between 3.47 and 22.06 µM. All seven compounds exhibit greater efficacy than the reference, ciprofloxacin, with potency increases ranging from 1.2-fold to 7.2-fold.

Compound **6 g** (*R* = 3-Cl), the most effective DNA gyrase inhibitor, also exhibited the highest potency as a Topo IV inhibitor, with an IC_50_ value of 3.47 µM, compared to ciprofloxacin’s IC_50_ value of 25.22 µM. Compound **6 g** exhibited approximately a sevenfold enhancement in potency compared to the reference ciprofloxacin, underscoring the significance of the chlorine atom at the 3-position of the phenyl ring within the 3-cyanopyridine moiety for both Topo IV and DNA gyrase inhibitory activities. Compound **6 L** (*R* = 4-*N*,* N*-dimethylamino) exhibited the second-highest activity, with an IC_50_ value of 4.50 µM, demonstrating a potency 1.3-fold inferior to that of **6 g** as a Topo IV inhibitor; however, it is still 5.6-fold more potent than the reference ciprofloxacin. Moreover, compound **6f** (*R* = 4-F) exhibited substantial Topo IV inhibitory activity with an IC_50_ value of 5.56 µM, demonstrating a potency 4.5 times greater than that of ciprofloxacin. Compound **6f** had the third highest activity against both DNA gyrase and Topo IV, demonstrating an IC_50_ value of 2.70 µM against DNA gyrase, comparable to the reference ciprofloxacin (IC_50_ = 2.13 µM against DNA gyrase). The data indicate that 3-Cl, 4-F, and 4-*N*,* N*-dimethylamino substituents are acceptable for the inhibitory activity against both DNA gyrase and Topo IV.

Compound **6i** (*R* = 3,4-di-OMe) exhibited reduced activity compared to ciprofloxacin as a DNA gyrase inhibitor, with an IC_50_ value of 5.72 µM, indicating it is 2.7-fold less effective than ciprofloxacin. However, it demonstrated more efficacy than ciprofloxacin as a Topo IV inhibitor. Compound **6i** had an IC_50_ of 8.17 µM as a Topo IV inhibitor, demonstrating potency 3 times that of ciprofloxacin in this assay. Compound **6d** (*R* = 4-Cl) is the second-most potent DNA gyrase inhibitor, with an IC_50_ of 1.87 µM, exceeding that of ciprofloxacin; yet it is ranked fifth as a Topo IV inhibitor. Compound **6d** had an IC_50_ value of 11.92 µM, demonstrating double the potency of ciprofloxacin against Topo IV. The studies indicated that **6d** exhibits greater potency than ciprofloxacin against both DNA gyrase and Topo IV, underscoring the importance of the 4-Cl atom for inhibitory efficacy, and demonstrating that chlorine atoms in both the 3- and 4-positions are well tolerated for this action. Finally, Compounds **6a** (*R* = H) and **6c** (*R* = 4-OMe) demonstrated superior potency as Topo IV inhibitors compared to ciprofloxacin, with IC_50_ values of 22.06 and 15.70 µM, respectively, indicating 1.2- and 1.6-fold greater potency than ciprofloxacin (IC_50_ = 25.22 µM). Conversely, both compounds exhibited reduced inhibitory activity against DNA gyrase, with IC_50_ values of 14.09 and 57.73 µM, rendering them at least 7-fold less potent than ciprofloxacin.

**Table 1:** DNA gyrase and Topoisomerase IV inhibitory results of Compounds **6a-l**.


Comp.	*R*	IC_50_ (µM)	IC_50_ (µM)
		E. Coli DNA gyrase	E. Coli Topo IV
**6a**	H	14.09 ± 0.41	22.06 ± 0.89
**6b**	4-Me	6.15 ± 0.18	35.34 ± 1.42
**6c**	4-OMe	57.73 ± 1.70	15.70 ± 0.63
**6d**	4-Cl	1.87 ± 0.06	11.92 ± 0.48
**6e**	4-Br	11.40 ± 0.34	27.31 ± 1.10
**6f**	4-F	2.70 ± 0.08	5.56 ± 0.22
**6g**	3-Cl	1.75 ± 0.05	3.47 ± 0.14
**6h**	3-NO_2_	9.70 ± 0.29	40.99 ± 1.65
**6i**	3,4-di-OMe	5.72 ± 0.17	8.17 ± 0.33
**6j**	2,4-di-Me	29.67 ± 0.87	83.44 ± 3.36
**6k**	4-NO_2_	160.70 ± 4.73	31.23 ± 1.26
**6l**	N, N-dimethylamino	3.89 ± 0.11	4.50 ± 0.18
**Ciprofloxacin**	NA	2.13 ± 0.06	25.22 ± 1.27

NA: Not Applicable.

#### In vitro antibacterial evaluation

The antibacterial efficacy of Compounds **6d**, **6f**, **6 g**, **6i**, and **6 L**, the most potent dual inhibitors within the series, was evaluated against two Gram-positive bacteria, *S. aureus* (RCMB010010) and *B. subtilis* (RCMB015 (1) NRRL B-543), as well as two Gram-negative bacteria, *E. coli* (RCMB 010052 ATCC 25955) and *P. aeruginosa* (RCMB 004 (1) ATCC 13315). The disc-diffusion method^19^ was employed to determine the inhibition zones (IZ, mm/mL) and the minimal inhibitory concentration (MIC, µg/mL). Ciprofloxacin served as positive control. Table 2 displays the results.

Generally, the tested Compounds **6d**, **6f**, **6 g**, **6i**, and **6 L** exhibited inhibition zones that were either superior to or comparable with those of the reference drug Ciprofloxacin against Gram-negative (G^−^) organisms, while demonstrating inferior zones against Gram-positive (G^+^) bacteria. The inhibition zone diameters for *E. coli* (27–33 mm) and *P. aeruginosa* (22–25 mm) are commendable. The zones for *S. aureus* (13–21 mm) and *B. subtilis* (all < 12 mm) are significantly smaller. The data reveal that the assessed derivatives demonstrate significantly superior efficacy against Gram-negative bacteria in comparison to Gram-positive pathogens.

Compound **6 g** (*R* = 3-Cl), the most effective dual inhibitor of DNA gyrase and Topo IV, had enhanced antibacterial activity, particularly against Gram-negative pathogens, with an inhibition zone diameter of 33 mm against *E. coli*, comparable to or surpassing ciprofloxacin’s 30 mm. Additionally, Compound **6 g** exhibited antibacterial efficacy similar to ciprofloxacin against *P. aeruginosa*, with an inhibition zone diameter of 24 mm, compared to 25 mm for ciprofloxacin. Compound **6 g** had negligible antibacterial action against *B. subtilis* among Gram-positive bacteria. It exhibited significant activity against *S. aureus*, with an inhibitory zone diameter of 21 mm, in contrast to 24 mm for ciprofloxacin. The facts obviously demonstrate that **6 g** is the most promising contender. It demonstrates the greatest efficacy against *E. coli* (33 mm) and is the only agent that retains significant action against *S. aureus* (21 mm). It is the closest approach to a “real” broad-spectrum lead.

Compounds **6d**, **6f**, and **6 L** demonstrated substantial antibacterial activity against the Gram- negative bacteria *E. coli* and *P. aeruginosa*, with inhibition zone widths matched those of ciprofloxacin, while exhibited reduced activity against the Gram-positive strains. These data indicate that Compounds **6d**, **6f**,** 6 g**, and **6 L** are possible dual inhibitors of DNA gyrase and Topo IV, exhibiting significant antibacterial activity, especially against Gram-negative strains.

**Table 2:** Diameter of inhibition zones (mm/mg) for Compounds **6d**, **6f**, **6g**, **6i**, **6l**, and Ciprofloxacin.

Compound	Inhibition Zone (IZ) Diameter (mm/mg)			
	Bacterial species			
	(G^+^)		(G^−^)	
	***B. subtilis***	***S. aureus***	***E. Coli***	***P. aeruginosa***
**6d**	< 12	15	30	25
**6f**	< 12	17	31	22
**6g**	< 12	21	33	24
**6i**	< 12	13	27	23
**6l**	< 12	14	29	24
**Ciprofloxacin**	26	24	30	25

NA: no activity (6 mm), weak activity (6–13 mm), moderate activity (13–18 mm), strong activity (> 18 mm), DMSO as solvent (6 mm), and *Bacillus subtilis* (*B. subtilis*), *Staphylococcus aureus* (*S. aureus*), *Escherichia coli* (*E. coli*), *Pseudomonas aeruginosa* (*P. aeruginosa*).

#### Minimum inhibitory concentration (MIC) assay

The antibacterial efficacy of the most effective Compounds, **6d**, **6f**, **6 g**, and **6 L**, was evaluated using the Broth microdilution method^44,45^. Table 3 presents the minimum inhibitory concentrations (MICs) of these compounds against the examined bacteria, with ciprofloxacin as the reference drug.

The results of this in vitro experiment correspond with the outcomes of the antimicrobial susceptibility test. Compound **6 g** (*R* = 3-Cl) had the greatest efficacy among the compounds investigated, with MIC values of 0.025, 0.025, and 0.125 µg/mL against *P. aeruginosa*, *E. coli*, and *S. aureus*, respectively. It exhibited comparable efficacy to ciprofloxacin against the G^−^ *E. coli*, while displaying approximately half the potency of ciprofloxacin against both *P. aeruginosa* and the gram-positive *S. aureus*. Compound **6d** (*R* = 4-Cl) had the second greatest level of activity. The MIC values were comparable to those of Compound **6 g** against the gram-negative bacteria *E. coli* and *P. aeruginosa*. Nonetheless, it exhibited a potency four times inferior to that of ciprofloxacin against gram-positive *S. aureus*. Finally, Compounds **6f** and **6 L** demonstrated significant efficacy against the studied species, especially *E. coli*, with MICs of 0.050 µg/mL, compared to ciprofloxacin, which had an MIC of 0.030 µg/mL.

**Table 3:** MICs values of Compounds **6d**, **6f**, **6g**, **6l** and ciprofloxacin.

MIC in µg/ml			
Compound	Bacterial species		
	(G^−^)		(G^+^)
	***P. aeruginosa***	***E. Coli***	***S. aureus***
**6d**	0.025	0.025	0.250
**6f**	0.050	0.050	0.125
**6g**	0.025	0.025	0.125
**6l**	0.125	0.050	0.250
**Ciprofloxacin**	0.010	0.030	0.060

#### Cell viability assay

This assay evaluates the safety of Compounds **6d** and **6 g**, the most effective derivatives in all in vitro antibacterial trials, by analyzing their impact on normal cell lines. The MCF-10 A cell line, a normal human mammary gland epithelial cell line [obtained from ATCC (American Type Cell Culture)], was used to determine the compounds’ cytotoxicity. The MTT assay was used to evaluate cell viability in MCF-10 A cells after a 4-day incubation with 50 µM of each compound tested^46^. The results indicated that at 50 µM, cell viability surpassed 92%, demonstrating that neither **6d** nor **6 g** exhibited cytotoxicity.

#### Anti-biofilm assay

Bacterial biofilms provide health hazards in healthcare facilities, the food sector, and potable water systems^47,48^. In the present study, we used the Microtiter plate assay^49,50^ for biofilm quantification to examine the antibiofilm activity of Compound **6 g**. Compound **6 g** was selected for this evaluation as it is the most effective and well-balanced dual inhibitor of DNA gyrase and Topoisomerase IV in the developed series. Due to its exceptional enzymatic profile and competitive antibacterial efficacy, **6 g** was selected as the primary candidate to demonstrate the proof-of-concept for the antibiofilm potential of this innovative 3-cyanopyridone-ciprofloxacin scaffold. Three distinct concentrations were used in the assay: the first was equal to the MIC of **6 g** against *E. coli*, the second was equal to half the MIC, and the third was equal to 1/4 the MIC. Table 4 displayed the results as a percentage of biofilm inhibition. According to the data, **6 g** has a considerable antibiofilm effect; at the MIC level, the biofilm inhibition percentage was 96. At ½ and ¼ MIC levels, Compound **6 g** reduced biofilms by 87 and 63%, respectively.

**Table 4:** Biofilm inhibition% of Compound **6 g**.

6 g	Biofilm Inhibition %	SD (±)
1/4 of MIC	63	0.50
1/2 MIC	87	0.60
MIC	96	0.40

### Docking study into *E. Coli* DNA gyrase B and *E. Coli* topoisomerase IV

This study performed a comprehensive computational docking investigation to clarify the binding interactions between Compounds **6d**, **6 g**, and the reference medication Ciprofloxacin with *E. coli* DNA gyrase B, as well as Compounds **6 g** and **6 L** with *E. coli* topoisomerase IV. The methodology utilized the Discovery Studio software^51^. We used the crystallographic structure of the *E. coli* DNA gyrase B ligand complex (PDB ID: 4DUH)^52^ to ensure the accuracy and relevance of our study. The energy of the examined molecular systems was minimized using the OPLS-AA (Optimized Potentials for Liquid Simulations - All Atom) force field^53^.

**Fig. 3:**
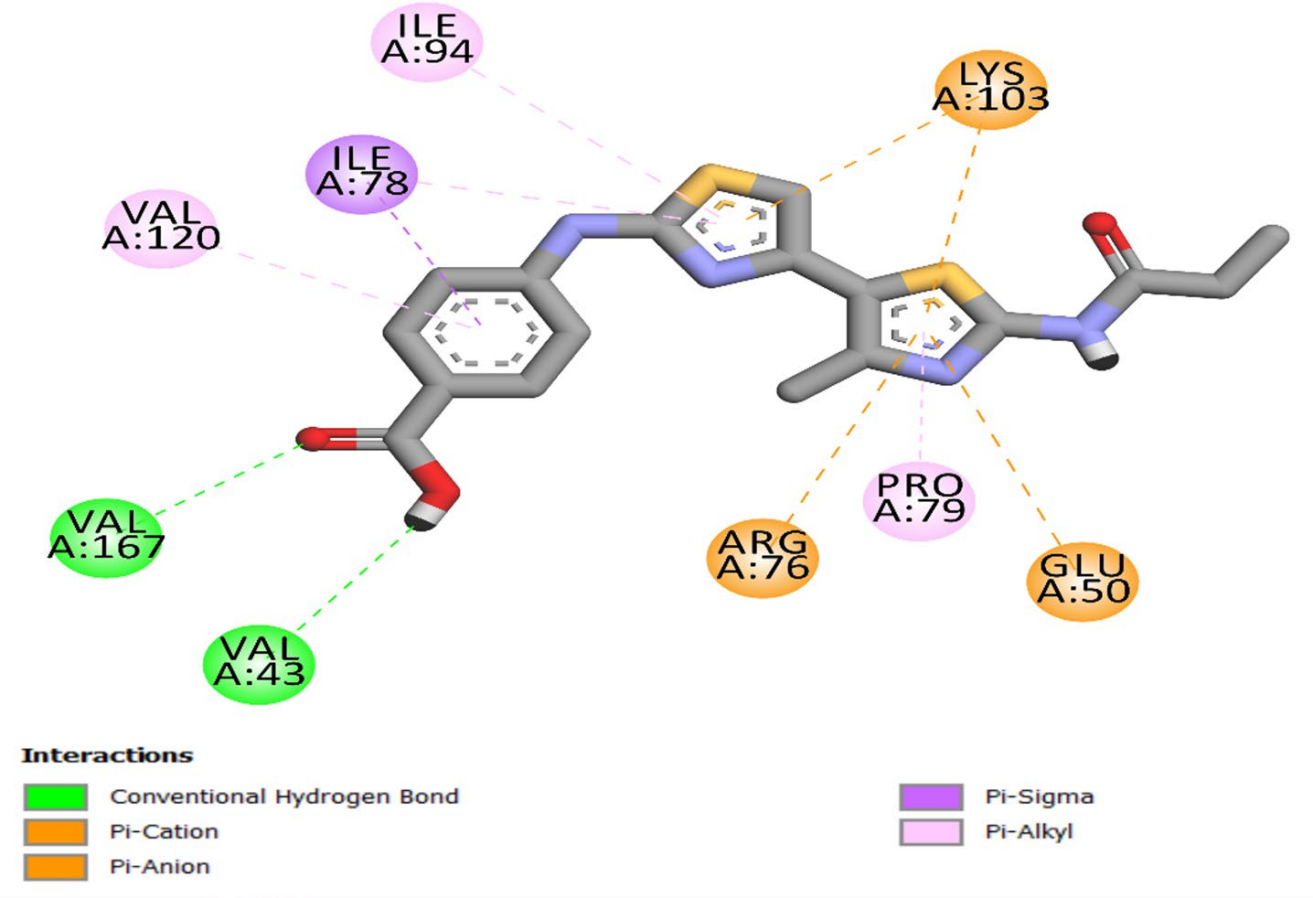
Two-dimensional model of co-crystallized ligand within the binding site of *E. coli* DNA gyrase B protein.

To verify the precision of the docking technique, the co-crystallized ligand was re-docked into the active site of the E. coli DNA gyrase B protein. The re-docking process yielded a S score of −8.10 kcal/mol and an RMSD value of 1.18, confirming the accuracy of the docking approach as depicted in Fig. 3.

**Fig. 4:**
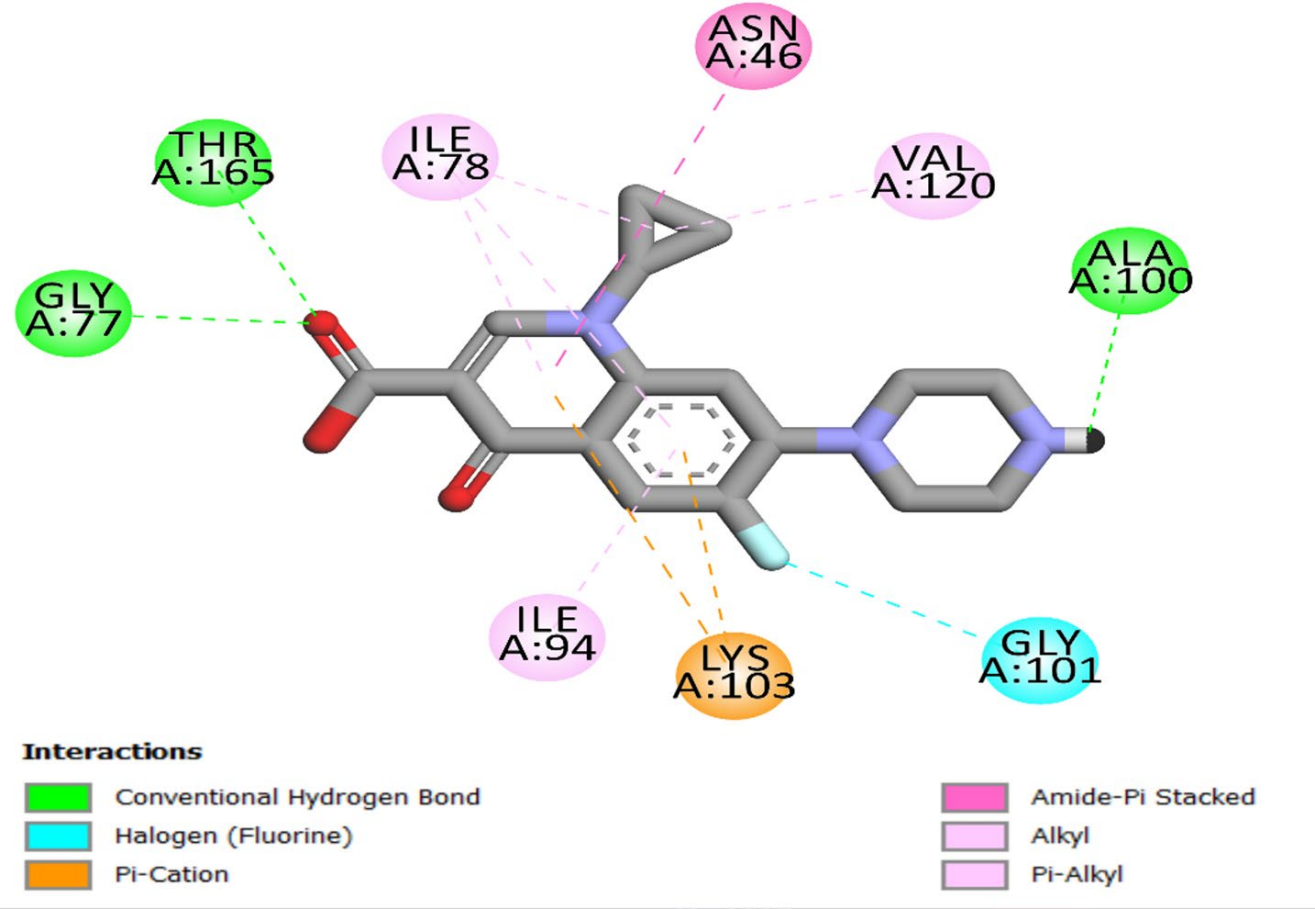
Two-dimensional docking representation models of Ciprofloxacin within the binding region of *E. coli* DNA gyrase B protein.

Ciprofloxacin was re-docked into the *E. coli* DNA gyrase B protein’s active site to confirm the accuracy of the docking procedure. The docking precision was confirmed by the re-docking approach, which yielded an S score of −9.0 kcal/mol. Along with other hydrogen bond interactions involving residues Ala100 and the piperazine ring, significant hydrogen bonds were found between the ligand’s carboxylic acid group and Thr165 and Gly77. The significance of molecular interactions in the binding process is highlighted by the fact that these interactions are necessary for stabilizing the ligand in the active site. As seen in Fig. 4, ciprofloxacin formed a pi-cation connection with Lys103, a halogen (fluorine) bond with Gly101, and many hydrophobic interactions with Asn46, Ile87, Ile94, and Val120 residues.

**Fig. 5:**
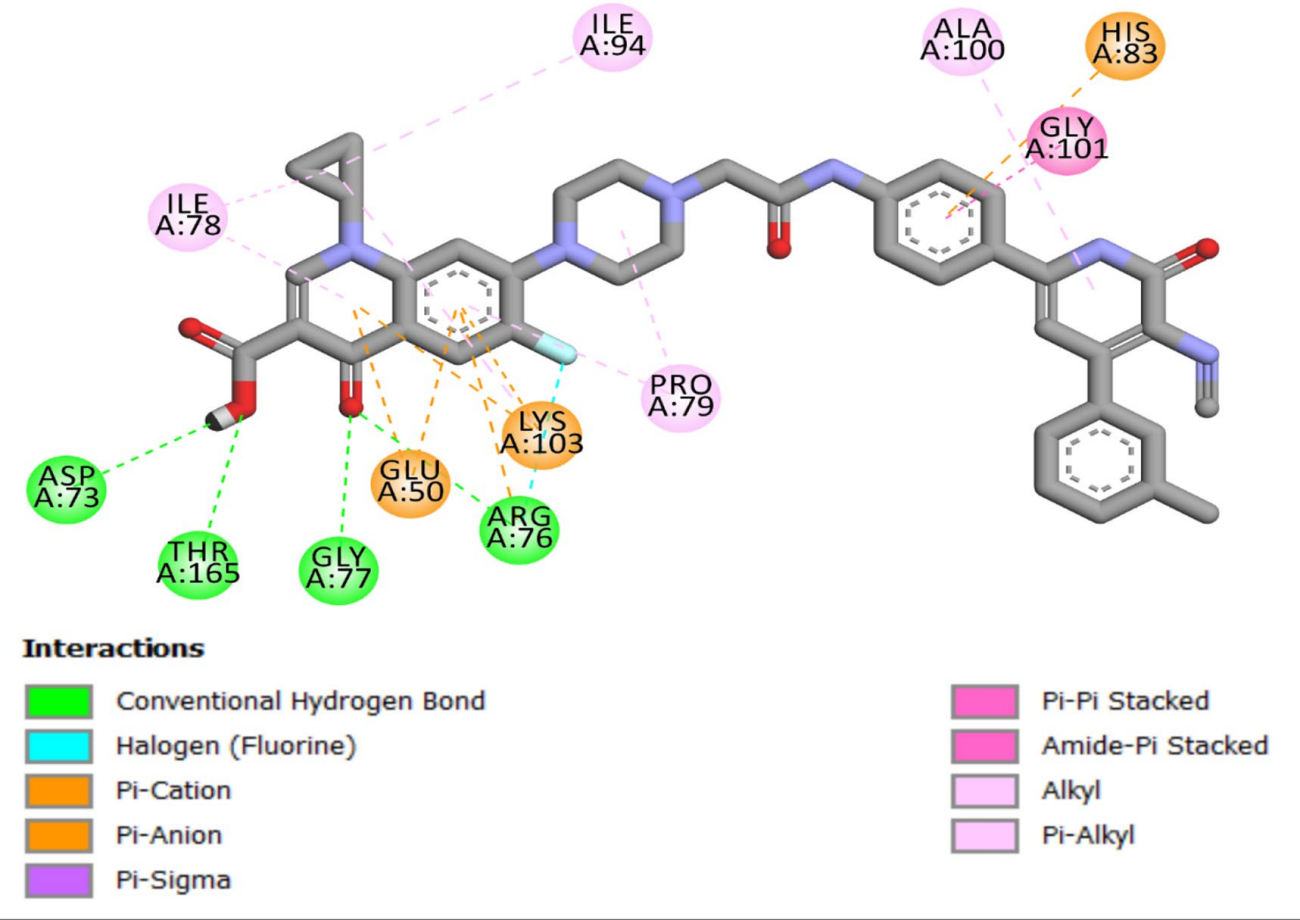
Two-dimensional docking representation models of **6 g** within the binding site of *E. coli* DNA gyrase B protein.

Compound **6 g**, demonstrating the highest in vitro activity against *E. coli* DNA gyrase B, achieved a docking score of −10.50 kcal/mol, indicating a direct correlation between docking scores and biological activity, suggesting that elevated docking scores are associated with enhanced affinity and, potentially, greater biological efficacy. In the examination of interactions between **6 g** and the *E. coli* DNA gyrase B protein, **6 g** has demonstrated significant binding. A notable interaction was observed in which the carboxylic group of 6 g acted as a hydrogen-bond acceptor. This interaction entailed the establishment of two hydrogen bonds with the essential amino acid residues Thr165 and Asp73 in DNA gyrase, signifying a vital enhancement of the compound’s binding affinity and specificity for the enzyme. Further detailing the binding dynamics of Compound **6 g**, the study highlighted the role of its carbonyl group. This group engaged in two hydrogen bonds with the amino acid residue Arg76 and Gly77, adding another interaction layer critical for stabilizing the active site, Fig. 5.

Furthermore, Compound **6 g** formed a halogen bond with Arg76 involving fluorine atom. The aromatic rings of **6 g** participated in pi-cation and pi-anion interactions with the residues Glu50, His83, and Lys103. The intricate interactions, including hydrogen bonding and pi-stacking, are crucial for the binding of **6 g** to the *E. coli* DNA gyrase B protein, as depicted in Fig. 5. This detailed interaction profile highlights the complex relationship between **6 g** and the target protein, enhancing its efficiency.

**Fig. 6:**
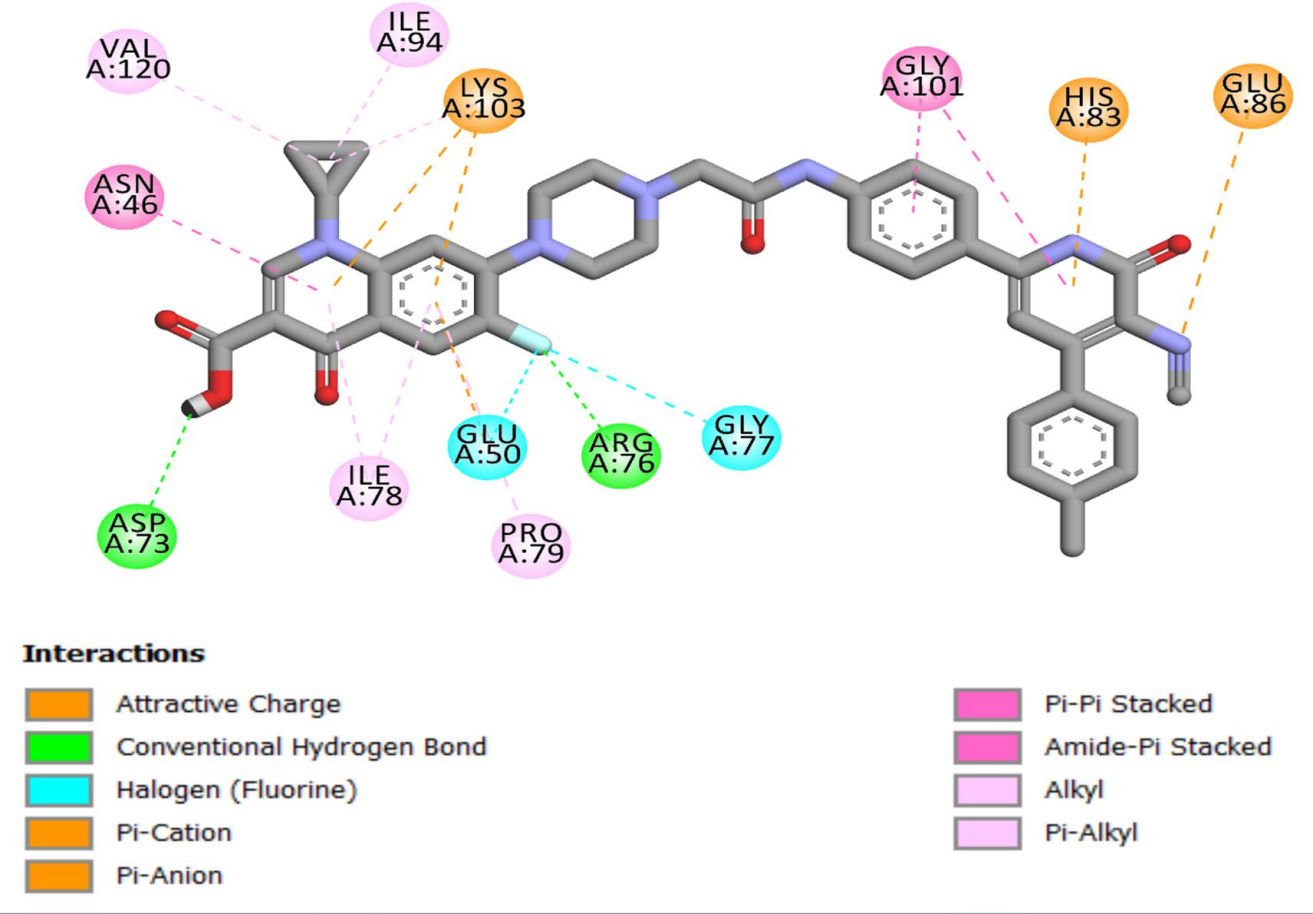
Two-dimensional docking representation models of **6d** within the binding site of *E. coli* DNA gyrase B protein.

Similarly, Compound **6d** exhibited significant interactions with *E. coli* DNA gyrase, specifically concerning Asp73 and Arg76, as illustrated in Fig. 6. The extent of significant hydrogen bonding interactions in **6d** was less than that in **6 g**, leading to a comparatively weaker affinity (S score of −10.10 kcal/mol) of **6d** for E. coli DNA gyrase. However, both **6 g** and **6d** demonstrated superior binding affinity compared to the reference ciprofloxacin, resulting in superior in vitro activity.

**Fig. 7:**
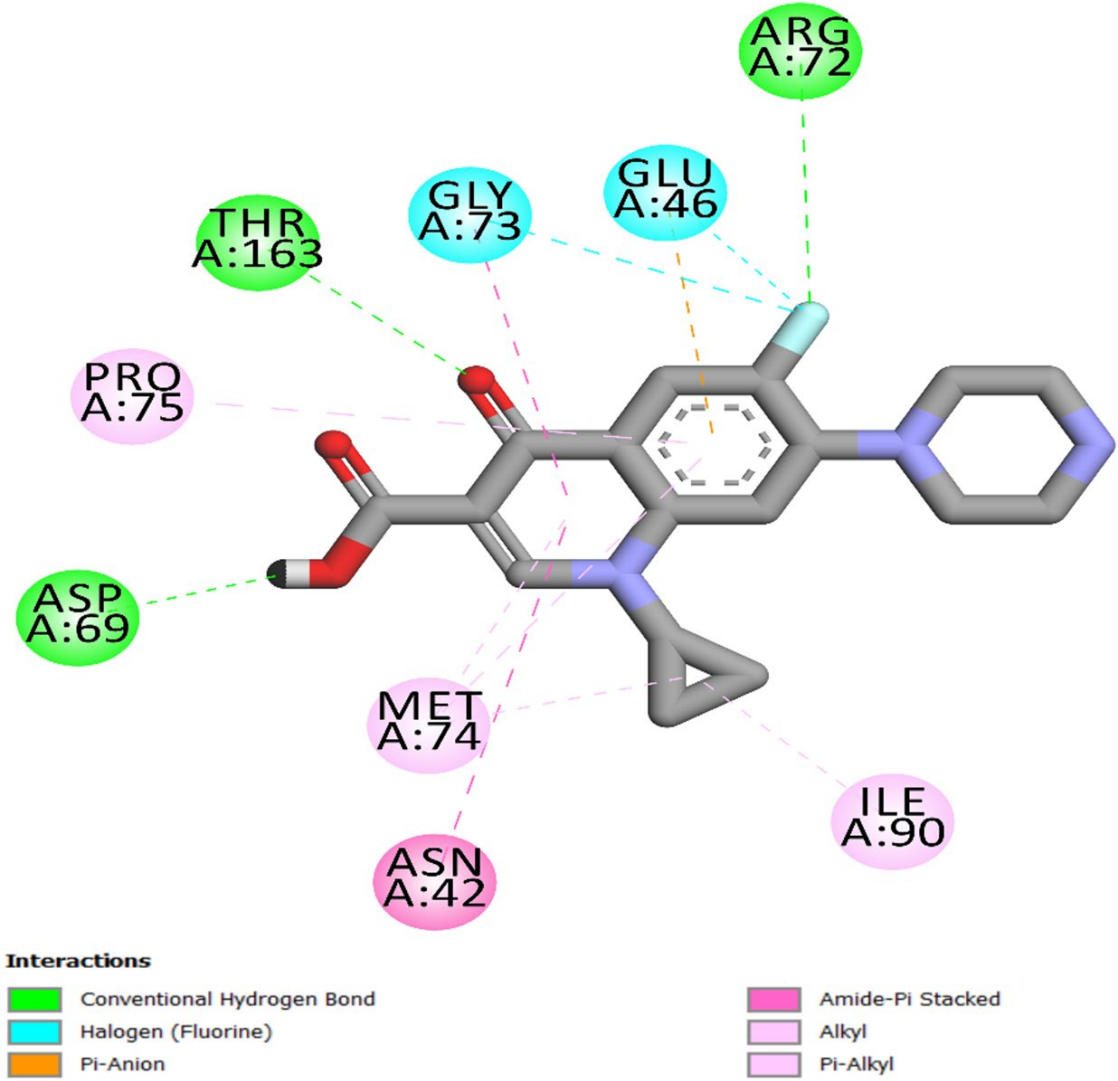
Two-dimensional docking representation models of Ciprofloxacin within the binding site of *E. coli* Topoisomerase IV protein.

In our investigation of *E. coli* topoisomerase IV, we integrated the crystallographic structure of its ligand complex (PDB ID: 3FV5)^52^ from the Protein Data Bank to provide a platform for computer modelling. Ciprofloxacin was docked back into the active site of *E. coli* topoisomerase IV to test the accuracy of our methods. This process provided an S score of −7.60 kcal/mol, demonstrating the methodological precision of our docking strategy. The identification of strong hydrogen bonding between the ligand and the amino acid residues Asp69, Thr163, and Arg72 was critical to this validation, as it defined essential interactions that enable ligand binding. Furthermore, ciprofloxacin formed two halogen (fluorine) bonds with residues Gly73 and Glu46, as well as a network of hydrophobic interactions with Pro75, Met74, Asn42, and Ile90 (Fig. 7).

**Fig. 8:**
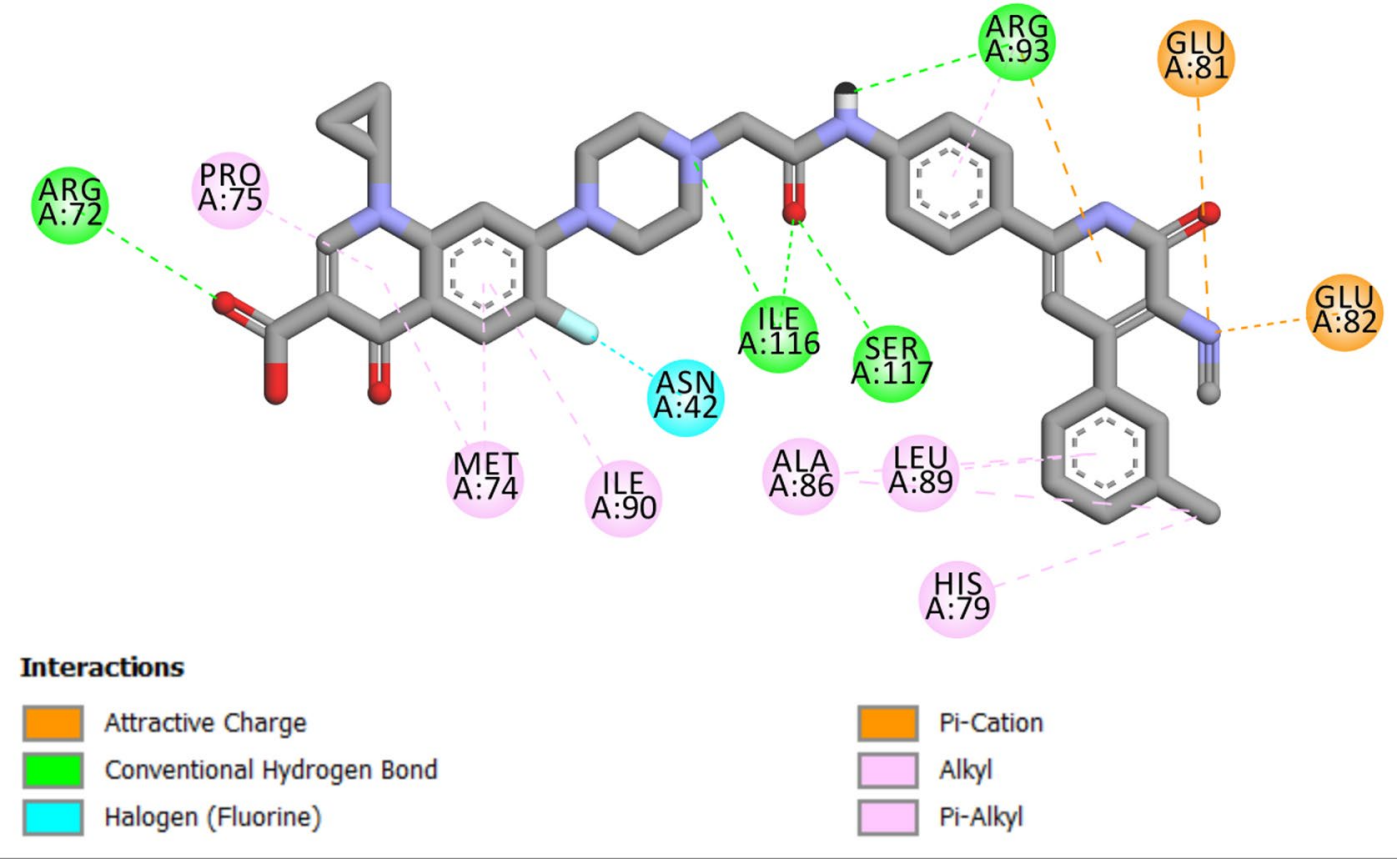
2D Map of **6 g** within the binding site of *E. coli* Topoisomerase IV protein.

Compound **6 g** demonstrated the greatest in vitro efficacy against *E. coli topoisomerase IV*,* achieving* a docking score of −9.00 kcal/mol. Compound **6 g** displayed notable binding properties to Coli topoisomerase IV. Numerous interactions were identified, particularly with the carboxyl group of **6 g**. This process enabled the formation of a hydrogen bond with the essential amino acid residue Arg72, highlighting the affinity for the active site. A further analysis of **6 g** binding indicated the significant involvement of its acetamido group, which formed a network of hydrogen bonds with the amino acid residues Ile116, Ser117, and Arg93. The 3-cyanopyridine ring of **6 g** engaged in pi-stacking interactions with residues Glu81 and Glu82, demonstrating a complicated binding interplay. Additionally, **6 g** engaged in a halogen (fluorine) bond with the Asn42 residue, as well as a network of hydrophobic interactions, as depicted in Fig. 8.

**Fig. 9:**
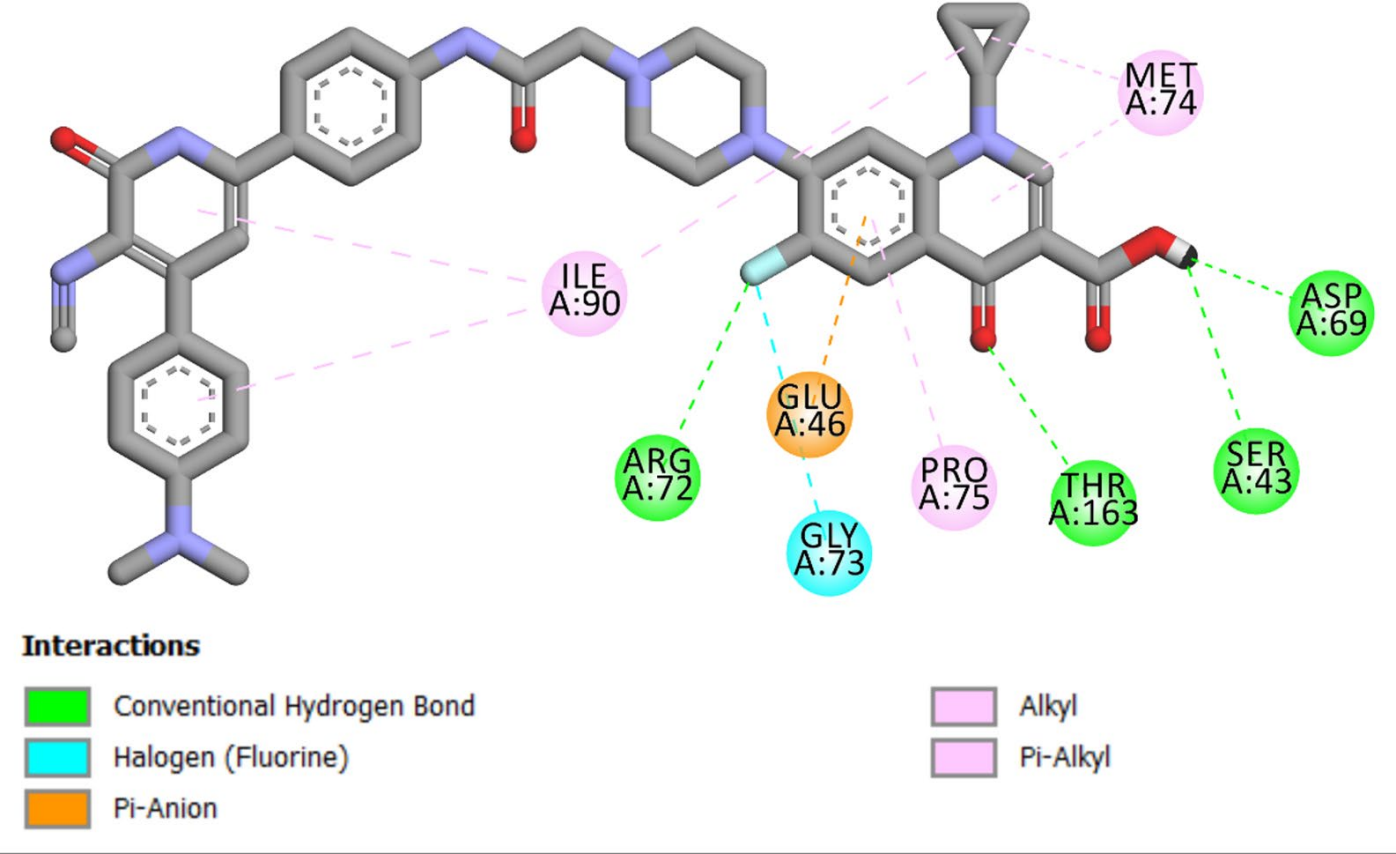
2D Map of **6 L** within the binding site of *E. coli* Topoisomerase IV protein.

The docking analysis of Compound **6 L** was defined by the lack of certain crucial hydrophobic and pi-stacked interactions that were essential for the increased binding affinity of **6 g** to the active site. The lack of these interactions and the inferior docking score of **6 L** (−8.25 kcal/mol) relative to **6 g** (−9.00 kcal/mol) indicate that **6 L** exhibits lower inhibitory efficacy against Topoisomerase IV than **6 g**, as illustrated in Fig. 9.

### ADME analysis

A preliminary in silico ADMET prediction was performed using the ADMET lab 2.0 online tool^54,55^ to assess the pharmacokinetic and drug-likeness properties of the synthesized Compounds (**6a**–**l**), as summarized in Table 5. All the compounds were evaluated for key parameters, including molecular weight, hydrogen bond acceptors (HBA), hydrogen bond donors (HBD), lipophilicity (LogP), and adherence to Lipinski’s Rule of Five (RO5). The molecular weights of the compounds ranged from 658.2 g/mol to 701.3 g/mol, exceeding the ideal threshold of 500 g/mol for optimal drug-like properties. On the other hand, most of the compounds met the necessary criteria for HBA (fewer than 10) and HBD (fewer than 5), suggesting that they may interact favorably with biological targets. The LogP values range from 4.27 to 5.38, indicating moderate lipophilicity, which is crucial for maintaining a balance between absorption and distribution. However, none of the compounds fully complied with Lipinski’s Rule of Five and possess relatively high molecular weights (658–737 g/mol) which may adversely affect their oral bioavailability and drug-likeness. These limitations suggest that the current compounds should be considered as preliminary lead structures rather than optimized drug candidates.

Additionally, the compounds were also evaluated for their gastrointestinal (GI) absorption potential, and all exhibited low GI absorption rates, typically below 0.02. The blood-brain barrier (BBB) permeability was low across all compounds, confirming their unlikely penetration into the central nervous system. The evaluation of cytochrome P450 (CYP) inhibition revealed important insights into the compounds’ metabolic profiles. Compounds like **6a**, **6e**, and **6 g** demonstrated low potential for CYP inhibition, indicating they are less likely to interact with the metabolism of other drugs, thereby reducing the risk of pharmacokinetic drug-drug interactions. On the other hand, compounds such as **6c** and **6j** showed moderate inhibition against specific CYP isoforms (e.g., CYP2C9), suggesting a potential for metabolic interactions with frequently co-administered drugs. Notably, Compound **6 L** demonstrated minimal inhibition across all CYP isoforms, reinforcing its favorable metabolic stability. In conclusion, while all the compounds showed promise in terms of their drug-likeness and pharmacokinetic properties, compound **6a** stood out with a relatively favorable profile, suggesting it may be the most suitable candidate for further development. Its moderate lipophilicity, lack of significant CYP inhibition, and low BBB permeability position as a potential lead for further optimization. However, the low gastrointestinal (GI) absorption observed for all compounds suggests the need for further improvements to enhance oral bioavailability. These improvements could include incorporating more polar functional groups to increase hydrophilicity or formulating the compounds into advanced drug delivery systems, such as nanoparticles or liposomes, to enhance bioavailability by improving solubility and, consequently, therapeutic effectiveness.

**Table 5:** In silico ADMET predictions for synthesized compounds **6a-l**.

Parameter	6a	6b	6c	6d	6e	6f	6g	6h	6i	6j	6k	6l
Molecular Weight	658.2	672.3	688.2	692.	736.1	676.2	692	703.2	718.3	686.3	703.2	701.3
HBA	11	11	12	11	11	11	11	14	13	11	14	12
HBD	3	3	3	3	3	3	3	3	3	3	3	3
LogP	4.62	5.01	4.70	5.25	5.38	4.80	5.24	4.53	4.27	5.28	4.56	4.61
Lipinski’s Rule	**No**	**No**	**No**	**No**	**No**	**No**	**No**	**No**	**No**	**No**	**No**	**No**
GI Absorption	0.01	0.01	0.01	0.01	0.01	0.01	0.01	0.01	0.01	0.01	0.01	0.02
BBB Permeant	0.01	0.01	0.01	0.01	0.01	0.01	0.01	0.01	0.01	0.01	0.01	0.03
CYP1A2 Inhibitor	0.06	0.06	0.05	0.09	0.07	0.06	0.08	0.05	0.03	0.05	0.04	0.06
CYP2C19 Inhibitor	0.302	0.276	0.2	0.31	0.41	0.21	0.39	0.32	0.21	0.33	0.21	0.16
CYP2C9 Inhibitor	0.83	0.60	0.52	0.50	0.51	0.50	0.70	0.70	0.73	0.79	0.44	0.52
CYP2D6 Inhibitor	0.02	0.005	0.004	0.02	0.01	0.01	0.02	0.005	0.002	0.006	0.002	0.004
CYP3A4 Inhibitor	0.08	0.09	0.11	0.08	0.07	0.08	0.10	0.13	0.13	0.12	0.09	0.10

With a molecular weight of 692 Da and a LogP of 5.24, compound **6 g** occupies a property space characterized by high lipophilicity and a relatively large molecular size. In a drug development context, these properties are known to significantly influence membrane permeability. While a molecular weight exceeding 600 Da typically hinders efficient diffusion across the complex bacterial cell wall, particularly through size-restricted porin channels, the observed potency of **6 g** against Gram-negative strains suggests a distinct entry mechanism. The high lipophilicity (5.24) likely facilitates a lipid-mediated uptake pathway (self-promoted uptake) through the lipopolysaccharide-rich outer membrane, allowing the compound to bypass porin-mediated restrictions. This favorable partitioning into and translocation across the outer membrane leads to high intracellular concentrations at the target site, effectively bridging the gap between potent enzymatic inhibition (IC_50_) and whole-cell antibacterial activity (MIC). This correlation between lipophilicity and Gram-negative penetration is a key feature of **6 g**’s profile, reinforcing its prioritization as a lead candidate for treating recalcitrant infections.

### Molecular dynamics (MD) simulations of 6 g against *E. Coli* DNA gyrase B

To elucidate the binding stability and time-dependent behavior of Compound **6 g** within the active site of *E. coli* DNA gyrase B, molecular dynamics (MD) simulations were performed over a 100-ns trajectory^56,57^, with ciprofloxacin serving as a reference ligand. Throughout the simulations, a series of structural and energetic parameters was monitored to evaluate the overall stability and conformational dynamics of the ligand–protein complex.

**Fig. 10:**
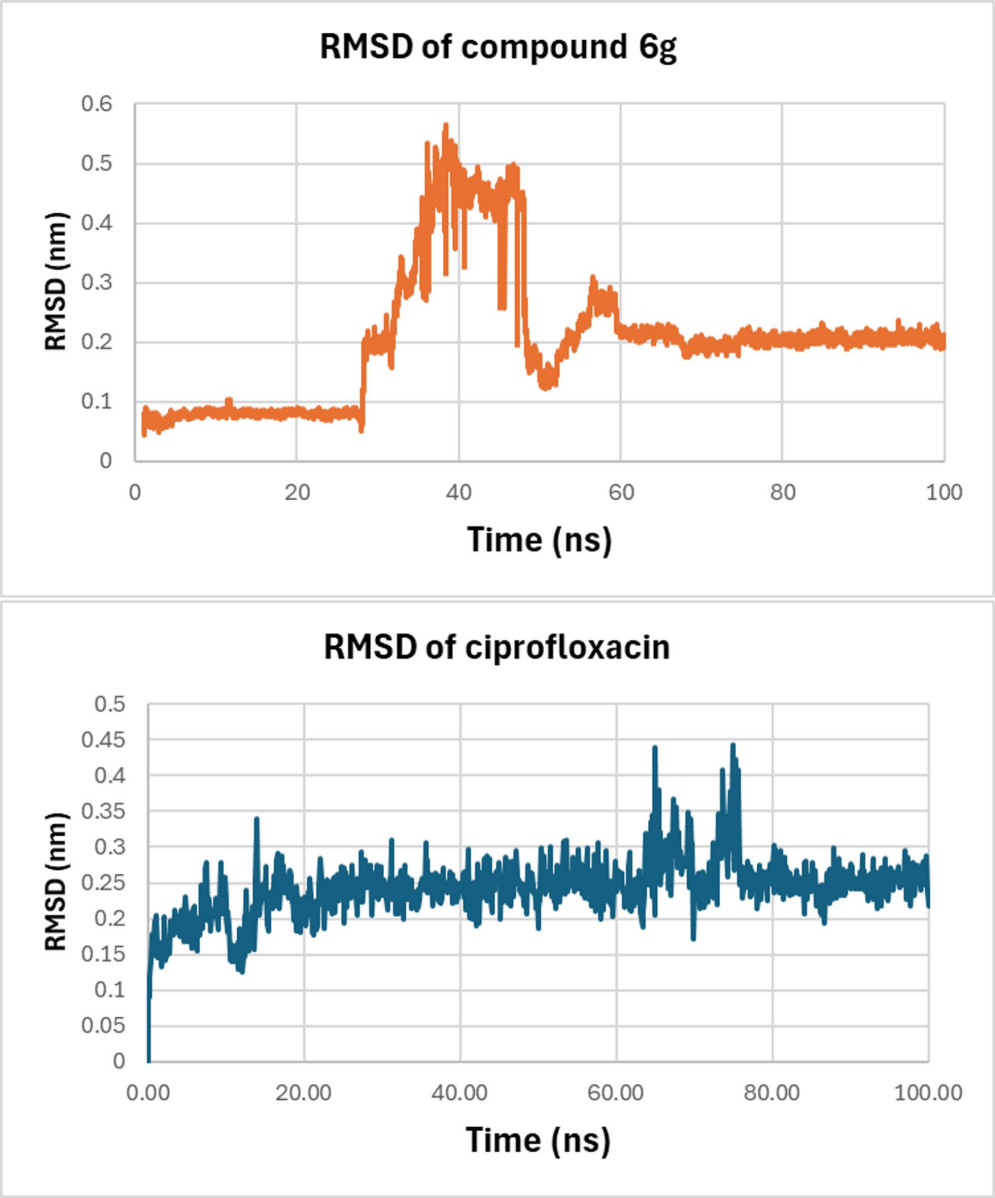
RMSD plot of *E. coli* DNA gyrase B–ligand complexes over a 100 ns. Compound **6 g** (orange) and ciprofloxacin (blue).

The Root Mean Square Deviation (RMSD) profile provides a comparative stability study of ciprofloxacin and Compound **6 g** within the binding pocket of *E. coli* DNA gyrase B over the 100-ns simulation. As illustrated in Fig. 10, ciprofloxacin displayed a consistently low RMSD throughout the trajectory, fluctuating within the narrow range of approximately 0.15–0.30 nm (0.084 ± 0.125), with only minor transient deviations around 60–80 ns. This stable pattern indicates that ciprofloxacin maintains a well-defined and persistent binding pose within the active site, with no evidence of destabilization. In contrast, Compound **6 g** exhibited an initial conformational adjustment phase during the early portion of the simulation, during which the ligand explored alternative orientations within the pocket. This reorientation phase, marked by a temporary rise in RMSD between 25 and 45 ns, enabled the ligand to adopt a more energetically favorable pose. Following this adaptive phase, Compound **6 g** stabilized markedly, maintaining a steady RMSD plateau of approximately 0.18–0.22 nm (0.185 ± 2.37 nm) for more than half of the simulation time, with minimal fluctuations thereafter. This sustained stabilization suggests that **6 g** ultimately established a robust and well-defined binding mode, demonstrating a strong capacity to equilibrate within the active site.

**Fig. 11:**
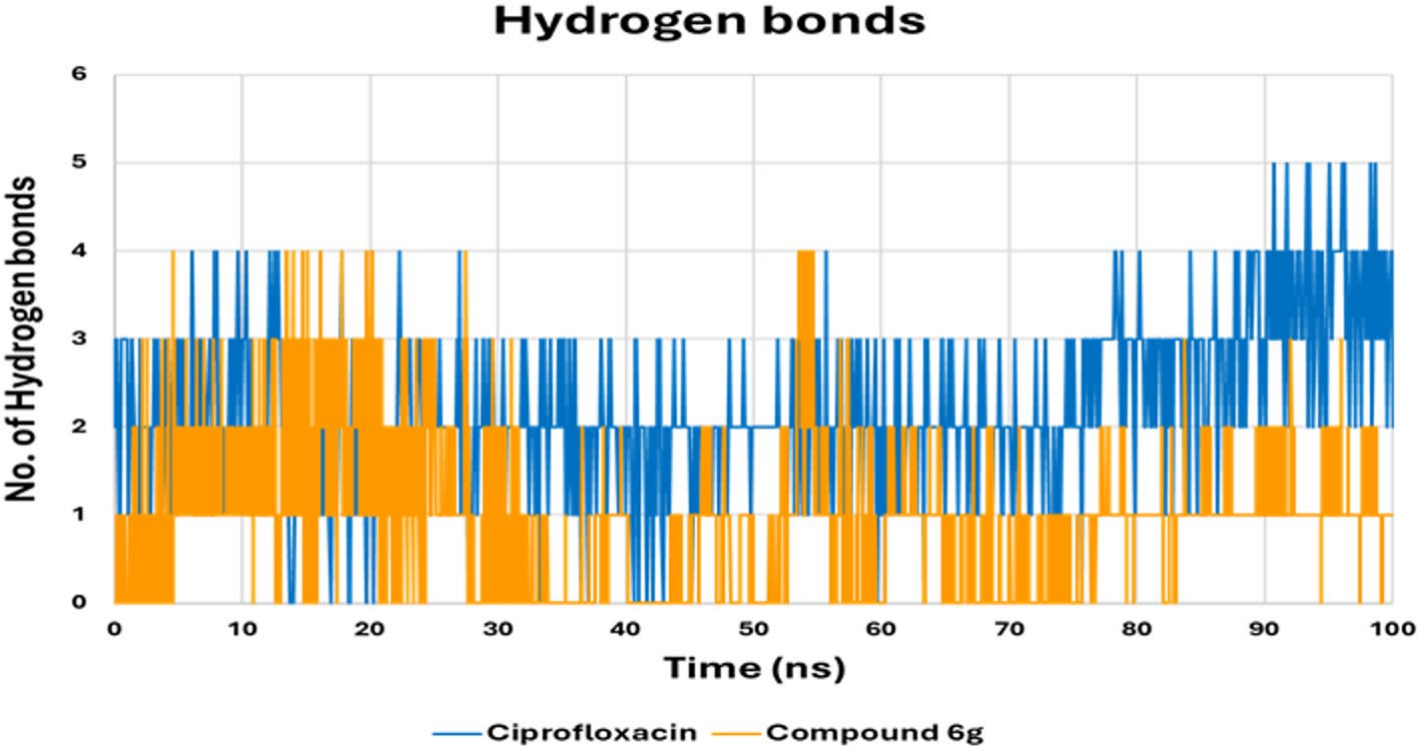
The hydrogen bond occupancy plot illustrates the number of hydrogen bonds established between the DNA gyrase B binding site and either **6 g** or ciprofloxacin during the simulation.

Hydrogen bonding analysis is a key element of ligand–protein binding specificity and affinity. As illustrated in Fig. 11, the hydrogen bonding profile of ciprofloxacin is highly dynamic, oscillating between 0 and 5 hydrogen bonds and frequently shifting between weakly bound states (0–1 HB) and moderately stable interactions (2–3 HB). Although there are periods of 4–5 hydrogen bond formations, these are interrupted by regular periods of reduced hydrogen bond formation, especially at about 30–40 ns and 80–90 ns, indicating irregular ligand binding to the active site (2.125 ± 1.140). In contrast, compound **6 g** exhibits a comparably stable hydrogen-bond interaction profile. At all instances throughout the simulation, compound **6 g** consistently maintained a hydrogen bond interaction of 2–4, with long periods of up to 5 hydrogen bonds (2.412 ± 0.844). Notably, compound **6 g** exhibits markedly fewer interruptions in hydrogen-bond formation compared with ciprofloxacin.

We conclude that, while ciprofloxacin maintains only a moderate, intermittently unstable hydrogen-bonding profile, compound **6 g** consistently exhibits more frequent, persistent, and structurally favorable hydrogen-bond interactions. This enhanced hydrogen-bonding capacity underscores the superior binding stability of compound **6 g** as a gyrase B inhibitor.

**Fig. 12:**
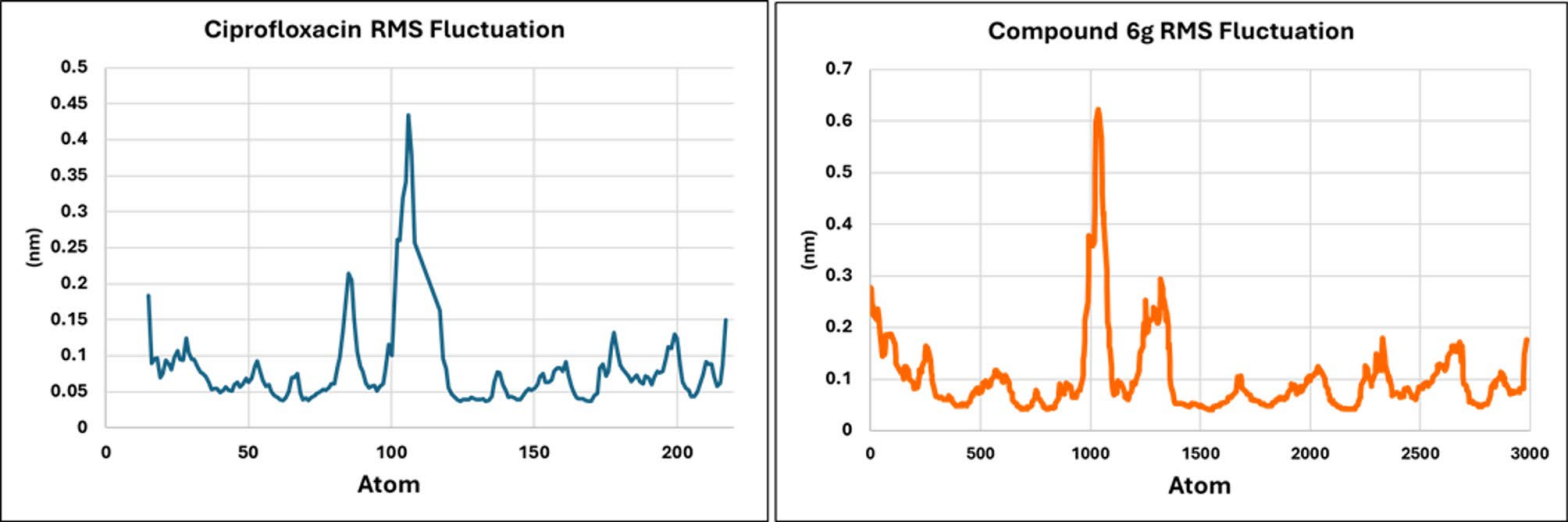
RMSF plot of *E.coli* DNA gyrase residues in complexes with compound **6 g** (orange) and ciprofloxacin (blue), illustrating the atomic flexibility of key binding site residues and overall stabilization of the ligand–receptor interactions.

Furthermore, the Root Mean Square Fluctuation (RMSF) profiles of the *E. coli* DNA gyrase B complexes with ciprofloxacin and compound **6 g** reveal marked differences in residue-level mobility, reflecting the distinct stabilizing capacities of the two ligands, as shown in Fig. 12.

In the ciprofloxacin-bound complex, most residues exhibit moderate flexibility, with RMSF values ranging from 0.05 to 0.20 nm (0.081 ± 0.057 nm), alongside a prominent peak reaching approximately 0.45 nm around residue index 100. Additional modest fluctuations appear between residue indices 70–120 and 180–220, consistent with the intrinsic dynamism of flexible protein segments. The elevated mobility in these regions suggests that ciprofloxacin imposes only limited structural restraints, allowing considerable conformational freedom in the vicinity of the binding site.

In contrast, the RMSF profile of the compound **6 g** complex demonstrates substantially reduced atomic fluctuations across the enzyme. Most residues remain below 0.10–0.12 nm (0.091 ± 0.056 nm), indicating a more rigid and stabilized protein environment. Although a pronounced fluctuation peak is observed around residues 1000–1100, corresponding to an intrinsically flexible loop present in both complexes, the critical residues forming the active site and immediate ligand-binding pocket exhibit markedly lower mobility in the **6 g**-bound system compared with the ciprofloxacin-bound complex. This enhanced stabilization strongly suggests that compound **6 g** binds to the active site with tighter affinity and superior complementarity, effectively restricting the conformational freedom of neighboring residues.

**Fig. 13:**
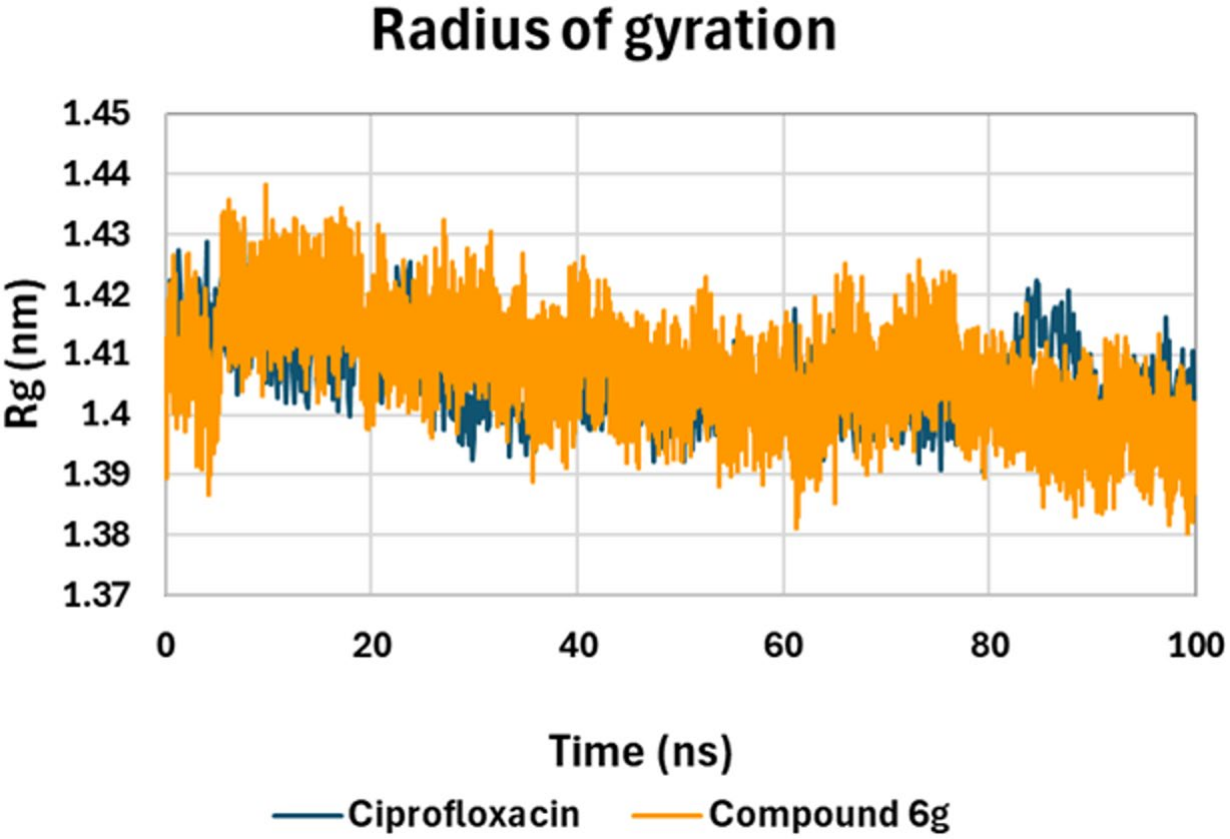
Rg plot of *E. coli* DNA gyrase residues in complexes with compound **6 g** (orange) and ciprofloxacin (blue), representing the compactness and structural stability of the receptor–ligand complexes over the course of the simulation.

On the other hand, Radius of Gyration (Rg) calculations were performed to determine the compactness and structural integrity of *E. coli* DNA Gyrase B in complex with ciprofloxacin and compound **6 g**, as shown in Fig. 13. Throughout the 100-ns simulation, both complexes maintained Rg values within a narrow and stable range, confirming that no large-scale unfolding or structural disruption occurred in either system. The ciprofloxacin complex fluctuated between approximately 1.40 and 1.42 nm (1.632 ± 0.008 nm), with noticeable short-term variations that indicate moderate structural oscillation of the protein. These fluctuations suggest that ciprofloxacin provides only partial stabilization, allowing the enzyme to undergo small expansions and contractions over time.

By contrast, in the **6 g** compound complex, the Rg values were marginally higher and more steadily ranged between 1.41 and 1.43 nm (1.638 ± 0.007 nm). It must be highlighted that in the **6 g** compound complex, the Rg values had less abrupt changes and a more uniform pattern across the simulation window. This stable profile indicates that compound **6 g** promotes greater structural compactness and, consequently, enhances the overall stability of the gyrase B protein complex.

**Fig. 14:**
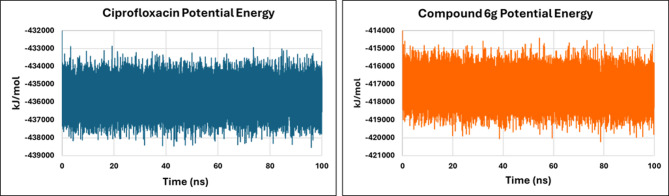
Potential energy plot of DNA gyrase–ligand complexes for ciprofloxacin (blue) and compound **6 g** (orange), illustrating the energy stability of the systems throughout the simulation.

Finally, to assess ligand stability and binding affinity within the DNA gyrase active site, the total potential energy of the system, encompassing bonded and non-bonded components, including van der Waals interactions, electrostatic forces, and covalent bonds, was examined as a principal measure of overall structural stability (Fig. 14). The ciprofloxacin-bound complex displayed potential energy values between approximately − 432,000 and − 439,000 kJ/mol, characterized by pronounced high-frequency fluctuations. This oscillatory pattern suggests that, while stable, the system does not achieve complete energetic stabilization, permitting recurrent adjustments in both bonded and non-bonded interactions. Conversely, the complex with compound **6 g** maintained a narrower, more consistent potential energy range (approximately − 414,000 to −421,000 kJ/mol). The **6 g**-bound system exhibited a smoother trajectory with fewer abrupt deviations, indicative of a more favorable and stable interaction profile. Collectively, the potential energy analysis indicates that compound **6 g** promotes a more stable and consistent binding milieu compared to ciprofloxacin. This finding correlates with the RMSD, RMSF, Rg, and hydrogen-bonding data, substantiating the superior binding stability and structural complementarity of **6 g** with the gyrase B active site.

## Structure–activity relationships

**Fig. 15:**
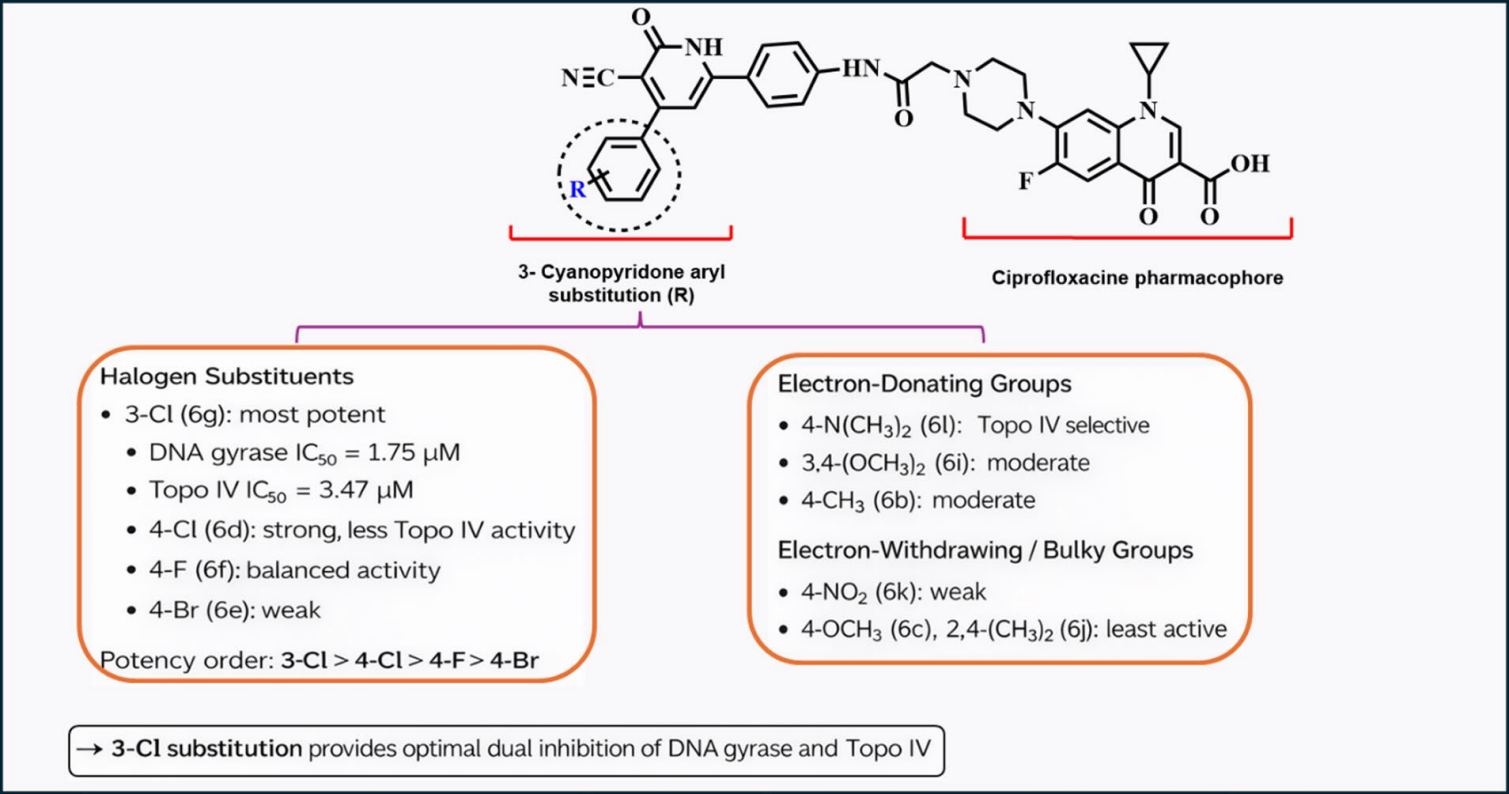
Structure-activity relationship of ciprofloxacin-3-cyanopyridone derivatives (**6a-l**).

Presented below is a detailed summary of the Structure-Activity Relationship (SAR) and related biological data (Fig. 15; Table 6):

(1) **Core Scaffold Stability**: The ciprofloxacin pharmacophore (C-6 fluorine, C-3 carboxylic acid, N-1 cyclopropyl, and C-7 piperazine) remained constant, providing a fixed baseline for evaluating the 3-cyanopyridone aryl substituents.
(2) **Lead Candidate Potency (6 g)**: The 3-chloro derivative (**6 g**) was identified as the most potent dual inhibitor, outperforming ciprofloxacin with an IC₅₀ of 1.75 µM for DNA gyrase and 3.47 µM for Topo IV (a 7.2-fold improvement in Topo IV inhibition).
(3) **Halogen Positional Effects**: The position of the halogen was critical; the 3-chloro (6 g) surpassed the 4-chloro (6d) and 4-fluoro (6f) analogues, establishing a clear potency hierarchy of 3-Cl > 4-Cl > 4-F > 4-Br.
(4) **Electronic Tolerance**: While electron-withdrawing halogens were optimal, the 4-N, N-dimethylamino derivative (6 L) showed significant Topo IV inhibition (IC₅₀ = 4.50 µM), suggesting that certain electron-donating groups are well-tolerated for enzyme engagement.
(5) **Detrimental Substitutions**: Strong electron-withdrawing groups (4-nitro, 6k) or sterically bulky/disubstituted rings (2,4-dimethyl, 6j) resulted in the lowest activity, with gyrase IC₅₀ values reaching as high as 161 µM.
(6) **Translational Limitations**: Despite high enzymatic potency, the series faces drug-likeness challenges, specifically high molecular weight (> 500 g/mol) and low predicted gastrointestinal absorption, necessitating further structural optimization.

**Table 6:** In vitro inhibitory activities of **6a–l** against DNA gyrase and Topo IV enzymes, highlighting the structure-activity relationship (SAR) of aryl substituents.

Compound ID	*R*	DNA Gyrase (IC_50_ = µM)	Topo IV (IC_50_ = µM)	SAR Observation
**6g**	3-Cl	**1.75**	**3.47**	**Lead Candidate**; most potent dual inhibitor.
**6d**	4-Cl	1.87	11.92	High gyrase potency; decreased Topo IV affinity.
**6f**	4-F	2.70	5.56	Balanced dual activity.
**6l**	4-N, N- Dimethyl	3.89	4.50	Electron-donor tolerated; strong Topo IV binding.
**6e**	4-Br	11.40		Significant loss in potency (4-Cl > 4-Br).
**6i**	3,4-dimethoxy	Intermediate	Intermediate	Moderate activity; positional tolerance.
**6b**	4-Me	Intermediate	Intermediate	Baseline activity for alkyl substitution.
**6k**	4-nitro	29.0	N.A.	Detrimental; strong electron-withdrawing effect.
**6j**	2,4-dimethyl	161.0	N.A.	Inactive; unfavorable steric bulk.
**Ciprofloxacin**	*Reference*	2.13	25.22	Reference standard for dual inhibition.

## Conclusion

Twelve compounds (**6a-l**) were synthesized by hybridizing ciprofloxacin with a pyridine moiety. The newly identified targets were examined for their inhibitory effect against DNA gyrase and topoisomerase IV. Compounds **6d**, **6f**, **6 g**, and **6 L** showed the highest activity in enzymatic assays. Compound **6 g** exhibited the highest potency among derivatives against both DNA gyrase and Topo IV, with IC_50_ values above those of ciprofloxacin for both enzymes. The antibacterial assays revealed that compound **6 g** displayed the greatest efficacy among the evaluated compounds, with MIC values marginally exceeding those of ciprofloxacin against *E. coli*, a gram-negative bacterium, while exhibiting roughly half the potency of ciprofloxacin against both *P. aeruginosa* and the gram-positive *S. aureus*. Compound **6 g** exhibits remarkable antibiofilm activity, inhibiting 96% of biofilms at the MIC. A comprehensive computational protocol utilizing molecular docking, ADMET prediction, and molecular dynamics simulations has been employed to examine the interaction of the synthesized compounds with *E. coli* DNA gyrase B and topoisomerase IV. Among all candidates, **6 g** had the best docking scores and the most extensive interaction network with key catalytic residues. Molecular dynamics simulations indicate that compound **6 g** generates a highly stable complex with DNA gyrase B. In silico ADMET profile indicated acceptable lipophilicity and metabolic safety; however, limited gastrointestinal absorption suggests constraints on oral bioavailability. Although compound **6 g** exhibited a comparatively favorable predicted ADMET profile among the synthesized derivatives, these predictions are based solely on computational estimation and were not intended to override the experimental biological findings. In contrast, collective enzymatic, antibacterial, docking, and molecular dynamics consistently identify compound **6 g** as the most potent and biologically relevant lead within this series. These data collectively suggest compound **6 g** as a potent lead scaffold for the development of antibacterial drug, warranting additional structural optimization and experimental confirmation.

### Future perspectives

While compound **6 g** demonstrates superior inhibition of Topo IV compared to ciprofloxacin at the molecular level, its comparable or slightly inferior MIC values likely reflect the multifaceted nature of bacterial cell entry. The higher molecular weight and lipophilicity of **6 g** compared to ciprofloxacin may lead to a slower rate of passive diffusion through the asymmetric Gram-negative outer membrane or the thick peptidoglycan layer of Gram-positive bacteria. Furthermore, as a novel scaffold, **6 g** may be subject to recognition by efflux pumps, which actively reduce intracellular drug concentrations. Future studies involving efflux pump inhibitors and membrane-permeabilizing agents will be essential to decouple these permeability barriers from the intrinsic biochemical potency of the scaffold. Additionally, The mechanistic proof-of-concept for these dual inhibitors is demonstrated in the present study. Future study will focus on evaluating their effectiveness against clinically relevant multidrug-resistant (MDR) pathogens, including MRSA and fluoroquinolone-resistant isolates.

In summary, we have identified compound **6 g** as the primary lead candidate for further drug development. Its prioritization is supported by its potent, dual inhibition of both DNA gyrase and Topoisomerase IV, a strategic advantage in the context of contemporary drug design. By simultaneously targeting two essential bacterial enzymes, compound **6 g** not only enhances antibacterial efficacy but also significantly increases the genetic barrier to the development of spontaneous resistance. These findings position **6 g** as a promising scaffold for the development of next-generation antibiotics capable of addressing the growing challenge of multi-drug resistant infections.

## Experimental

### Chemistry

**General details:** See Appendix A (Supplementary File).

Compounds **2** and **4** were synthesized based on earlier findings^58^.

#### General procedures for the Synthesis of Compounds 6a-l

A mixture including compound **4** (1 mmol), ethyl cyanoacetate (0.113 g, 1 mmol), ammonium acetate (0.154 g, 2 mmol), and benzaldehydes **5a-l** (1 mmol) was heated to 120–130 °C for 30 min. The reaction progress was monitored using thin-layer chromatography (TLC) employing a methanol: chloroform (1:9, v/v) solvent system. After cooling, the mixture was filtered, and pure **6a-l** was obtained by recrystallization of the crude product from absolute ethanol.

##### **7-(4-(2-((4-(5-cyano-6-oxo-4-phenyl-1**,**6-dihydropyridin-2-yl)phenyl)amino)−2-oxoethyl)piperazin-1-yl)−1-cyclopropyl-6-fluoro-4-oxo-1**,**4-dihydroquinoline-3-carboxylic acid** (**6a**)

Yellow powder; yield (0.559 g, 85%); m.p.: > 300 °C; ^1^H NMR (400 MHz, DMSO-*d*_6_) δ (ppm): 1.13–1.17 (m, 2 H, cyclopropyl-2 H), 1.27–1.31 (m, 2 H, cyclopropyl-2 H), 2.77 (s, 4 H, piperazinyl-H), 3.29 (s, 2 H, COCH_2_-N), 3.39 (s, 4 H, piperazinyl-H), 3.86 (s, 1H, cyclopropyl-H), 6.76 (s, 1H, pyridone-C_5_–H), 7.26 (s, 1H, Ar-H), 7.40 (t, *J* = 8.7 Hz, 2 H, Ar-H), 7.55 (s, 2 H, Ar-H), 7.77–7.87 (m, 4 H, Ar-H), 7.90 (d, *J* = 8.9 Hz, 2 H, Ar-H), 8.63 (s, 1H), 10.14 (s, 1H, CONH), 12.71 (s, 1H, pyridone-NH), 15.19 (s, 1H, COOH). ^13^C NMR (126 MHz, DMSO) δ 7.77, 13.11, 13.52, 30.81, 35.84, 49.57, 52.39, 61.51, 67.19, 72.15, 105.60, 106.72, 111.00, 115.67, 116.73, 118.53, 119.48, 119.54, 127.55, 127.87, 128.24, 128.83, 130.38, 136.22, 138.97, 141.39, 145.14, 147.94, 151.12, 152.01, 153.95, 159.70, 162.36, 166.00, 166.86, 168.86, 176.31. Anal. Calcd. For C_37_H_31_FN_6_O_5_ (658.23): C, 67.47; H, 4.74; N, 12.76. Found: C, 67.68; H, 4.90; N, 13.02.

##### **7-(4-(2-((4-(5-cyano-6-oxo-4-(p-tolyl)−1**,**6-dihydropyridin-2-yl)phenyl)amino)−2-oxoethyl)piperazin-1-yl)−1-cyclopropyl-6-fluoro-4-oxo-1**,**4-dihydroquinoline-3-carboxylic acid** (**6b**)

Yellow powder; yield (0. 537 g, 80%); m.p.: > 300 °C; ^1^H NMR (400 MHz, DMSO-*d*_6_) δ (ppm): 1.13 (s, 2 H, cyclopropyl-H), 1.26–1.28 (m, 2 H, cyclopropyl-H), 2.26 (s, 3 H, CH_3_), 2.73 (m, 4 H, piperazine-H), 3.23 (s, 2 H, COCH_2_-N), 3.36 (s, 4 H, piperazine-H), 3.85–3.89 (s, 1H, cyclopropyl-H), 6.67 (s, 1H, pyridone-C_5_–H), 7.15 (s, 3 H, Ar-H), 7.26 (d, 1H, Ar-H), 7.53–7.63 (m, 1H, Ar-H), 7.70–7.75 (m, 2 H, Ar-H), 7.86 (d, 1H, Ar-H), 7.92 (d, *J* = 8.5 Hz, 2 H), 8.61 (s, 1H, Ar-H), 9.98 (s, 1H, CONH), 12.67 (s, 1H, pyridone-NH), 15.17 (s, 1H, COO**H**). ^13^C NMR (126 MHz, DMSO) δ 8.09, 14.08, 21.18, 21.27, 31.37, 36.39, 43.94, 49.84, 52.80, 57.19, 61.94, 62.81, 67.46, 106.84, 107.23, 111.37, 111.56, 116.24, 119.36, 127.73, 128.23, 128.56, 129.28, 129.67, 133.16, 135.09, 136.66, 137.77, 138.03, 139.69, 141.35, 145.64, 148.48, 165.31, 166.47, 167.46, 169.01, 176.83. Anal. Calcd. For C_38_H_33_FN_6_O_5_ (672.25): C, 67.85; H, 4.94; N, 12.49. Found: C, 68.04; H, 5.12; N, 12.73.

##### 7-(4-(2-((4-(5-cyano-4-(4-methoxyphenyl)−6-oxo-1,6-dihydropyridin-2-yl)phenyl)amino)−2-oxoethyl)piperazin-1-yl)−1-cyclopropyl-6-fluoro-4-oxo-1,4-dihydro quinoline-3-carboxylic acid (6c)

Yellow powder; yield (0.550 g, 80%); m.p.: > 300 °C; ^1^H NMR (400 MHz, DMSO) δ 1.13–1.17 (m, 2 H, cyclopropyl-H), 1.25–1.31 (m, 2 H, cyclopropyl-H), 2.73 (s, 4 H, piperazine-H), 3.25 (s, 2 H, COCH_2_-N), 3.36 (s, 4 H, piperazine-H), 3.79 (s, 3 H, OCH_3_), 3.86–3.92 (m, 1H, cyclopropyl-H), 6.72 (s, 1H, pyridone-C_5_–H), 6.90 (d, J = 8.3 Hz, 1H, Ar-H), 7.06 (d, *J* = 8.4 Hz, 2 H, Ar-H), 7.53 (s, 1H, Ar-H), 7.66 (d, *J* = 8.3 Hz, 2 H, Ar-H), 7.72–7.80 (m, 2 H, Ar-H), 7.85 (d, *J* = 8.9 Hz, 2 H, Ar-H), 8.61 (s, 1H, Ar-H), 10.08 (s, 1H, CONH), 12.64 (s, 1H, pyridone-NH), 15.19 (s, 1H, COOH). ^13^C NMR (126 MHz, DMSO) δ 8.07, 14.10, 36.29, 49.82, 52.78, 55.56, 55.92, 61.93, 111.36, 114.05, 114.47, 114.66, 117.84, 119.71, 127.72, 128.83, 130.45, 139.62, 141.65, 145.59, 148.49, 159.37, 161.45, 162.83, 166.50, 169.22. Anal. Calcd. For C_38_H_33_FN_6_O_6_ (688.24): C, 66.27; H, 4.83; N, 12.20. Found: C, 66.45; H, 5.98; N, 12.41.

##### 7-(4-(2-((4-(4-(4-chlorophenyl)−5-cyano-6-oxo-1,6-dihydropyridin-2-yl)phenyl) amino)−2-oxoethyl)piperazin-1-yl)−1-cyclopropyl-6-fluoro-4-oxo-1,4-dihydroquinoline-3-carboxylic acid (6d)

Yellow powder; yield (0. 553 g, 80%); m.p.: > 300 °C; 1 H NMR (400 MHz, DMSO) δ 1.15–1.24 (m, 2 H, cyclopropyl-2 H), 1.30–1.35 (m, 2 H, cyclopropyl-2 H), 2.76–2.81 (m, 4 H, piperazinyl-H), 3.30 (s, 2 H, COCH_2_-N), 3.42 (s, 4 H, piperazinyl-H), 3.83 (s, 1 H, cyclopropyl-H), 6.81 (s, 1 H, pyridone-C_5_–H), 7.30–7.49 (m, 1 H, Ar-H), 7.58 (d, *J* = 7.3 Hz, 1 H, Ar-H), 7.67 (s, 1 H, Ar-H), 7.74 (d, *J* = 8.3 Hz, 1 H, Ar-H), 7.80 (d, *J* = 8.5 Hz, 3 H, Ar-H), 7.90 (d, *J* = 6.0 Hz, 1 H, Ar-H), 7.94 (d, *J* = 8.6 Hz, 3 H, Ar-H), 8.67 (s, 1 H, Ar-H), 10.14 (s, 1 H, CONH), 12.64 (s, 1 H, pyridone-NH), 15.22 (s, 1 H, COOH). ^13^C NMR (126 MHz, DMSO) δ 8.08, 26.94, 36.36, 49.79, 52.76, 61.92, 105.76, 106.78, 107.18, 111.33, 111.52, 117.00, 119.00, 119.06, 119.72, 124.54, 127.02, 129.00, 129.90, 130.87, 132.26, 135.78, 139.65, 141.94, 145.60, 145.68, 148.50, 151.57, 152.48, 154.47, 159.01, 162.49, 162.82, 166.45, 169.28, 176.79, 197.05. Anal. Calcd. For C_37_H_30_ClFN_6_O_5_ (693.13): C, 64.12; H, 4.36; N, 12.12. Found: C, 64.36; H, 4.49; N, 12.39.

##### 7-(4-(2-((4-(4-(4-bromophenyl)−5-cyano-6-oxo-1,6-dihydropyridin-2-yl)phenyl)amino)−2-oxoethyl)piperazin-1-yl)−1-cyclopropyl-6-fluoro-4-oxo-1,4-dihydro quinoline-3-carboxylic acid (6e)

Yellow powder; yield (0. 581 g, 79%); m.p.: > 300 °C; ^1^H NMR (400 MHz, DMSO*d6*) δ 1.13–1.17 (m, 2 H, cyclopropyl-2 H), 1.27 (s, 2 H, cyclopropyl-2 H), 2.73 (s, 4 H, piperazinyl-H), 3.25 (s, 2 H, COCH_2_-N), 3.36 (s, 4 H, piperazinyl-H), 3.65 (s, 1H, cyclopropyl-H), 6.78 (s, 1H, pyridone-C_5_–H), 7.40 (d, *J* = 113.0 Hz, 2 H, Ar-H), 7.63 (d, *J* = 7.9 Hz, 2 H, Ar-H), 7.73 (d, *J* = 7.9 Hz, 2 H¸ Ar-H), 7.81 (s, 2 H, Ar-H), 7.83–7.89 (m, 2 H, Ar-H). ^13^C NMR (126 MHz, DMSO) δ 8.08, 26.94, 36.36, 49.79, 52.76, 61.92, 105.76, 106.78, 107.18, 111.33, 111.52, 117.00, 119.00, 119.06, 119.72, 124.54, 127.02, 129.00, 129.90, 130.87, 132.26, 135.78, 139.65, 141.94, 145.60, 145.68, 148.50, 151.57, 152.48, 154.47, 159.01, 162.49, 162.82, 166.45, 169.28, 176.79, 197.05. Anal. Calcd. For C_37_H_30_BrFN_6_O_5_ (737.59): C, 60.25; H, 4.10; N, 11.39. Found: C, 60.49; H, 4.23; N, 11.65.

##### 7-(4-(2-((4-(5-cyano-4-(4-fluorophenyl)−6-oxo-1,6-dihydropyridin-2-yl)phenyl)amino)−2-oxoethyl)piperazin-1-yl)−1-cyclopropyl-6-fluoro-4-oxo-1,4-dihydro quinoline-3-carboxylic acid (6f)

Yellow powder; yield (0. 540 g, 80%); m.p.: > 300 °C; ^1^H NMR (400 MHz, DMSO) δ 1.13–1.17 (m, 2 H, cyclopropyl-2 H), 1.27–1.31 (m, 2 H, cyclopropyl-2 H), 2.77 (s, 4 H, piperazinyl-H), 3.29 (s, 2 H, COCH_2_-N), 3.39 (s, 4 H, piperazinyl-H), 3.86 (s, 1H, cyclopropyl-H), 6.76 (s, 1H, pyridone-C_5_–H), 7.26 (s, 1H, Ar-H), 7.40 (t, *J* = 8.7 Hz, 2 H, Ar-H), 7.55 (s, 1H, Ar-H), 7.77–7.87 (m, 4 H, Ar-H), 7.90 (d, *J* = 8.9 Hz, 2 H, Ar-H), 8.63 (s, 1H), 10.14 (s, 1H, CONH), 12.71 (s, 1H, pyridone-NH), 15.19 (s, 1H, COOH). ^13^C NMR (126 MHz, DMSO) δ 8.08, 26.94, 36.36, 49.79, 52.76, 61.92, 105.76, 106.78, 107.18, 111.33, 111.52, 117.00, 119.00, 119.06, 119.72, 124.54, 127.02, 129.00, 129.90, 130.87, 132.26, 135.78, 139.65, 141.94, 145.60, 145.68, 148.50, 151.57, 152.48, 154.47, 159.01, 162.49, 162.82, 166.45, 169.28, 176.79, 197.05. Anal. Calcd. For C_37_H_30_F_2_N_6_O_5_ (676.68): C, 65.67; H, 4.47; N, 12.42. Found: C, 65.92; H, 4.65; N, 12.61.

##### 7-(4-(2-((4-(4-(3-chlorophenyl)−5-cyano-6-oxo-1,6-dihydropyridin-2-yl)phenyl)amino)−2-oxoethyl)piperazin-1-yl)−1-cyclopropyl-6-fluoro-4-oxo-1,4-dihydro quinoline-3-carboxylic acid (6 g)

Yellow powder; yield (0.553 g, 80%); m.p.: > 300 °C; ^1^H NMR (400 MHz, DMSO) δ 1.13–1.17 (m, 2 H, cyclopropyl-2 H), 1.24–1.32 (m, 2 H, cyclopropyl-2 H), 2.73 (s, 4 H, piperazinyl-H), 3.25 (s, 2 H, COC**H**_**2**_-N), 3.36 (s, 4 H, piperazinyl-H), 3.77 (s, 1H, cyclopropyl-H), 6.83 (s, 1H, pyridone-C_5_–H), 7.56 (m, 3 H, Ar-H), 7.64 (d, *J* = 7.5 Hz, 1H, Ar-H), 7.77 (d, *J* = 11.7 Hz, 3 H, Ar-H), 7.84 (d, *J* = 13.2 Hz, 2 H, Ar-H), 7.88 (d, *J* = 8.2 Hz, 2 H, Ar-H), 8.60 (s, 1H, Ar-H), 10.07 (s, 1H, CONH), 12.72 (s, 1H, pyridone-NH), 15.17 (s, 1H, COOH). ^13^C NMR (126 MHz, DMSO) δ 8.08, 26.78, 36.38, 49.79, 52.75, 61.94, 105.92, 106.81, 107.19, 111.35, 111.54, 116.90, 119.02, 119.08, 119.70, 127.55, 128.54, 129.05, 129.91, 130.65, 131.16, 132.33, 133.96, 138.63, 139.67, 141.97, 145.61, 145.69, 148.41, 152.50, 154.49, 158.59, 162.47, 166.45, 169.28, 176.82, 197.05. Anal. Calcd. For C_37_H_30_ClFN_6_O_5_ (693.13): C, 64.12; H, 4.36; N, 12.12. Found: C, 64.35; H, 4.51; N, 12.34.

##### 7-(4-(2-((4-(5-cyano-4-(3-nitrophenyl)−6-oxo-1,6-dihydropyridin-2-yl)phenyl)amino)−2-oxoethyl)piperazin-1-yl)−1-cyclopropyl-6-fluoro-4-oxo-1,4-dihydro quinoline-3-carboxylic acid (6 h)

Yellow powder; yield (0. 527 g, 75%); m.p.: 268–270 °C; ^1^H NMR (400 MHz, DMSO) δ 1.13–1.17 (m, 2 H, cyclopropyl-2 H), 1.24–1.32 (m, 2 H, cyclopropyl-2 H), 2.72 (s, 4 H, piperazinyl-H), 3.25 (s, 2 H, COCH_2_-N), 3.51 (s, 4 H, piperazinyl-H), 3.78 (s, 1H, cyclopropyl-H), 6.90 (s, 1H, pyridone-C_5_–H), 7.51 (s, 2 H, Ar-H), 7.76 (s, 2 H, Ar-H), 7.89 (s, 3 H, Ar-H), 8.11 (s, 1H, Ar-H), 8.34 (s, 1H, Ar-H), 8.47 (s, 1H, Ar-H), 8.60 (s, 1H, Ar-H), 10.08 (s, 1H, CONH), 12.72 (s, 1H, pyridone-NH), 15.15 (s, 1H, COOH). ^13^C NMR (126 MHz, DMSO) δ 8.08, 26.95, 36.31, 49.82, 52.78, 61.93, 91.17, 97.34, 105.62, 106.78, 111.36, 111.55, 117.40, 119.18, 119.69, 123.59, 125.20, 128.92, 129.91, 130.94, 132.33, 135.41, 138.45, 139.64, 141.75, 143.61, 145.56, 148.32, 148.51, 152.48, 154.46, 157.10, 162.85, 166.51, 169.25, 176.75, 197.08. Anal. Calcd. For C_37_H_30_FN_7_O_7_ (703.69): C, 63.15; H, 4.30; N, 13.93. Found: C, 63.04; H, 4.43; N, 14.18.

##### 7-(4-(2-((4-(5-cyano-4-(3,4-dimethoxyphenyl)−6-oxo-1,6-dihydropyridin-2-yl)phenyl)amino)−2-oxoethyl)piperazin-1-yl)−1-cyclopropyl-6-fluoro-4-oxo-1,4-dihydro quinoline-3-carboxylic acid (6i)

Yellow powder; yield (0.538 g, 75%); m.p.: > 300 °C; ^1^H NMR (400 MHz, DMSO) δ 1.12 (s, 2 H, cyclopropyl-2 H), 1.27 (m, 2 H, cyclopropyl-2 H), 2.72 (s, 4 H, piperazinyl-H), 3.25 (s, 2 H, COCH_2_-N), 3.36 (s, 4 H, piperazinyl-H), 3.60 (s, 1H, cyclopropyl-H), 3.79 (s, 6 H, Ph-(3,4-diOCH_3_)_2_), 6.74 (s, 1H, pyridone-C_5_–H), 7.06 (s, 1H, Ar-H), 7.27 (s, 1H, Ar-H), 7.50 (d, *J* = 7.8 Hz, 2 H, Ar-H), 7.76 (s, 3 H, Ar-H), 7.84 (s, 1H, Ar-H), 8.60 (s, 1H, Ar-H), 10.11 (s, 1H, CONH), 11.97 (s, 1H, pyridone-NH), 15.15 (s, 1H, COOH). ^13^C NMR (126 MHz, DMSO) δ 8.07, 26.94, 31.28, 36.30, 49.82, 52.77, 56.17, 61.92, 105.61, 106.78, 111.36, 111.54, 112.05, 112.33, 117.87, 119.18, 119.73, 121.87, 127.72, 128.87, 129.89, 132.32, 139.64, 141.69, 143.48, 145.63, 148.49, 149.03, 151.09, 152.46, 154.47, 159.63, 162.85, 163.38, 166.53, 169.24, 169.36, 176.77, 197.08. Anal. Calcd. For C_39_H_35_FN_6_O_7_ (718.74): C, 65.17; H, 4.91; N, 11.69. Found: C, 65.09; H, 5.18; N, 11.84.

##### 7-(4-(2-((4-(5-cyano-4-(2,4-dimethylphenyl)−6-oxo-1,6-dihydropyridin-2-yl)phenyl)amino)−2-oxoethyl)piperazin-1-yl)−1-cyclopropyl-6-fluoro-4-oxo-1,4-dihydro quinoline-3-carboxylic acid (6j)

Yellow powder; yield (0.548 g, 80%); m.p.: > 300 °C; ^1^H NMR (400 MHz, DMSO) δ 0.96 (s, 2 H, cyclopropyl-2 H), 1.13–1.17 (m, 2 H, cyclopropyl-2 H), 1.27 (s, 6 H, Ph(CH_3_)_2_), 2.72 (s, 4 H, piperazinyl-H), 3.25 (s, 2 H, COCH_2_-N), 3.36 (s, 4 H, piperazinyl-H), 3.60 (s, 1H, cyclopropyl-H), 6.74 (s, 1H, pyridone-C_5_–H), 7.32–7.36 (m, 1H, Ar-H), 7.59 (s, 2 H, Ar-H), 7.68 (d, *J* = 7.8 Hz, 2 H, Ar-H), 7.76 (s, 2 H, Ar-H), 7.87 (s, 2 H, Ar-H), 8.64 (s, 1H, Ar-H), 10.14 (s, 1H, CONH), 12.71 (s, 1H, pyridone-NH), 15.19 (s, 1H, COOH). ^13^C NMR (126 MHz, DMSO) δ 8.07, 14.66, 26.87, 26.94, 26.97, 36.36, 49.81, 52.75, 61.92, 61.96, 66.85, 79.64, 92.46, 106.78, 107.20, 111.35, 111.54, 112.20, 112.82, 118.09, 118.77, 119.16, 129.89, 132.32, 134.30, 139.64, 143.49, 145.60, 148.37, 152.50, 154.23, 154.62, 154.67, 163.99, 166.47, 169.34, 176.80, 197.04. Anal. Calcd. For C_39_H_35_FN_6_O_5_ (686.74): C, 68.21; H, 5.14; N, 12.24. Found: C, 68.45; H, 5.07; N, 12.46.

##### 7-(4-(2-((4-(5-cyano-4-(4-nitrophenyl)−6-oxo-1,6-dihydropyridin-2-yl)phenyl)amino)−2-oxoethyl)piperazin-1-yl)−1-cyclopropyl-6-fluoro-4-oxo-1,4-dihydro quinoline-3-carboxylic acid (6k)

Yellow powder; yield (0. 527 g, 75%); m.p.: 269–271 °C; ^1^H NMR (400 MHz, DMSO) δ 1.13–1.17 (m, 2 H, cyclopropyl-2 H), 1.27–1.31 (m, 2 H, cyclopropyl-2 H), 2.77 (s, 4 H, piperazinyl-H), 3.29 (s, 2 H, COCH_2_-N), 3.39 (s, 4 H, piperazinyl-H), 3.86 (s, 1H, cyclopropyl-H), 6.76 (s, 1H, pyridone-C_5_–H), 7.26 (s, 1H, Ar-H), 7.40 (t, *J* = 8.7 Hz, 2 H, Ar-H), 7.55 (s, 1H, Ar-H), 7.77–7.87 (m, 4 H, Ar-H), 7.90 (d, *J* = 8.9 Hz, 2 H, Ar-H), 8.63 (s, 1H, Ar-H), 10.14 (s, 1H, CONH), 12.71 (s, 1H, pyridone-NH), 15.19 (s, 1H, COOH). ^13^C NMR (126 MHz, DMSO) δ 8.11, 30.00, 36.42, 49.87, 52.81, 61.96, 66.87, 106.82, 111.51, 117.27, 119.20, 119.72, 123.64, 125.27, 127.86, 128.99, 129.91, 130.96, 135.43, 138.37, 139.64, 141.87, 145.62, 148.33, 152.35, 152.97, 154.54, 157.36, 163.47, 163.54, 166.49, 169.28, 176.81, 197.06. Anal. Calcd. For C_37_H_30_FN_7_O_7_ (703.69): C, 63.15; H, 4.30; N, 13.93. Found: C, 63.36; H, 4.41; N, 14.09.

##### 7-(4-(2-((4-(5-cyano-4-(4-(dimethylamino)phenyl)−6-oxo-1,6-dihydropyridin-2-yl)phenyl)amino)−2-oxoethyl)piperazin-1-yl)−1-cyclopropyl-6-fluoro-4-oxo-1,4-dihydro- quinoline-3-carboxylic acid (6 L)

Yellow powder; yield (0.560 g, 80%); m.p.: 188–190 °C; ^1^H NMR (400 MHz, DMSO) δ 1.21 (m, 2 H, cyclopropyl-2 H), 1.27–1.31 (m, 2 H, cyclopropyl-2 H), 2.73 (s, 4 H, piperazinyl-H), 3.03 (s, 6 H, N(CH_3_)_2_), 3.26 (s, 2 H, COCH_2_-N), 3.36 (s, 4 H, piperazinyl-H), 3.52 (s, 1H, cyclopropyl-H), 6.79 (s, 1H, pyridone-C_5_–H), 7.52 (d, *J* = 38.4 Hz, 1H, Ar-H), 7.76 (d, *J* = 8.4 Hz, 2 H, Ar-H), 7.89 (m, 5 H, Ar-H), 7.91 (s, 2 H, Ar-H), 8.05 (s, 2 H, Ar-H), 8.60 (s, 1H, Ar-H), 10.12 (s, 1H, CONH), 12.76 (s, 1H, pyridone-NH), 15.15 (s, 1H, COOH). ^13^C NMR (126 MHz, DMSO) δ 8.07, 14.66, 26.87, 26.94, 26.97, 36.36, 49.81, 52.75, 61.92, 61.96, 66.85, 79.64, 92.46, 106.78, 107.20, 111.35, 111.54, 112.20, 112.82, 118.09, 118.77, 119.16, 129.89, 132.32, 134.30, 139.64, 143.49, 145.60, 148.37, 152.50, 154.23, 154.62, 154.67, 163.99, 166.47, 169.34, 176.80, 197.04. Anal. Calcd. For C_39_H_36_FN_7_O_5_ (701.76): C, 66.75; H, 5.17; N, 13.97. Found: C, 66.62; H, 5.26; N, 14.19.

### Biology

#### DNA gyrase and topoisomerase IV inhibitory assays

The inhibitory effects of Compounds **6a-l** on DNA gyrase and topoisomerase IV were assessed by a supercoiling assay^38^. Inspiralis assay kits were employed to assess inhibitory actions, adhering to established methods. Five distinct inhibitor concentrations were used to derive the IC_50_ values, which were then computed using the GraphPad Prism 6.0 program. Three separate measurements were used to get the IC_50_ values, and the final results are mean values. For further information, see Appendix A.

#### Antibacterial assays

Using ciprofloxacin as the reference compound, the antibacterial activity of Compounds **6d**, **6f**, **6 g**, **6i**, and **6 L** was assessed using a twofold serial dilution method^44^. Dose-response tests were used to determine the MIC. The reported values are derived from at least two separate investigations, each of which had three duplicates for each concentration. Appendix A (Supplementary File) contains experimental details.

#### Cell viability assay

The MCF-10 A cell line was used to assess the effects of **6d** and **6 g** on viability. Cell viability was assessed using the MTT assay during a four-day incubation period on MCF-10 A cells at a dose of 50 µM for each compound examined^46^. For further information, see Appendix A.

#### Antibiofilm assay

Using the Microtiter plate assay for biofilm quantification, Compound **6 g**’s antibiofilm activity against *E. coli* was evaluated^49^. Three different concentrations were used in the assay: the first was equivalent to the MIC of **6 g** against *E. coli*, the second was equivalent to 1/2 MIC, and the third was equivalent to 1/4 MIC. For further information, see Appendix A.

## Supplementary Information

Below is the link to the electronic supplementary material.

Supplementary Material 1

## Data Availability

The data supporting this paper are incorporated within the Supplementary Information.
