# Supplementary material for: Design, synthesis, and antibacterial assessment of a new series of ciprofloxacin-based compounds as possible dual DNA gyrase/topoisomerase IV inhibitors
Source: Sci Rep. 2026 Apr 30;16:13911. doi: 10.1038/s41598-026-50106-z (PMC13133380; doi:10.1038/s41598-026-50106-z)
Supplement: Supplementary file 1 — Supplementary Material 1 [file 41598_2026_50106_MOESM1_ESM.docx]

**Design, synthesis, and antibacterial assessment of a new series of ciprofloxacin-based compounds as possible dual DNA gyrase/Topoisomerase IV inhibitors**

Lamya H. Al-Wahaibi^1^, Hayat Ali Alzahrani^2^, Stefan Bräse^3^***, Bahaa G. M. Youssif^4^***, Mohamed Hisham^5^

^1^Department of Chemistry, College of Sciences, Princess Nourah bint Abdulrahman University, Riyadh 11671, Saudi Arabia; ^2^Applied Medical Science College, Medical Laboratory Technology Department, Northern Border University, Arar, Saudi Arabia; ^3^Institute of Biological and Chemical Systems, IBCS-FMS, Karlsruhe Institute of Technology, 76131 Karlsruhe, Germany; ^4^Pharmaceutical Organic Chemistry Department, Faculty of Pharmacy, Assiut University, Assiut 71526, Egypt; ^5^Pharmaceutical Chemistry Department, Faculty of Pharmacy, Deraya University, Minia, Egypt.

**To whom correspondence should be addressed:*

**Bahaa G. M. Youssif**, Ph.D. Pharmaceutical Organic Chemistry Department, Faculty of Pharmacy, Assiut University, Assiut 71526, Egypt.

Tel.: +201044353895; E-mail address: [bgyoussif2@gmail.com](mailto:bgyoussif2@gmail.com)

**Stefan Bräse**

Institute of Biological and Chemical Systems, IBCS-FMS, Karlsruhe Institute of Technology, 76131 Karlsruhe, Germany. E-mail address: [braese@kit.edu](mailto:braese@kit.edu)


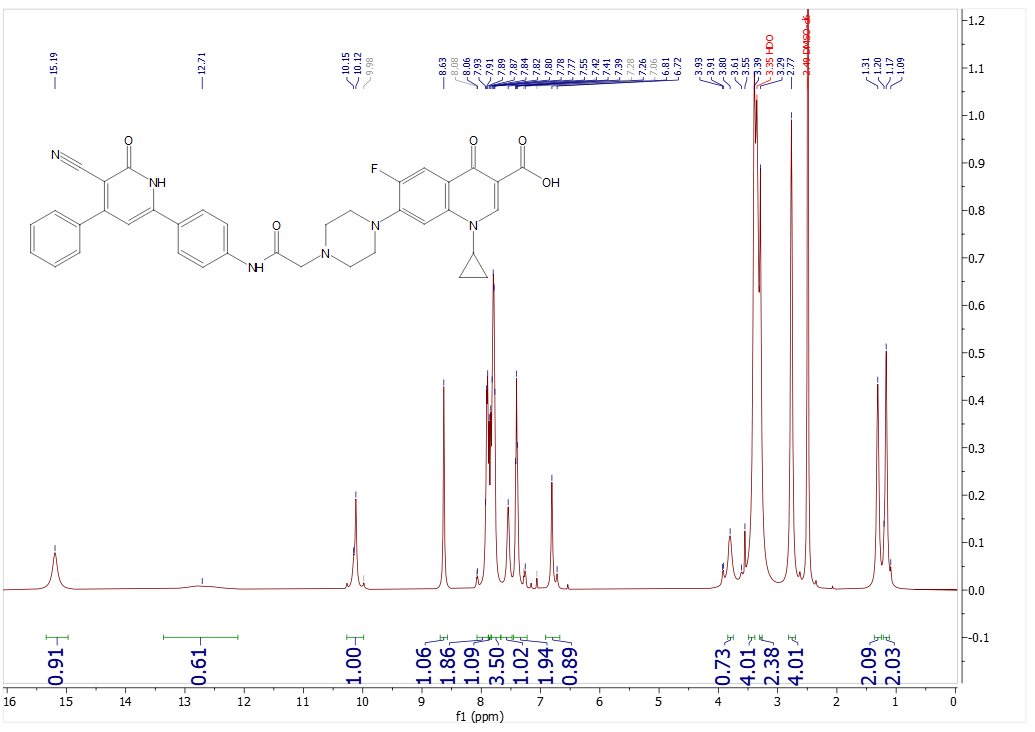


**Figure S1**. ^1^H NMR spectrum of compound **6a** in DMSO-*d*_6_ (400 MHz)


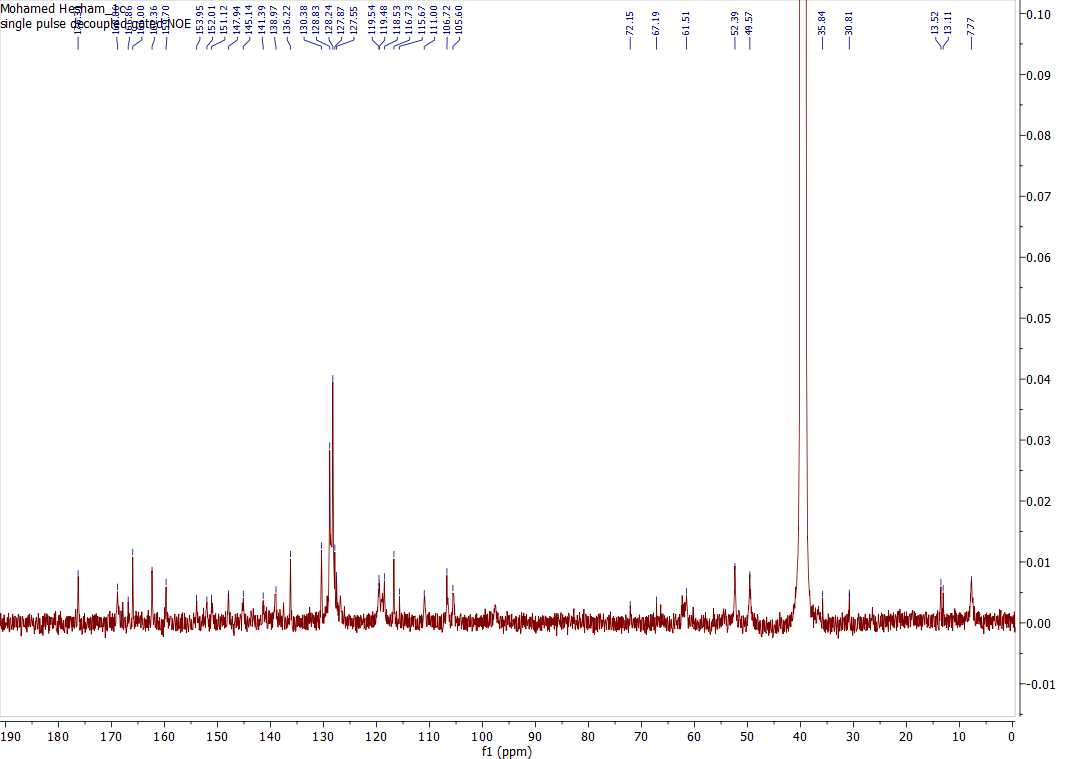


**Figure S2**. ^13^C NMR spectrum of compound **6a** in DMSO-*d*_6_ (125 MHz)

\


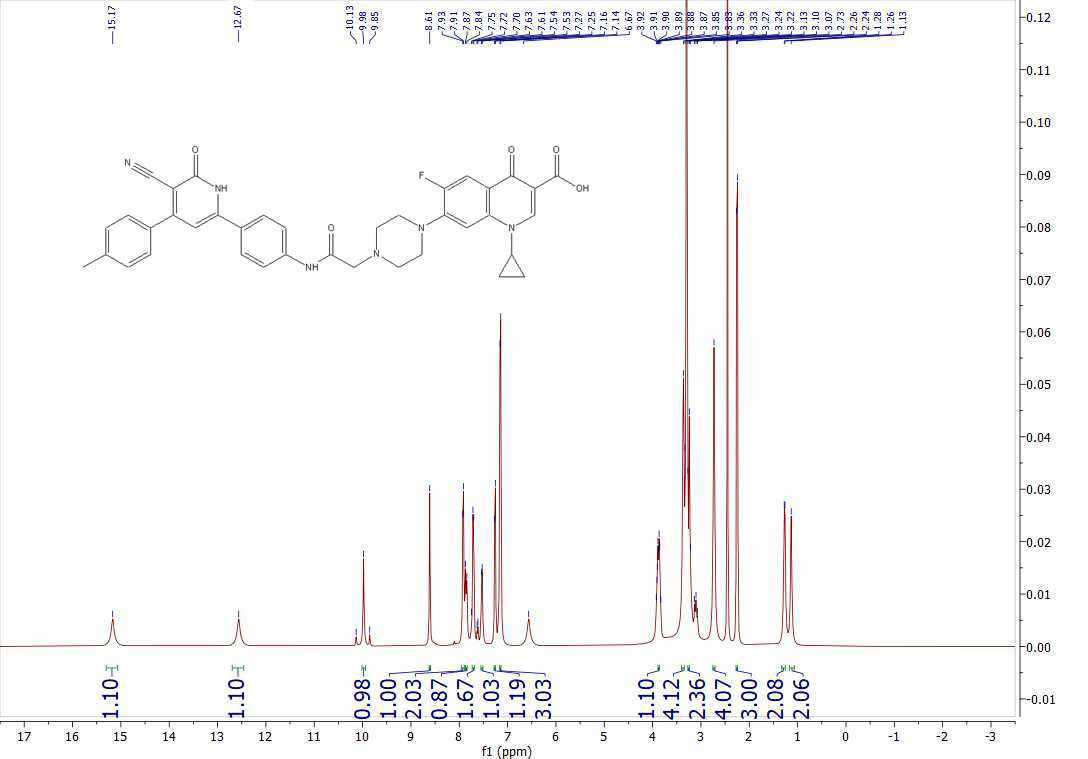


**Figure S3**. ^1^H NMR spectrum of compound **6b** in DMSO-*d*_6_ (400 MHz)


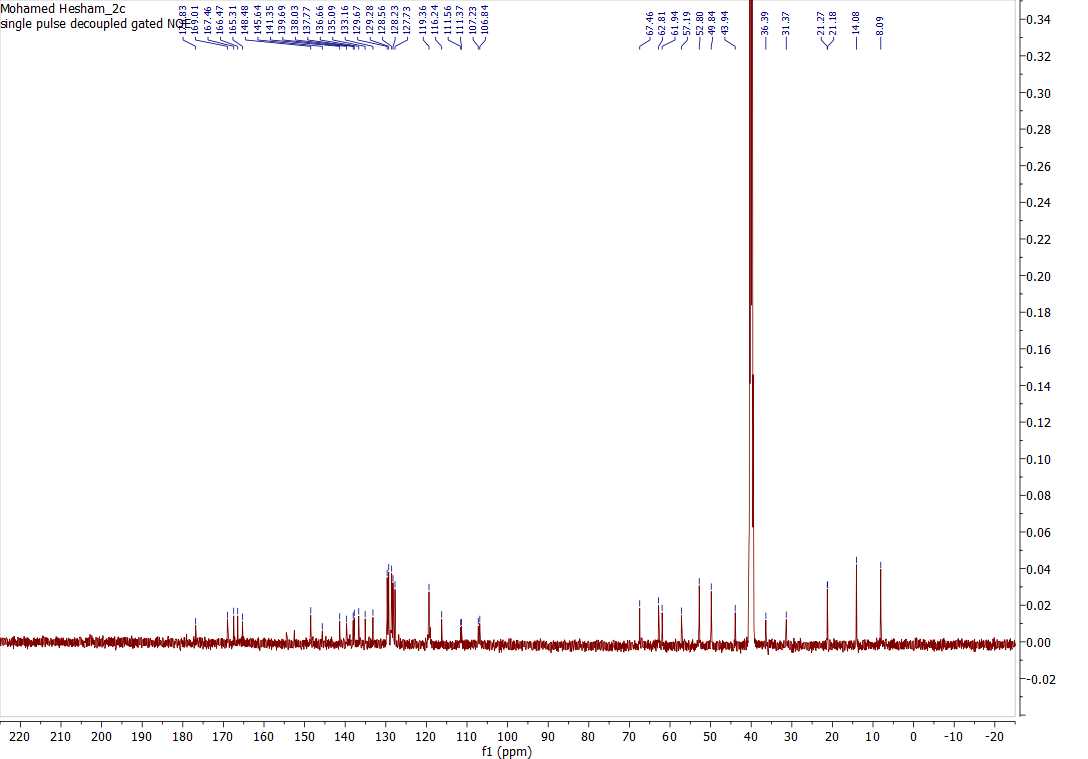


**Figure S4**. ^13^C NMR spectrum of compound **6b** in DMSO-*d*_6_ (125 MHz)


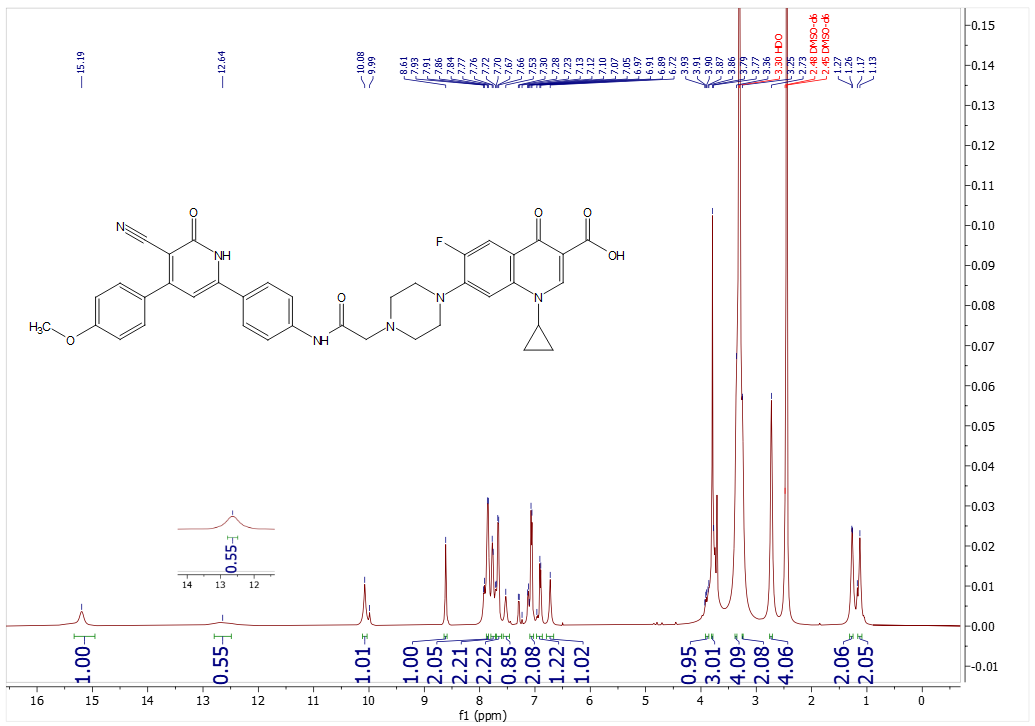


**Figure S5**. ^1^H NMR spectrum of compound **6c** in DMSO-*d*_6_ (400 MHz)


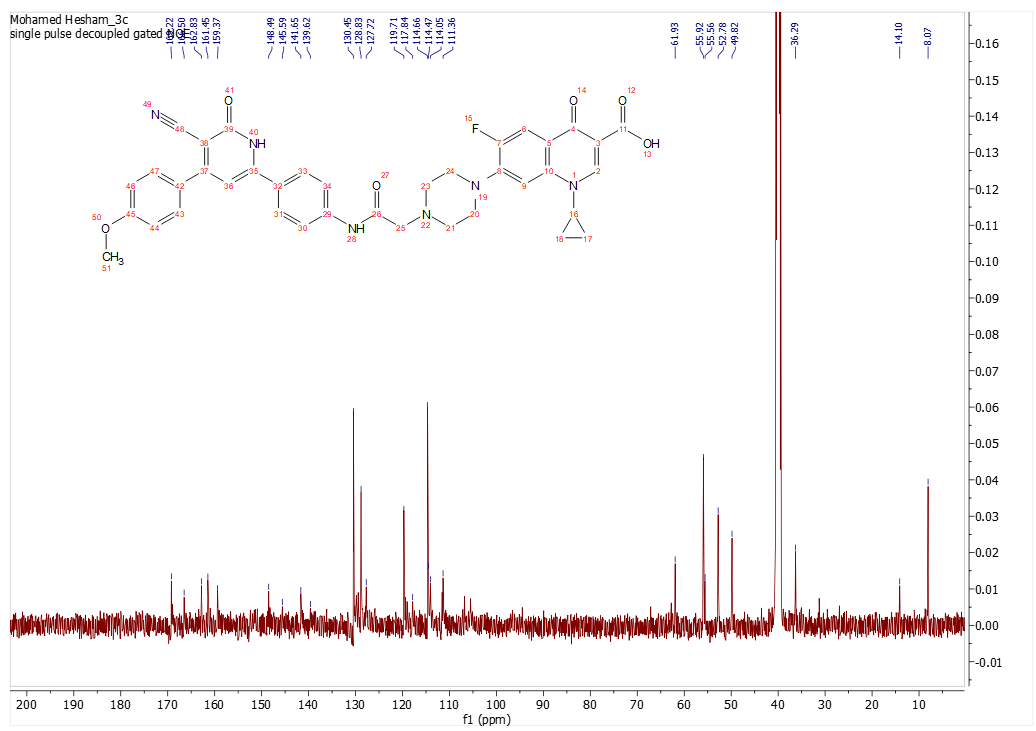


**Figure S6**. ^13^C NMR spectrum of compound **6c** in DMSO-*d*_6_ (125 MHz)


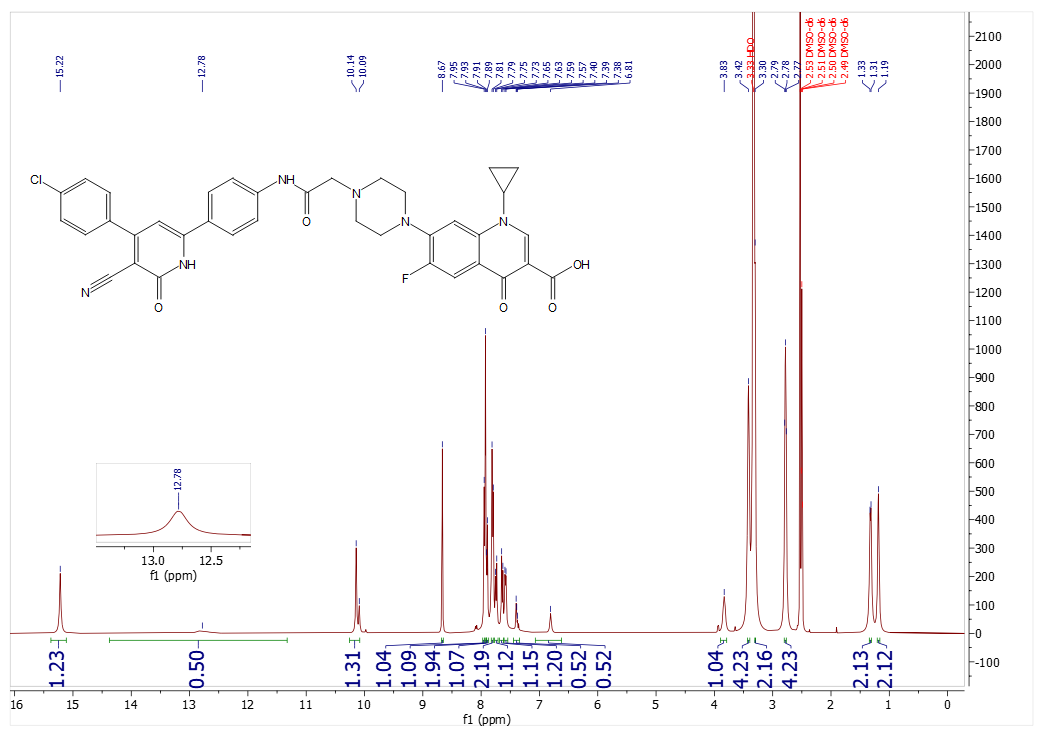


**Figure S7**. ^1^H NMR spectrum of compound **6d** in DMSO-*d*_6_ (400 MHz)


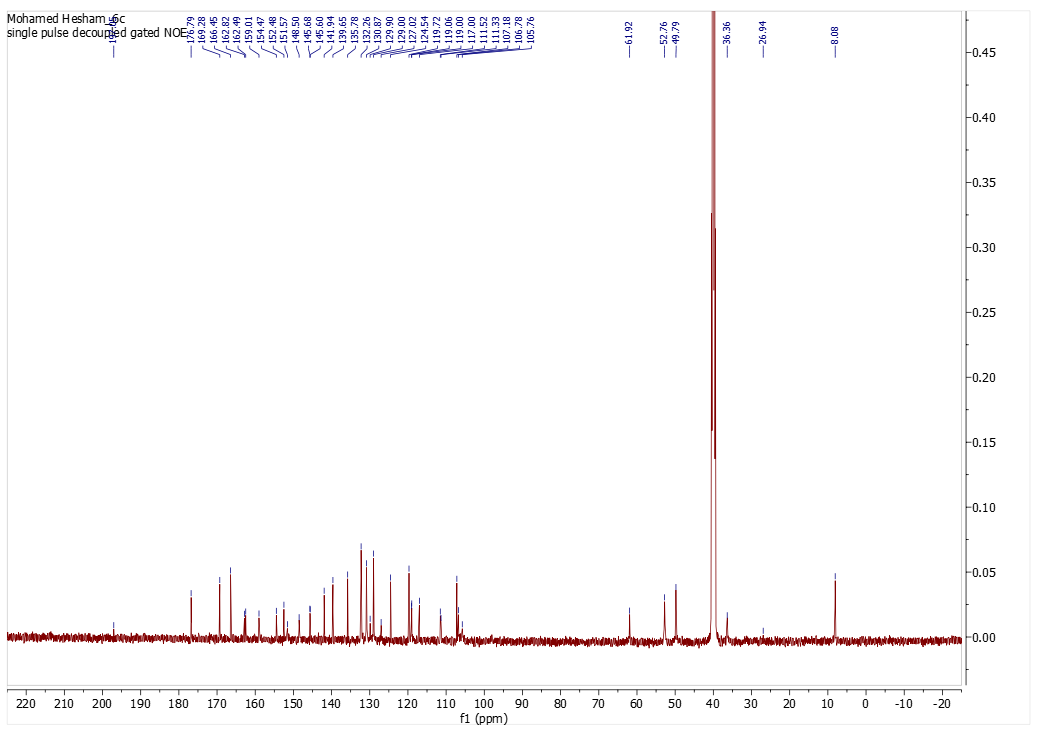


**Figure S8**. ^13^C NMR spectrum of compound **6d**in DMSO-*d*_6_ (125 MHz)


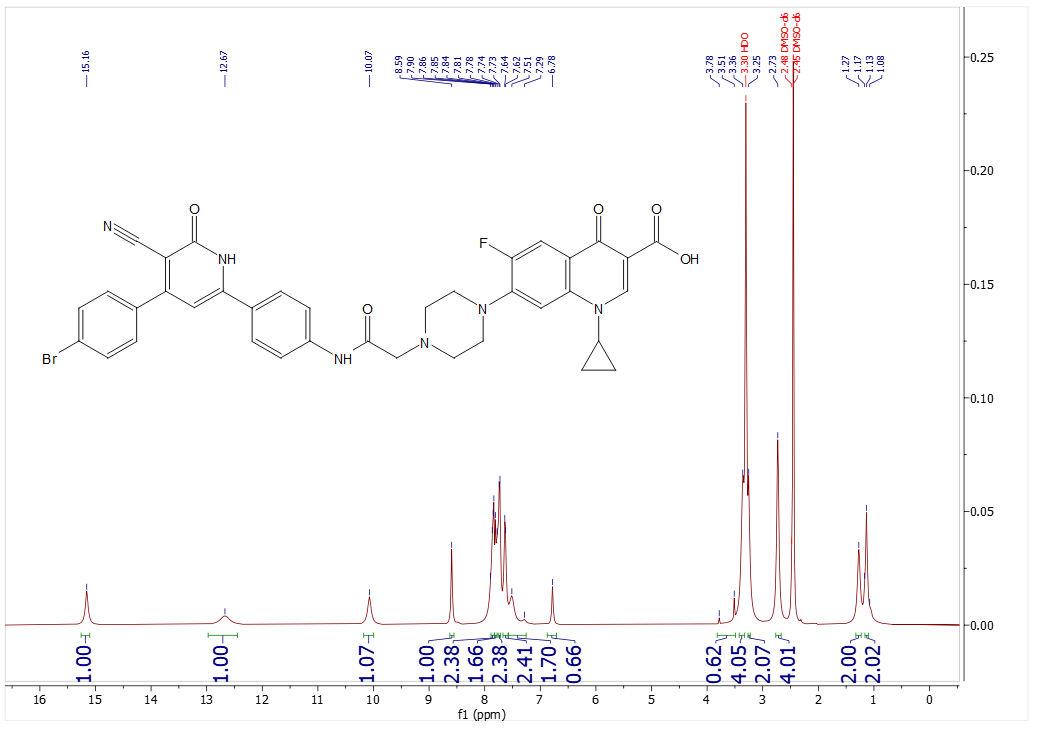


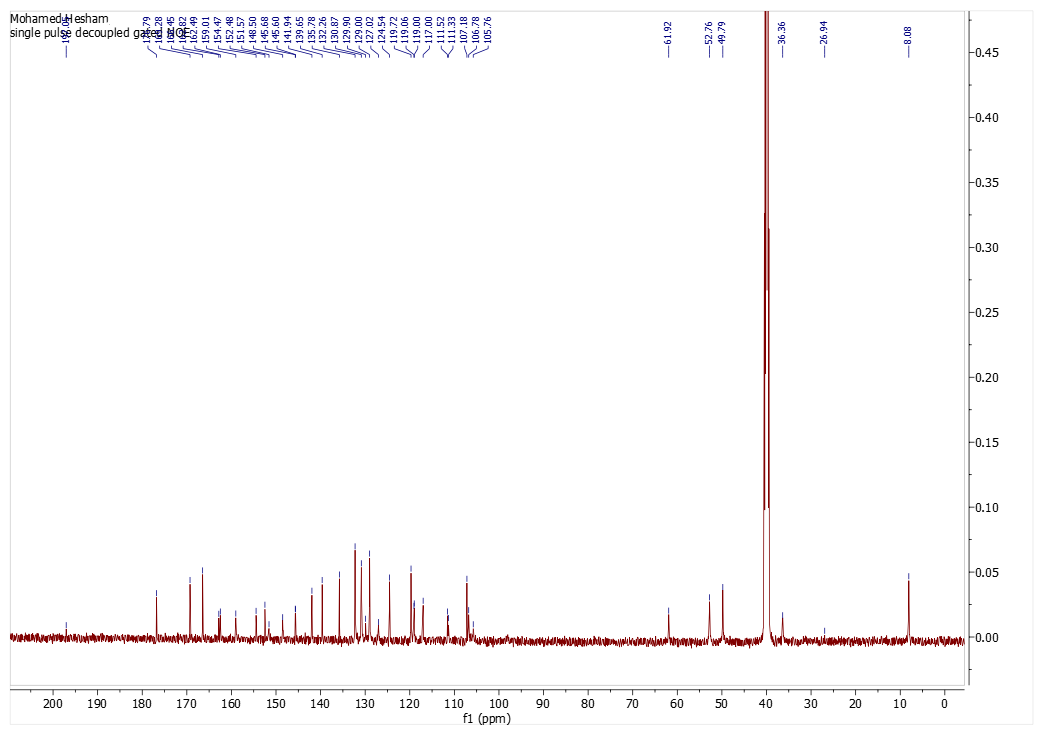
**Figure S9**. ^1^H NMR spectrum of compound **6e** in DMSO-*d*_6_ (400 MHz)


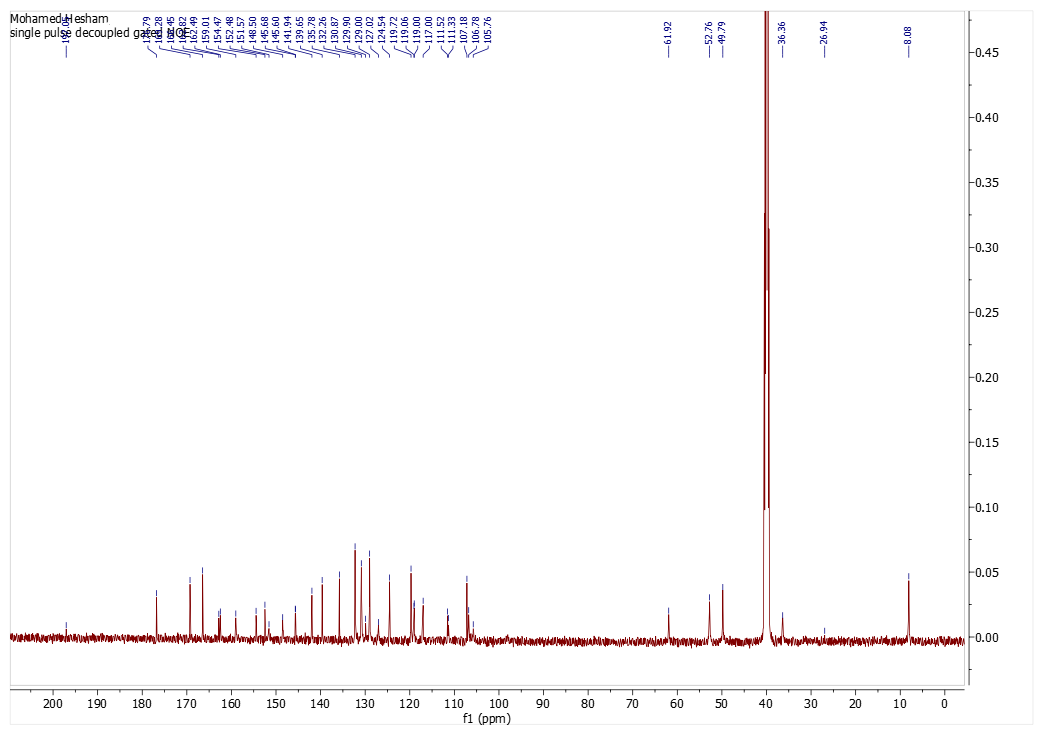


**Figure S10**. ^13^C NMR spectrum of compound **6e** in DMSO-*d*_6_ (125 MHz)


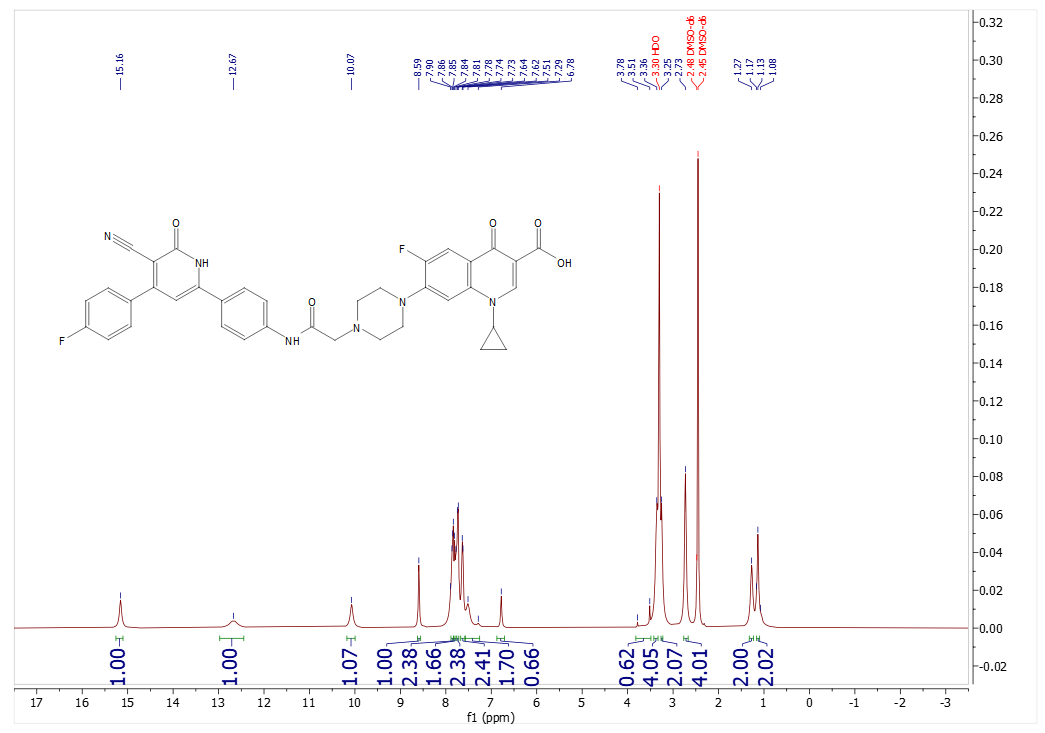
**Figure S11**. ^1^H NMR spectrum of compound **6f** in DMSO-*d*_6_ (400 MHz)


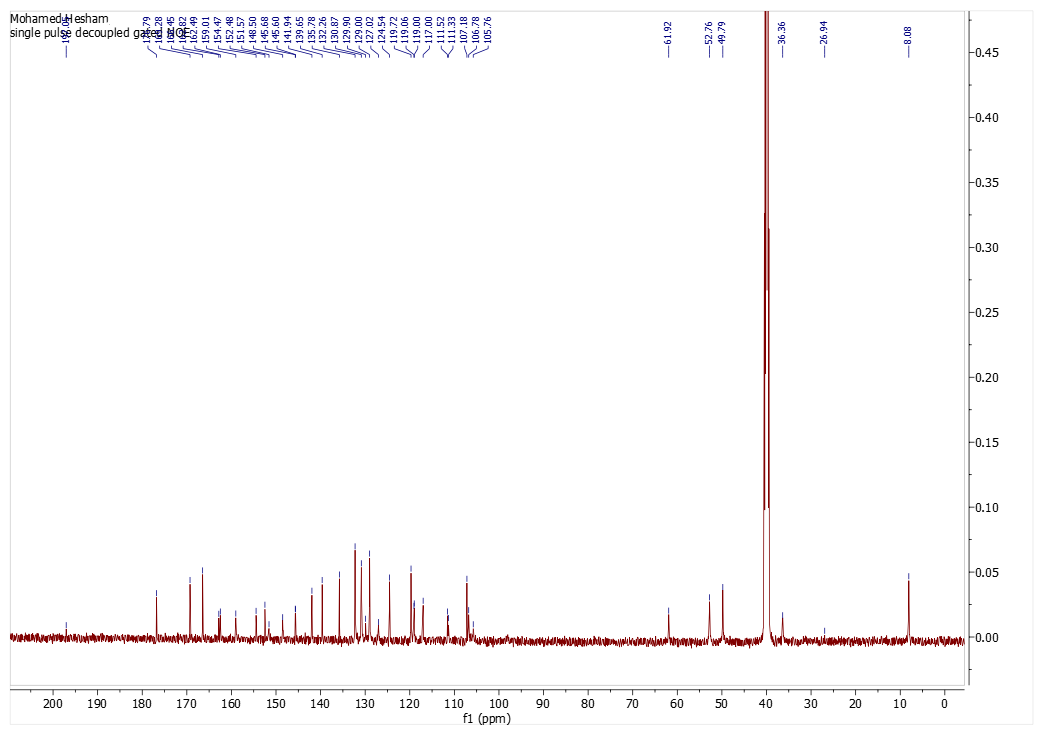


**Figure S12**. ^13^C NMR spectrum of compound **6f** in DMSO-*d*_6_ (125 MHz)


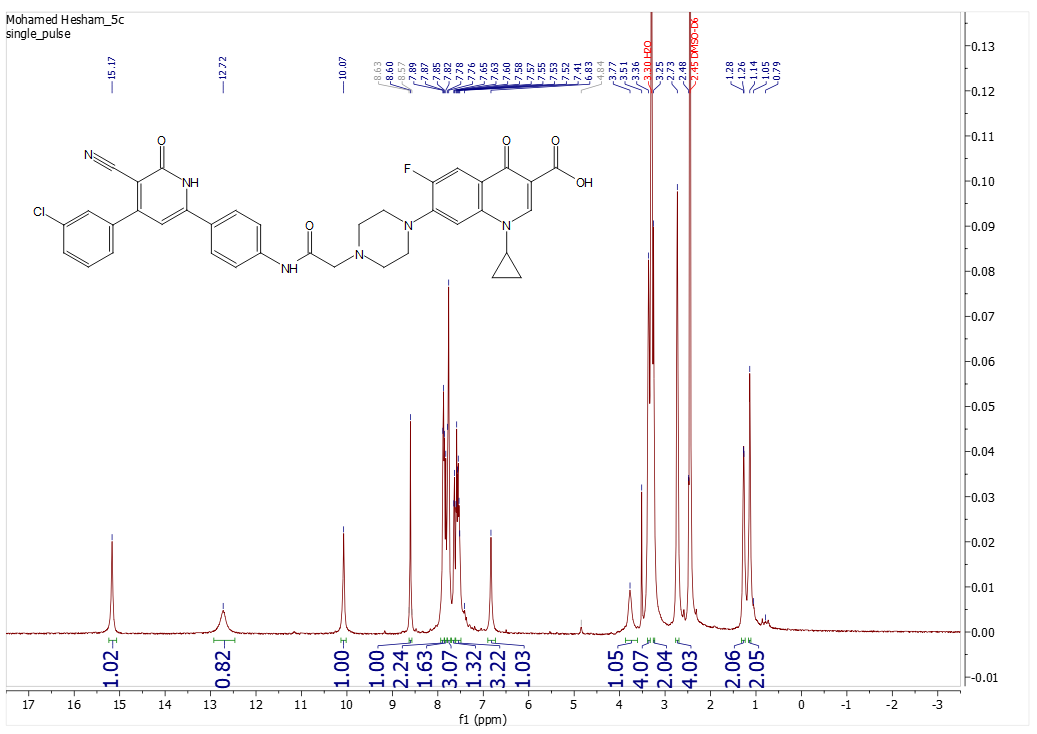


**Figure S13**. ^1^H NMR spectrum of compound **6g** in DMSO-*d*_6_ (400 MHz)


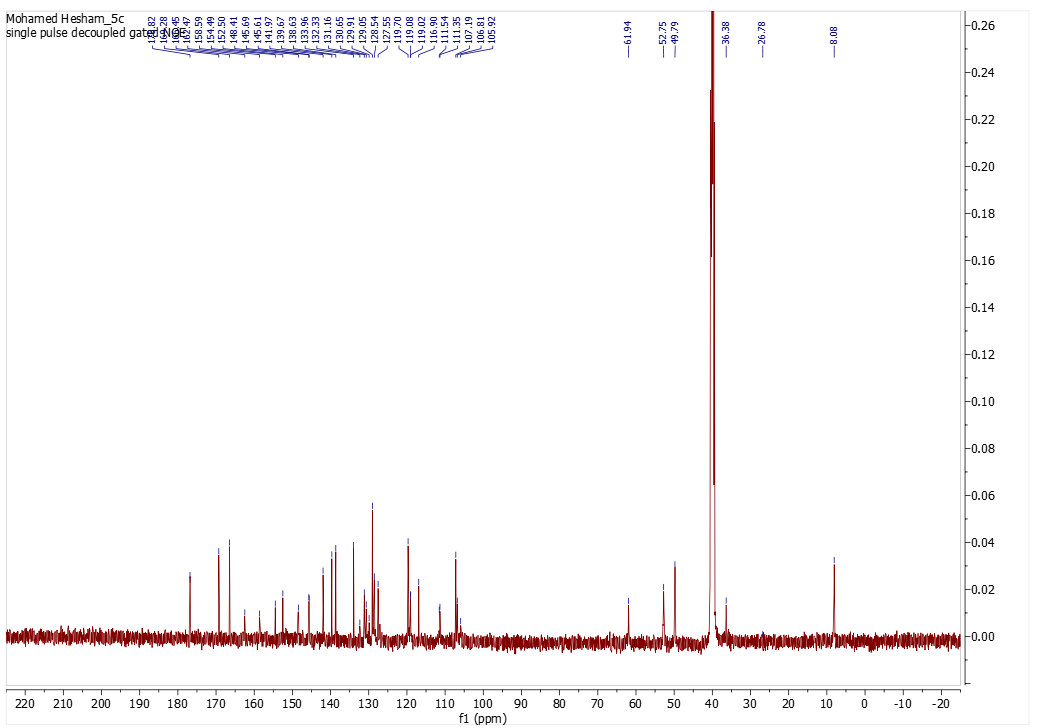


**Figure S14**. ^13^C NMR spectrum of compound **6g** in DMSO-*d*_6_ (125 MHz)


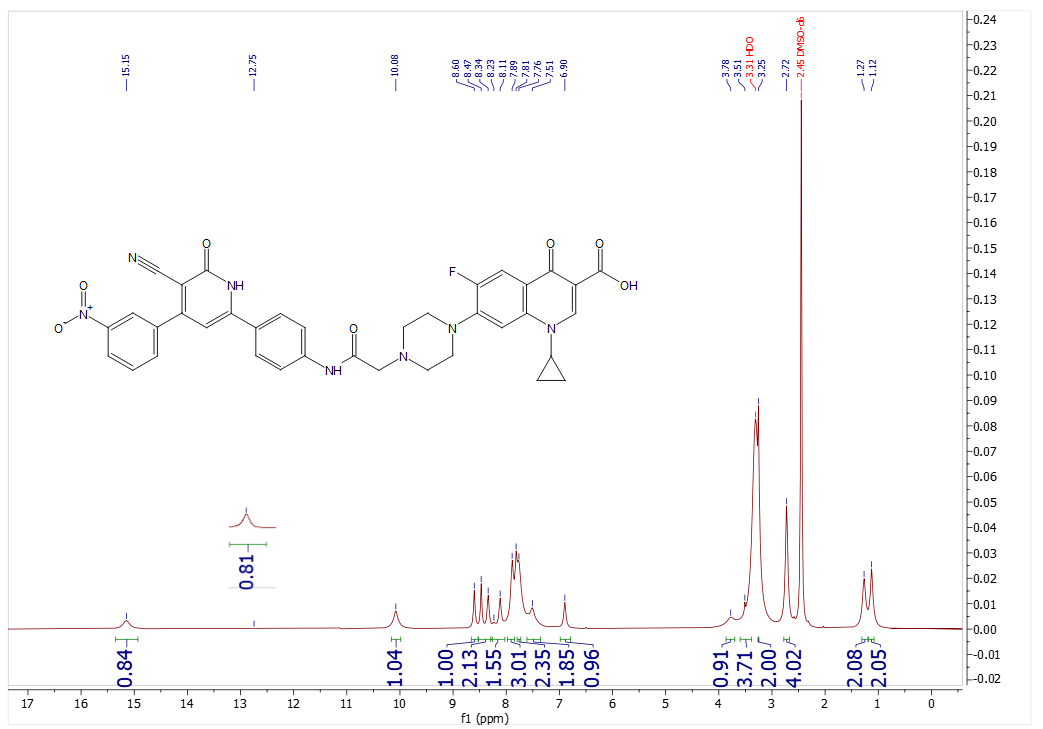


**Figure S15**. ^1^H NMR spectrum of compound **6h** in DMSO-*d*_6_ (400 MHz)


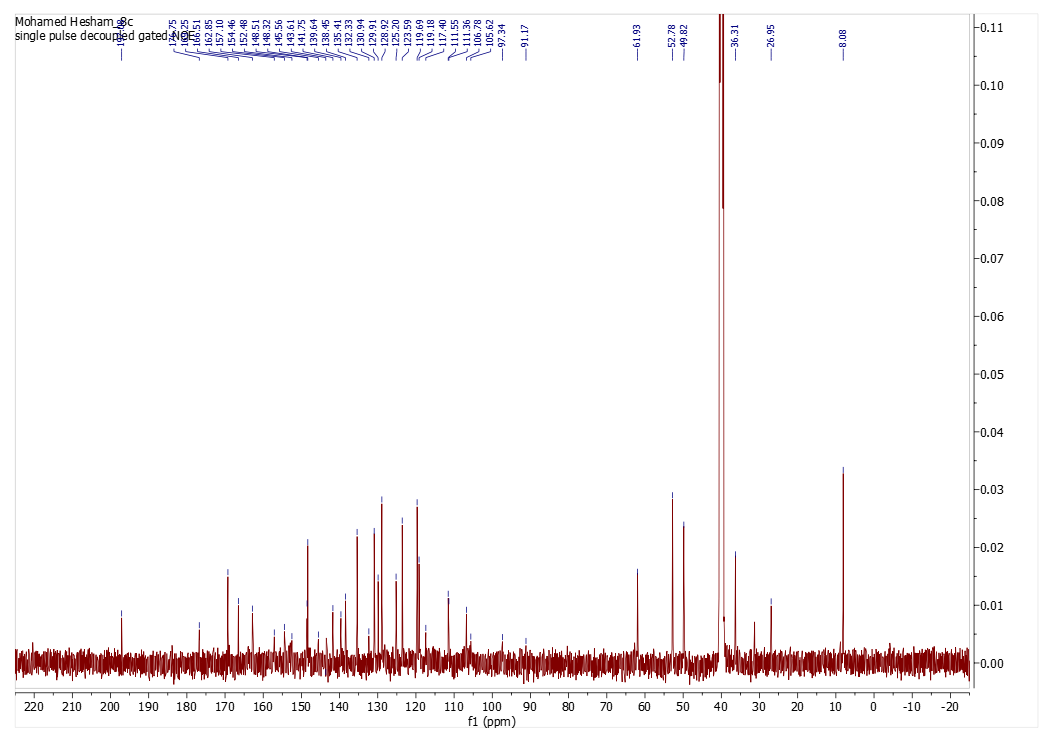


**Figure S16**. ^13^C NMR spectrum of compound **6h** in DMSO-*d*_6_ (125 MHz)


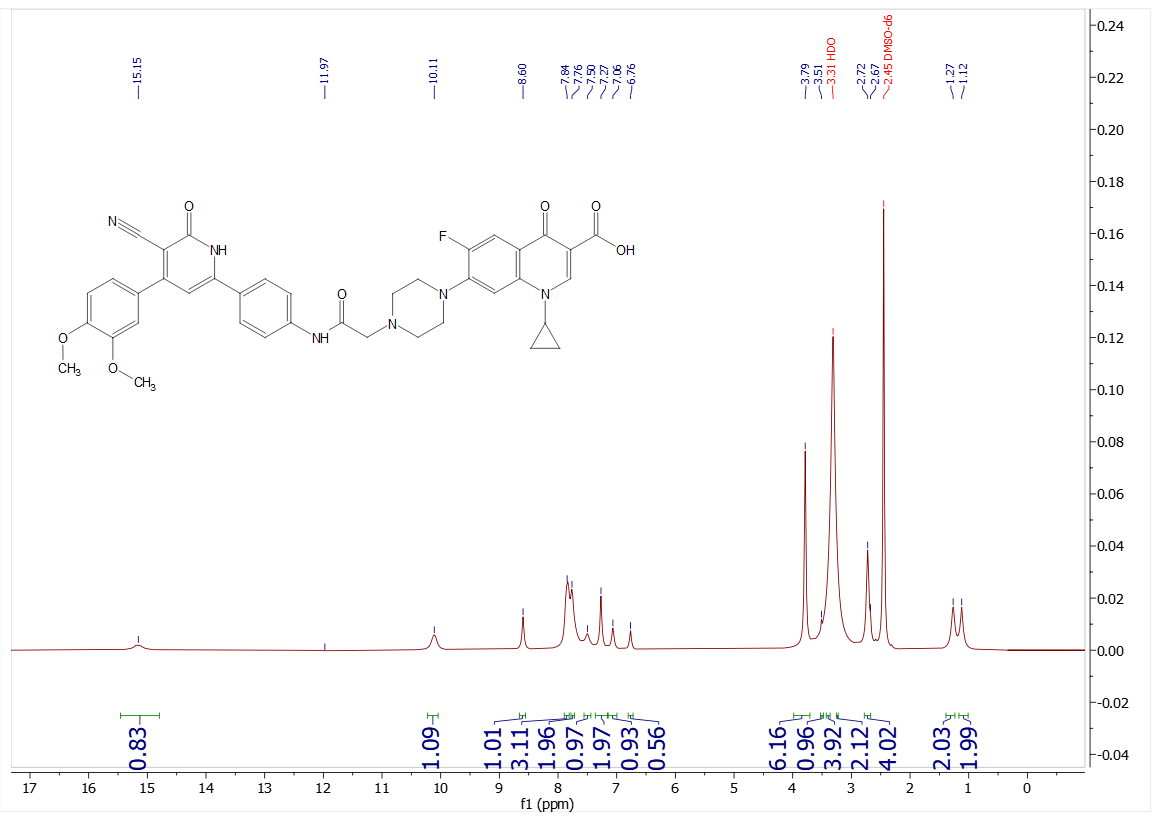


**Figure S17**. ^1^H NMR spectrum of compound **6i** in DMSO-*d*_6_ (400 MHz)


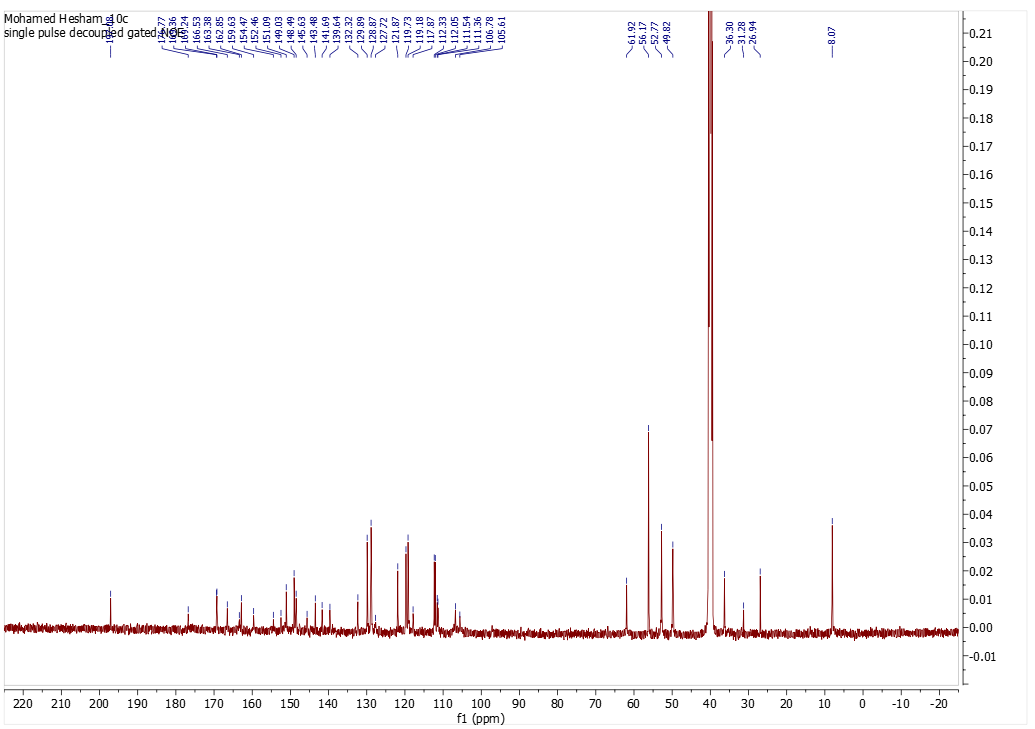


**Figure S18**. ^13^C NMR spectrum of compound **6i** in DMSO-*d*_6_ (125 MHz)


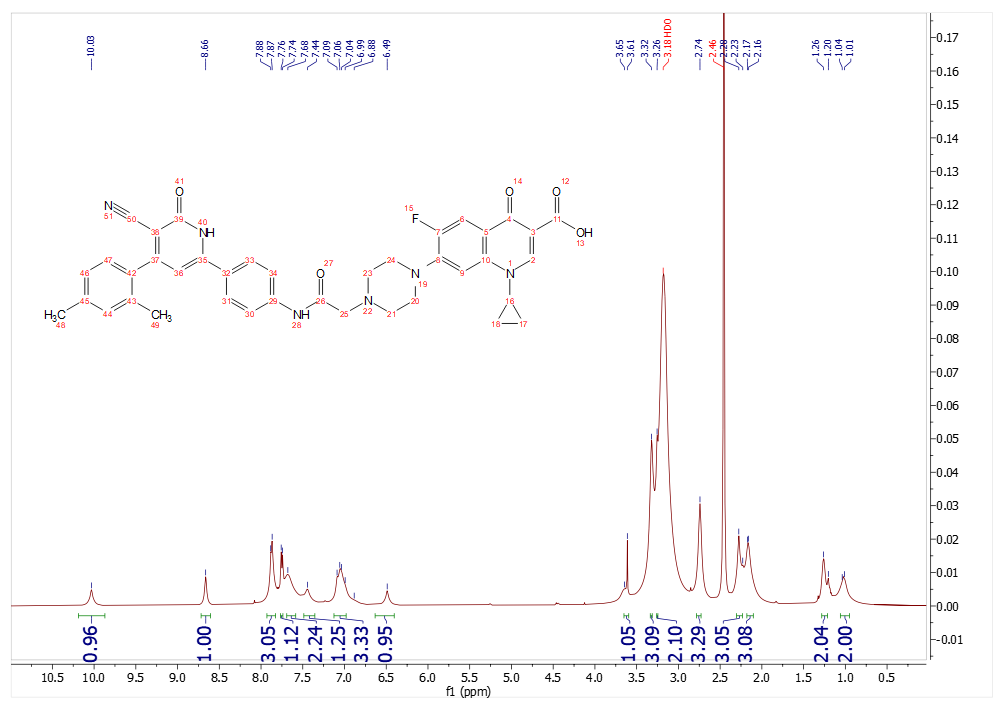


**Figure S19**. ^1^H NMR spectrum of compound **6j** in DMSO-*d*_6_ (400 MHz)


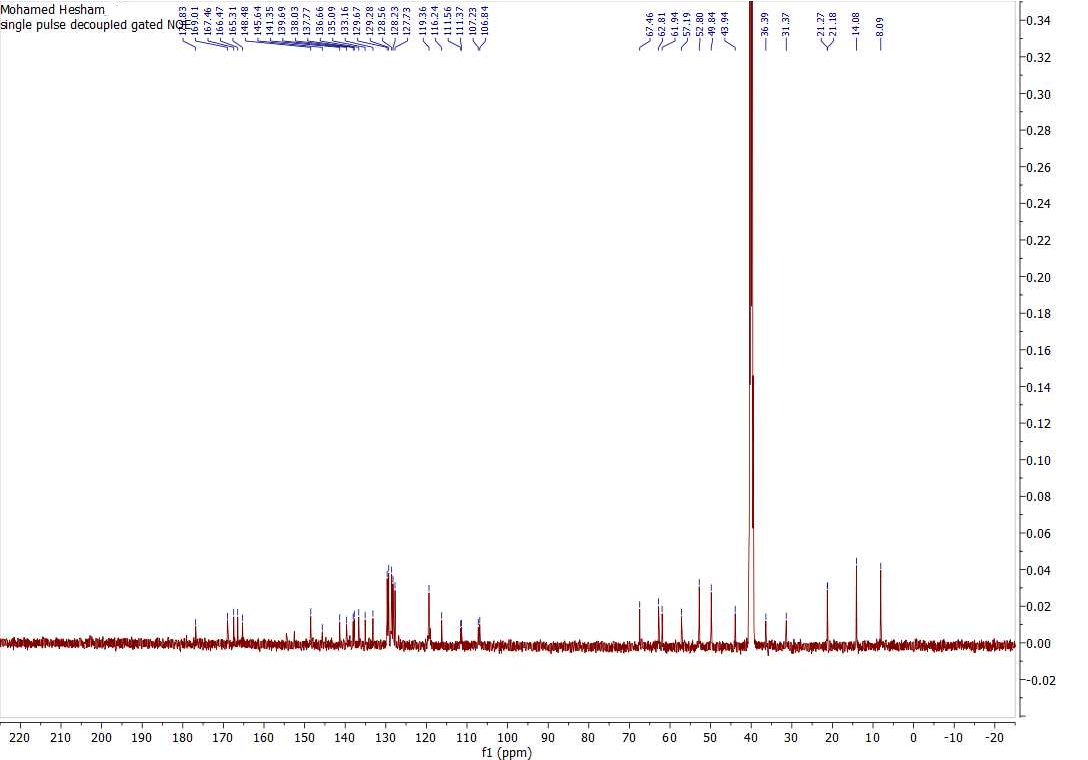


**Figure S20**. ^13^C NMR spectrum of compound **6j** in DMSO-*d*_6_ (125 MHz)


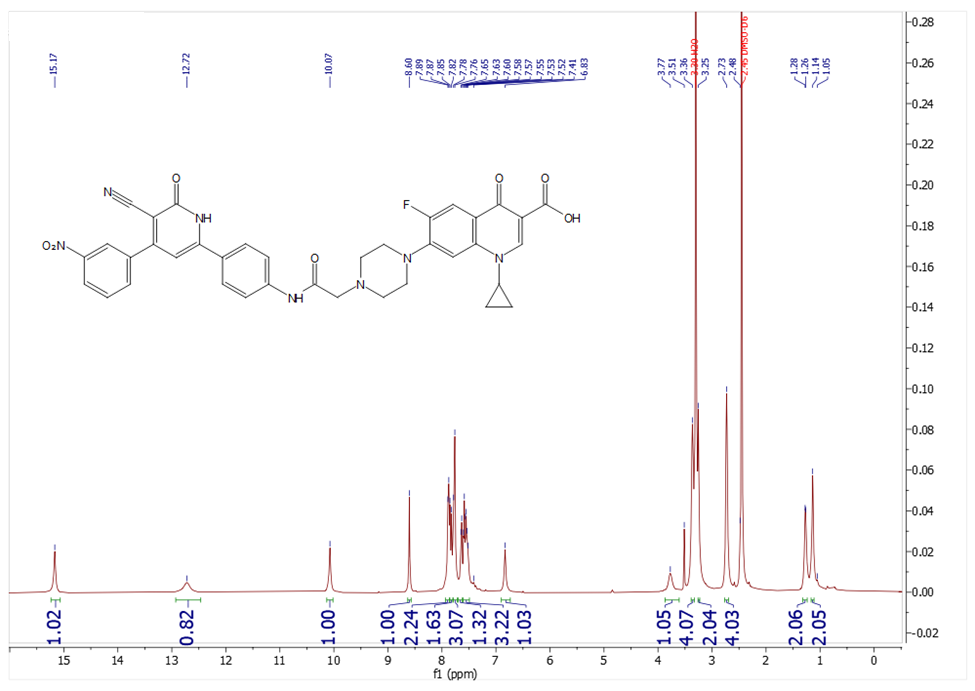


**Figure S21**. ^1^H NMR spectrum of compound **6k** in DMSO-*d*_6_ (400 MHz)


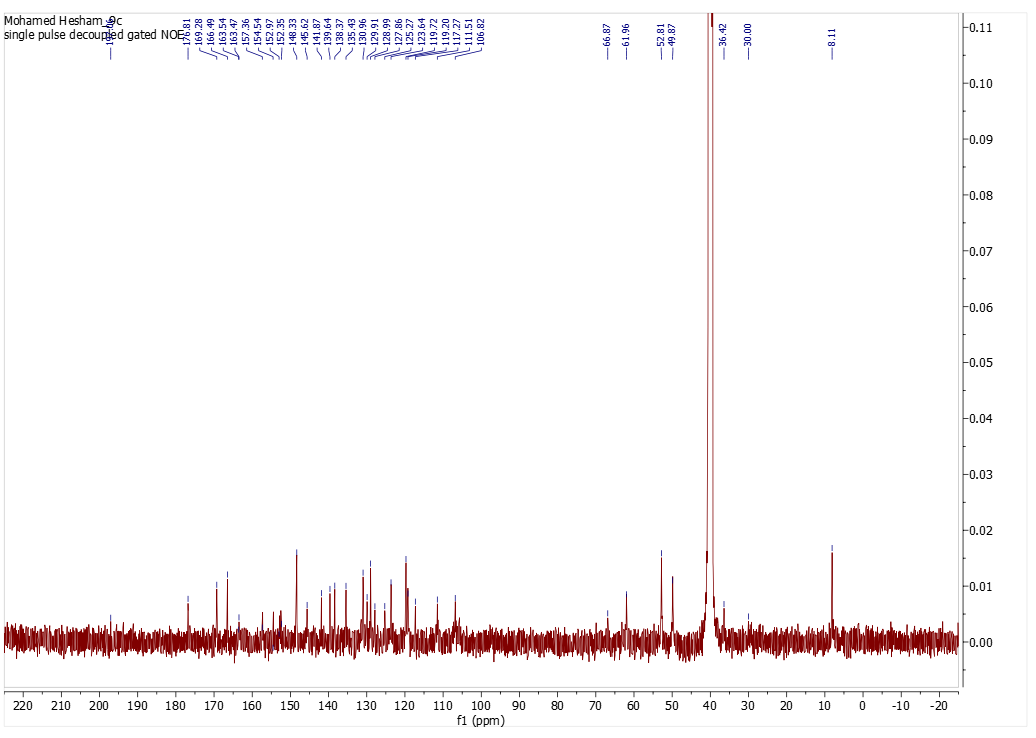


**Figure S22**. ^13^C NMR spectrum of compound **6k** in DMSO-*d*_6_ (125 MHz)


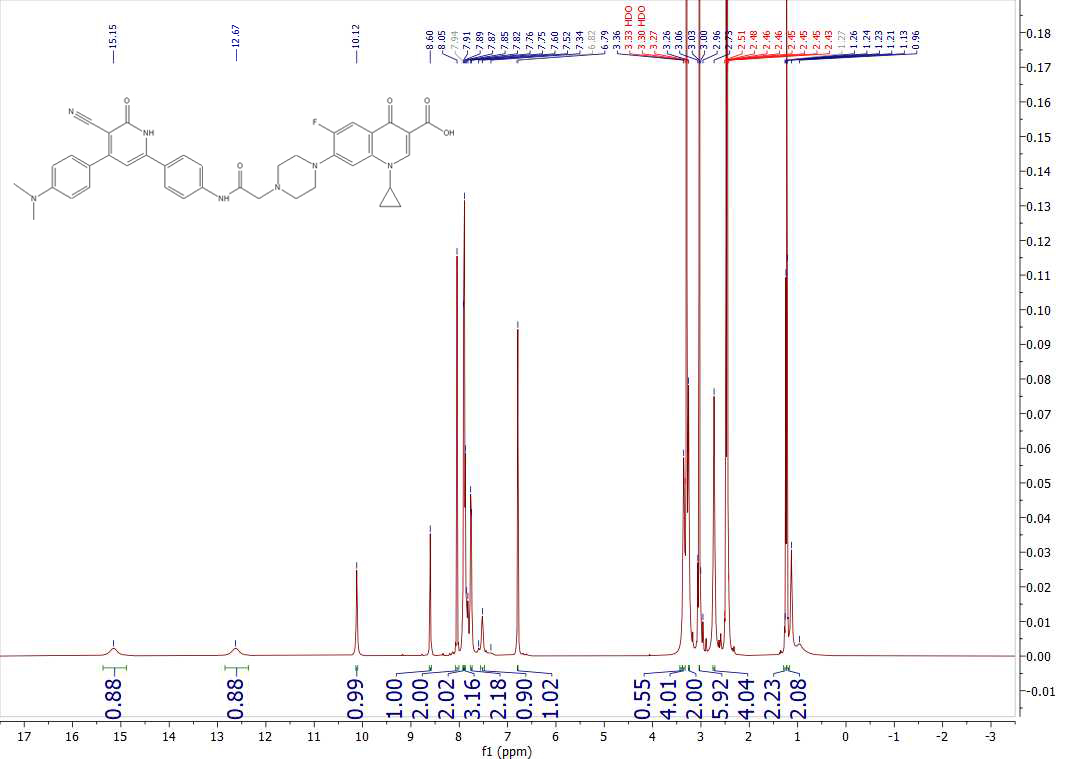


**Figure S23**. ^1^H NMR spectrum of compound **6l** in DMSO-*d*_6_ (400 MHz)

**Figure S24**. ^13^C NMR spectrum of compound **6l** in DMSO-*d*_6_ (125 MHz)

**Figure S25**. Elemental analysis of compounds 6a-6l

**Appendix A**

**4. EXPERIMENTAL**

**4.1. Chemistry**

**General details:**

All chemicals were purchased from Sigma Aldrich, Combi-Blocks, Fisher Scientific and they were used without purification unless mentioned. ^1^H NMR spectra were recorded in DMSOd-6 at 500 MHz on a Bruker AC 500 Ultra shield 10 spectrophotometer. Chemical shifts are expressed in ppm, (δ scale). When peak multiplicities are reported, the following abbreviations are used: s (singlet), d (doublet), m (multiplet), dd (doublet of doublet). Coupling constants are reported in Hertz (Hz). Elemental microanalyses were performed on elemental analyzer model flash 2000 thermo fisher at the regional center for mycology and biotechnology (RCMB), faculty of science, Al-Azhar university, Nasr city, Cairo, Egypt.

**4.2. Antimicrobial activity**

**4.2.1. Organisms and culture conditions**

The cultures used were collected from the Cairo University's Microanalytical Centre, Faculty of Science. An updated Kirby-Bauer disc diffusion method was applied for antimicrobial activities of the tested compounds. Shortly, the 10 ml of fresh medium was grown to 100 μl bacteria / food until a count of 108 cell / ml or 105 cell / ml was achieved. 100 μl microbial suspension has been spread over agar plates that suit the broth in which it was held. Selected colonies of each organism that may play a pathogenic function should be from the primary agar plates. Plates inoculated with Gram positive bacteria as *Staphylococcus aureus* (ATCC 12600)*, Bacillus subtilis* (ATCC 6051); Gram negative bacteria as *Escherichia coli* (ATCC 11775), *Pseudomonas aeuroginosa* (ATCC 10145) they were incubated at 35-37^o^C for 24-48 hours and yeast as *Candida albicans* (ATCC 7102) incubated at 30^o^C for 24-48 hours and, then the diameters of the inhibition zones were measured in millimeters. Standard discs of ciprofloxacin (Antibacterial agent), Fluconazole (Antifungal agent) served as positive controls for antimicrobial activity, but filter discs impregnated with 10 µl of solvent (distilled water, chloroform, DMSO) were used as a negative control. Blank paper disks (Schleicher & Schuell, Spain) with a diameter of 8.0 mm were impregnated 10µ of tested concentration of the stock solutions. When a filter paper disc impregnated with a tested chemical is placed on agar the chemical will diffuse from the disc into the agar. This diffusion will place the chemical in the agar only around the disc. The solubility of the chemical and its molecular size will determine the size of the area of chemical infiltration around the disc. If an organism is placed on the agar, it will not grow in the area around the disc if it is susceptible to the chemical. This area of no growth around the disc is known as a “Zone of inhibition” or" Clear zone". For the disc diffusion, the zone diameters were measured with slipping calipers of the National Committee for Clinical Laboratory Standards, and the results are given in **Table 1**.

**4.2.2. Minimum inhibitory concentration assay**

In 96-well microtiter plates and 50 mL of fresh bacterial culture of a single McFarland unit overnight, a double serial dilution of each compound (100 mL) in sterile standard saline were prepared to every single source well. Ciprofloxacin antibiotic (5 mg / mL-1) and normal saline were included as standard reference in each assay. The plates were incubated at 37 ^0^C overnight. As an indicator of bacterial growth, 40 mL of p-iodonitrotetrazolium violet (INT) was added to each well and incubated at 37 ^0^C for 30 min. MIC values are recorded as the lowest concentration of the extract that completely inhibited bacterial growth that is clear well. The colorless tetrazolidium salt acts as an electron accepter and is reduced to a red colored formazan product by biological activity organisms. Where bacterial growth was inhibited, the solution in the well remained clear after incubation with INT. The observed MIC values are presented in **Table 2**.

**4.2.3. Determination of Inhibitory Activities on *E. coli* DNA Gyrase and Topoisomerase IV**.

All the final compounds were tested for *E. coli* DNA gyrase inhibitory activity in a supercoiling assay. Activities were determined on streptavidin-coated 96-well microtiter plates from Thermo scientific Pierce. First, the plates were rehydrated with buffer (20 mM Tris-HCl with pH 7.6, 0.01% w/v BSA, 0.05% v/v Tween 20, 137 mM NaCl) and the biotinylated oligonucleotide was then immobilized. After washing off the unbound oligonucleotide, the enzyme test was performed. The reaction volume of 30 μL in buffer (35 mM Tris-HCl with pH 7.5, 4 mM MgCl_2_, 24 mM KCl, 2 mM DTT, 1.8 mM spermidine, 1 mM ATP, 6.5 % w/v glycerol, 0.1 mg/mL albumin) contained 1.5 U of DNA gyrase from *E. coli* or *S. aureus*, 0.75 μg of relaxed pNO1 plasmid, and 3 μL solution of the inhibitor in 10% DMSO and 0.008% Tween 20. Reaction solutions were incubated at 37 °C for 30 min. After that, the TF buffer (50 mM NaOAc with pH 5.0, 50 mM NaCl and 50 mM MgCl_2_) was added to terminate the enzymatic reaction. After additional incubation for 30 min at rt, during which biotin-oligonucleotide-plasmid triplex was formed, the unbound plasmid was washed off using TF buffer and SybrGOLD in T10 buffer (10 mM Tris HCl with pH 8.0 and 1 mM EDTA) was added. The fluorescence was measured with a microplate reader (BioTek Synergy H4, excitation: 485 nm, emission: 535 nm). Initial screening was done at 100 or 10 μM concentration of inhibitors. For the most active inhibitors IC_50_ was determined using seven concentrations of tested compounds. GraphPad Prism software was used to calculate the IC_50_ values. The result is given as the average value of three independent measurements. As the internal standard novobiocin (IC_50_ = 0.168 µM for *E. coli* gyrase and IC_50_ = 0.041 µM for *S. aureus* gyrase) was used. Determination of inhibitory activities on *E. coli* and *S. aureus* Topoisomerase IV. IC_50_ values were determined in an assay from In spiralis on streptavidin-coated 96-well microtiter plates from Thermo scientific Pierce. First, the plates were rehydrated with buffer (20 µM Tris-HCl with pH 7.6, 0.01% w/v BSA, 0.05% v/v Tween 20, 137 mM NaCl) and biotinylated oligonucleotide was then immobilized. After washing off the unbound oligonucleotide, the enzyme test was performed. The reaction volume of 30 μL in buffer (40 mM HEPES KOH with pH 7.6, 100 mM potassium glutamate, 10 mM magnesium acetate, 10 mM DTT, 1 mM ATP, 0.05 mg/mL albumin) contained 1.5 U of topoisomerase IV from *E. coli* or *S. aureus*, 0.75 μg of pNO1 supercoiled plasmid, and 3 μL solution of the inhibitor in DMSO (10%) and Tween 20 (0.008%). Reaction mixtures were incubated at 37 °C for 30 min and after that, the TF buffer (50 mM NaOAc with pH 5.0, 50 mM NaCl and 50 mM MgCl_2_) was added to terminate the enzymatic reaction. After additional incubation for 30 min at rt, during which triplex (biotin-oligonucleotide-plasmid) was formed, the unbound plasmid was washed off using TF buffer and Sybr GOLD in T10 buffer (10 mM Tris HCl with pH 8.0 and 1 mM EDTA) was added. The fluorescence was measured with a microplate reader (BioTek Synergy H4, excitation: 485 nm, emission: 535 nm). Initial screening was done at 100 or 10 μM concentration of inhibitors. For the most active inhibitors IC_50_ was determined using seven concentrations of tested compounds. GraphPad Prism software was used to calculate the IC_50_ values. The result is given as the average value of three independent measurements.

**4.2.3. Cell Viability assay**

MTT assay was carried out to study the effect of compounds on mammary epithelial cells (MCF-10A). The medium in which cells were propagated contained Dulbecco's modified Eagle's medium (DMEM)/ Ham's F-12 medium (1:1) supplemented with epidermal growth factor (20 ng/mL), hydrocortisone (500 ng/mL), insulin (10 μg/mL), 2 mM glutamine and 10% fetal calf serum. After every 2-3 days, the cells were passaged using trypsin ethylenediamine tetra acetic acid (EDTA). The cells were seeded at a density of 10^4^ cells mL^-1^ in flat-bottomed culture plates containing 96 wells each. After 24 h, medium was removed from the plates and the compounds in (in 0.1% DMSO) were added (in 200 μL medium to yield a final concentration of 0.1% v/v) to the wells of plates. A single compound was designated with four wells followed by incubation of plates for 96h at 37°C. After incubation, medium was removed completely from the plates followed by addition of MTT (0.4 mg/mL in medium) to each well and subsequent incubation of plates for 3h. MTT (along with the medium) was removed and DMSO (150μL) was added to each well of the culture plates, followed by vortexing and subsequent measurement of absorbance (at 540 nm) using microplate reader. The data are shown as percentage inhibition of proliferation in comparison with controls containing 0.1% DMSO.

ULTRA ADVANCED BIOTECHNOLOGY

RESEARCH

*Bacterial Biofilm*

**Referred from : Prof. Dr Bahaa Gamal**

**Biofilm inhibition ability Raw Data (Absorbance)**

| **Anti Biofilm of Staph aureus** | Replicate1 Ab | Replicate2 Ab | Replicate 3 Ab | Mean |
| --- | --- | --- | --- | --- |
| Blank (Media only) | **0** | **0.001** | **0.002** | 0.001 |
| Media + Organism (Cont.) | **0.987** | **0.947** | **0.956** | 2.675 |
| 1/4 of MIC | **0.341** | **0.35** | **0.348** | 0.372 |
| 1/2 MIC | **0.113** | **0.121** | **0.125** | 0.189 |
| MIC | **0.034** | **0.026** | **0.031** | 0.116 |

| **Anti Biofilm of Staph aureus** | **Biofilm Inhibition %** | | | Mean **Inhibition %** | SD (±) |
| --- | --- | --- | --- | --- | --- |
|  | Replicate 1 Ab | Replicate 2 Ab | Replicate 3 Ab |  |  |
| 1/4 of MIC | **63.66** | **62.72** | **62.93** | **63.10** | **0.50** |
| 1/2 MIC | **87.36** | **86.53** | **86.11** | **86.66** | **0.60** |
| MIC | **95.57** | **96.40** | **95.88** | **95.95** | **0.40** |

**Note:**

**Blank represented absorbance of media only**

**Control represented absorbance of test organism without any treatment.**

**
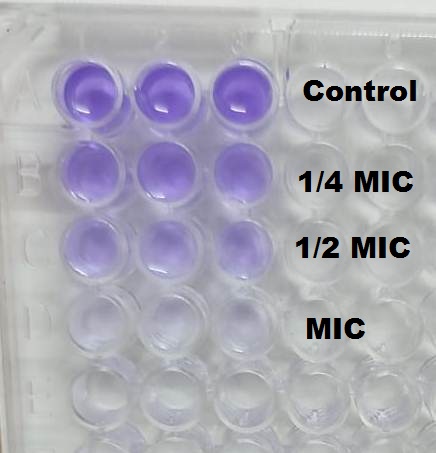
**

**Methods:**

**Microtiter plate assay for biofilm quantification**

The effect of sample on biofilm formation was evaluated in 96-well polystyrene flatbottom plates. Briefly, 300 𝜇L of inoculated fresh trypticase soy yeast broth (TSY) (final concentration 106 CFU/mL) was aliquoted into each well of microplate and cultured in presence of sublethal concentrations (MIC, 1/2 MIC; 1/4 MIC) previously determined (MIC value 0.025 ug/ml). Wells containing medium and those without extracts and only with methanol were used as controls. Plates were incubated at 37∘C for 48h. After incubation, supernatant was removed and each well was washed thoroughly with sterile distilled water to remove free-floating cells; thereafter plates were air-dried for 30min, and the biofilm formed was stained during 15 min at room temperature with 0.1% aqueous solution of crystal violet. Following incubation, the excess of stain was removed by washing the plate three times with sterile distilled water. Finally, the dye bound to the cells was solubilized by adding 250 𝜇L of 95% ethanol to each well and after 15min of incubation, absorbance was measured using microplate reader at a wavelength of 570 nm.

**Biofilm inhibition ability of sample=(1- (absorb. sample - absorb. Blank))/ (absorb. control - absorb. Blank))*100**

**Researcher** : Dr.Mohamed Hisham

email: [Mohammedhisham90@yahoo.com](mailto:Mohammedhisham90@yahoo.com)

**mob.** 01005626269

Assay : DNA gyrase supercoiling

Samples : 12 samples

Ref. : *---*

Date : 29-10-2025

Reader : ---

Cell line : ---

Kit used : E.Coli DNA Gyrase kit **Inspirals**

Solvent : DMSO

Assay samples : ---

**Lab Report**

| **ser** | **Compound** | | | **DNA gyrase**  **Supercoiling** | SD |
| --- | --- | --- | --- | --- | --- |
|  | **code** | **MW** | **conc**  **ug** | **IC50**  **uM** |  |
| 1 | **1C** | 658.69 | --- | **14.09**±0.41 |  |
| 2 | **2C** | 672.72 | --- | **6.156**±0.18 |  |
| 3 | **3C** | 688.72 | --- | **57.73**±1.7 |  |
| 4 | **4C** | 693.13 | --- | **1.868**±0.06 |  |
| 5 | **5C** | 693.13 | --- | **1.751**±0.05 |  |
| 6 | **6C** | 737.59 | --- | **11.39**±0.34 |  |
| 7 | **7C** | 676.68 | --- | **2.687**±0.08 |  |
| 8 | **8C** | 703.69 | --- | **9.703**±0.29 |  |
| 9 | **9C** | 703.69 | --- | **160.7**±4.73 |  |
| 10 | **10C** | 718.74 | --- | **5.725**±0.17 |  |
| 11 | **11C** | 686.74 | --- | **29.67**±0.87 |  |
| 12 | **12C** | 701.76 | --- | **3.894**±0.11 |  |
| * | Novobiocin | 612.62 | --- | **0.778**±0.02 |  |
| ** | ciprofloxacin | 331.34 | --- | **2.128**±0.06 |  |


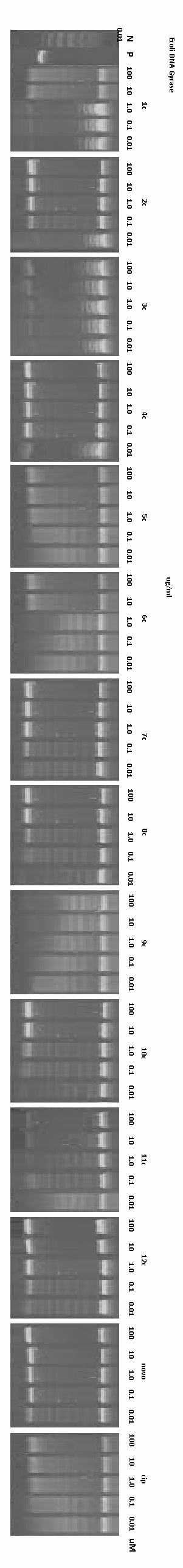
Detailed results


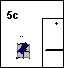


| **DNA Gyrase** | | |  |  |
| --- | --- | --- | --- | --- |
| code | IC50 | conc | log | %inh |
| 1c |  | 100 | 2 | 77 |
| 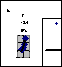 |  | 10 | 1 | 42 |
|  |  | 1 | 0 | 15 |
|  |  | 0.1 | -1 | 7.1 |
|  |  | 0.01 | -2 | 2.3 |
| EC |  |  |  | 0 |
|  |  |  |  |  |
| code | IC50 | conc | log | %inh |
| 2c |  | 100 | 2 | 81 |
| 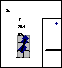 |  | 10 | 1 | 57 |
|  |  | 1 | 0 | 21 |
|  |  | 0.1 | -1 | 7.2 |
|  |  | 0.01 | -2 | 3.4 |
| EC |  |  |  | 0 |
|  |  |  |  |  |
| code | IC50 | conc | log | %inh |
| 3c |  | 100 | 2 | 72 |
| 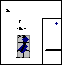 |  | 10 | 1 | 23 |
|  |  | 1 | 0 | 7.7 |
|  |  | 0.1 | -1 | 4.5 |
|  |  | 0.01 | -2 | 1.6 |
| EC |  |  |  | 0 |
|  |  |  |  |  |
| code | IC50 | conc | log | %inh |
| 4c |  | 100 | 2 | 87 |
| 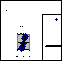 |  | 10 | 1 | 71 |
|  |  | 1 | 0 | 37 |
|  |  | 0.1 | -1 | 19 |
|  |  | 0.01 | -2 | 6.7 |
| EC |  |  |  | 0 |
|  |  |  |  |  |
| code | IC50 | conc | log | %inh |
| 5c |  | 100 | 2 | 88 |
|  |  | 10 | 1 | 72 |
|  |  | 1 | 0 | 40 |
|  |  | 0.1 | -1 | 15 |
|  |  | 0.01 | -2 | 7.4 |
| EC |  |  |  | 0 |
|  |  |  |  |  |
| code | IC50 | conc | log | %inh |
| 6c |  | 100 | 2 | 78 |
| 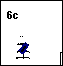 |  | 10 | 1 | 49 |
|  |  | 1 | 0 | 12 |
|  |  | 0.1 | -1 | 5.3 |
|  |  | 0.01 | -2 | 3.1 |
| EC |  |  |  | 0 |
|  |  |  |  |  |
| code | IC50 | conc | log | %inh |
| 7c |  | 100 | 2 | 88 |
| 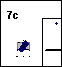 |  | 50 | 1.7 | 73 |
|  |  | 1 | 0 | 38 |
|  |  | 0.1 | -1 | 15 |
|  |  | 0.01 | -2 | 7 |
| EC |  |  |  | 0 |
|  |  |  |  |  |
| code | IC50 | conc | log | %inh |
| 8c |  | 100 | 2 | 81 |
|  |  | 50 | 1.7 | 57 |
| 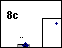 |  | 1 | 0 | 20 |
|  |  | 0.1 | -1 | 8.1 |
|  |  | 0.01 | -2 | 4.9 |
| EC |  |  |  | 0 |
|  |  |  |  |  |
| code | IC50 | conc | log | %inh |
| 9c |  | 100 | 2 | 71 |
| 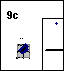 |  | 50 | 1.7 | 24 |
|  |  | 1 | 0 | 7.9 |
|  |  | 0.1 | -1 | 3.8 |
|  |  | 0.01 | -2 | 1.3 |
| EC |  |  |  | 0 |
|  |  |  |  |  |
| code | IC50 | conc | log | %inh |
| 10c |  | 100 | 2 | 85 |
|  |  | 50 | 1.7 | 66 |
| 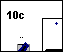 |  | 1 | 0 | 24 |
|  |  | 0.1 | -1 | 8 |
|  |  | 0.01 | -2 | 6 |
| EC |  |  |  | 0 |
|  |  |  |  |  |
| code | IC50 | conc | log | %inh |
| 11c |  | 100 | 2 | 78 |
|  |  | 50 | 1.7 | 38 |
| 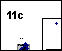 |  | 1 | 0 | 18 |
|  |  | 0.1 | -1 | 8.3 |
|  |  | 0.01 | -2 | 3.9 |
| EC |  |  |  | 0 |
|  |  |  |  |  |
| code | IC50 | conc | log | %inh |
| 12c |  | 100 | 2 | 85 |
| 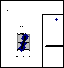 |  | 10 | 1 | 63 |
|  |  | 1 | 0 | 25 |
|  |  | 0.1 | -1 | 8.2 |
|  |  | 0.01 | -2 | 5.4 |
| EC |  |  |  | 0 |
|  |  |  |  |  |
| code | IC50 | conc | log | %inh |
| **Novo** |  | 100 | 2 | 89 |
| 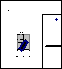 |  | 10 | 1 | 80 |
|  |  | 1 | 0 | 58 |
|  |  | 0.1 | -1 | 27 |
|  |  | 0.01 | -2 | 7.6 |
| EC |  |  |  | 0 |
|  |  |  |  |  |
| code | IC50 | conc | log | %inh |
| 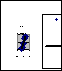**CIP** |  | 100 | 2 | 85 |
|  |  | 10 | 1 | 70 |
|  |  | 1 | 0 | 39 |
|  |  | 0.1 | -1 | 15 |
|  |  | 0.01 | -2 | 6.1 |
| EC |  |  |  | 0 |

**Researcher** : Dr.Mohamed Hisham

email: [Mohammedhisham90@yahoo.com](mailto:Mohammedhisham90@yahoo.com)

**mob.** 01005626269

Assay : DNA TOPO IV

Samples : 12 samples

Ref. : *---*

Date : 29-10-2025

Reader : ---

Cell line : ---

Kit used : E.Coli DNA TOPO IV kit **Inspirals**

Solvent : DMSO

Assay samples : ---

**Lab Report**

| **ser** | **Compound** | | | **DNA –TOPO IV**  **Relaxation** | SD |
| --- | --- | --- | --- | --- | --- |
|  | **code** | **MW** | **conc**  **ug** | **IC50**  **uM** |  |
| 1 | **1C** | 658.69 | --- | **22.06**±0.89 |  |
| 2 | **2C** | 672.72 | --- | **35.34**±1.42 |  |
| 3 | **3C** | 688.72 | --- | **15.7**±0.63 |  |
| 4 | **4C** | 693.13 | --- | **11.92**±0.48 |  |
| 5 | **5C** | 693.13 | --- | **3.47**±0.14 |  |
| 6 | **6C** | 737.59 | --- | **27.31**±1.1 |  |
| 7 | **7C** | 676.68 | --- | **5.562**±0.22 |  |
| 8 | **8C** | 703.69 | --- | **40.99**±1.65 |  |
| 9 | **9C** | 703.69 | --- | **31.23**±1.26 |  |
| 10 | **10C** | 718.74 | --- | **8.167**±0.33 |  |
| 11 | **11C** | 686.74 | --- | **83.44**±3.36 |  |
| 12 | **12C** | 701.76 | --- | **4.507**±0.18 |  |
| * | Novobiocin | 612.62 | --- | **10.62**±**0.46** |  |
| ** | ciprofloxacin | 331.34 | --- | **25.22**±**1.27** |  |

Detailed results


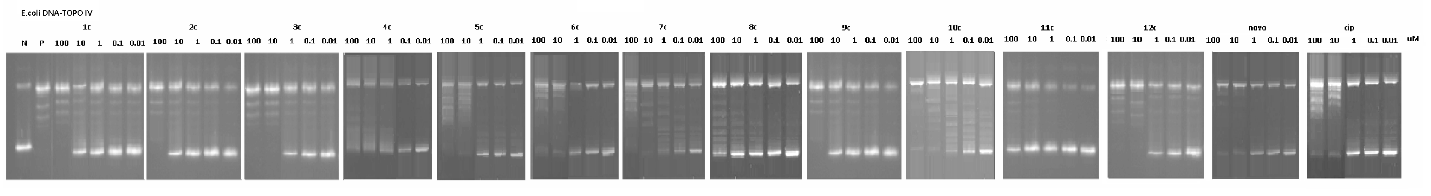


| **DNA-TOPO IV** | | |  |  |
| --- | --- | --- | --- | --- |
| code | IC50 | conc | log | %inh |
| 1c |  | 100 | 2 | 75 |
| 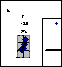 |  | 10 | 1 | 39 |
|  |  | 1 | 0 | 8.9 |
|  |  | 0.1 | -1 | 4.6 |
|  |  | 0.01 | -2 | 1.8 |
| EC |  |  |  | 0 |
|  |  |  |  |  |
| code | IC50 | conc | log | %inh |
| 2c |  | 100 | 2 | 73 |
| 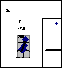 |  | 10 | 1 | 31 |
|  |  | 1 | 0 | 7.2 |
|  |  | 0.1 | -1 | 3.6 |
|  |  | 0.01 | -2 | 1.4 |
| EC |  |  |  | 0 |
|  |  |  |  |  |
| code | IC50 | conc | log | %inh |
| 3c |  | 100 | 2 | 83 |
| 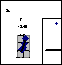 |  | 10 | 1 | 37 |
|  |  | 1 | 0 | 8.9 |
|  |  | 0.1 | -1 | 3.8 |
|  |  | 0.01 | -2 | 2.2 |
| EC |  |  |  | 0 |
|  |  |  |  |  |
| code | IC50 | conc | log | %inh |
| 4c |  | 100 | 2 | 79 |
| 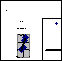 |  | 10 | 1 | 42 |
|  |  | 1 | 0 | 18 |
|  |  | 0.1 | -1 | 7.3 |
|  |  | 0.01 | -2 | 3.9 |
| EC |  |  |  | 0 |
|  |  |  |  |  |
| code | IC50 | conc | log | %inh |
| 5c |  | 100 | 2 | 89 |
| 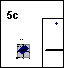 |  | 10 | 1 | 71 |
|  |  | 1 | 0 | 33 |
|  |  | 0.1 | -1 | 8.9 |
|  |  | 0.01 | -2 | 5.5 |
| EC |  |  |  | 0 |
|  |  |  |  |  |
| code | IC50 | conc | log | %inh |
| 6c |  | 100 | 2 | 71 |
| 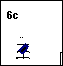 |  | 10 | 1 | 41 |
|  |  | 1 | 0 | 7.6 |
|  |  | 0.1 | -1 | 3.8 |
|  |  | 0.01 | -2 | 1.4 |
| EC |  |  |  | 0 |
|  |  |  |  |  |
| code | IC50 | conc | log | %inh |
| 7c |  | 100 | 2 | 82 |
| 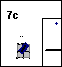 |  | 50 | 1.7 | 60 |
|  |  | 1 | 0 | 18 |
|  |  | 0.1 | -1 | 7.7 |
|  |  | 0.01 | -2 | 3.4 |
| EC |  |  |  | 0 |
|  |  |  |  |  |
| code | IC50 | conc | log | %inh |
| 8c |  | 100 | 2 | 73 |
|  |  | 50 | 1.7 | 42 |
| 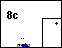 |  | 1 | 0 | 8.6 |
|  |  | 0.1 | -1 | 5.3 |
|  |  | 0.01 | -2 | 1.3 |
| EC |  |  |  | 0 |
|  |  |  |  |  |
| code | IC50 | conc | log | %inh |
| 9c |  | 100 | 2 | 77 |
| 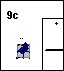 |  | 50 | 1.7 | 39 |
|  |  | 1 | 0 | 12 |
|  |  | 0.1 | -1 | 6.4 |
|  |  | 0.01 | -2 | 2.1 |
| EC |  |  |  | 0 |
|  |  |  |  |  |
| code | IC50 | conc | log | %inh |
| 10c |  | 100 | 2 | 82 |
|  |  | 50 | 1.7 | 54 |
| 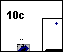 |  | 1 | 0 | 11 |
|  |  | 0.1 | -1 | 6.9 |
|  |  | 0.01 | -2 | 2.9 |
| EC |  |  |  | 0 |
|  |  |  |  |  |
| code | IC50 | conc | log | %inh |
| 11c |  | 100 | 2 | 76 |
|  |  | 50 | 1.7 | 27 |
| 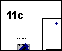 |  | 1 | 0 | 7.3 |
|  |  | 0.1 | -1 | 3.9 |
|  |  | 0.01 | -2 | 1.2 |
| EC |  |  |  | 0 |
|  |  |  |  |  |
| code | IC50 | conc | log | %inh |
| 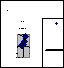12c |  | 100 | 2 | 87 |
|  |  | 10 | 1 | 67 |
|  |  | 1 | 0 | 27 |
|  |  | 0.1 | -1 | 10 |
|  |  | 0.01 | -2 | 6.5 |
| EC |  |  |  | 0 |
|  |  |  |  |  |
| code | IC50 | conc | log | %inh |
| **Novo** |  | 100 | 2 | 76 |
| 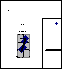 |  | 10 | 1 | 52 |
|  |  | 1 | 0 | 13 |
|  |  | 0.1 | -1 | 5.6 |
|  |  | 0.01 | -2 | 1.1 |
| EC |  |  |  | 0 |
|  |  |  |  |  |
| code | IC50 | conc | log | %inh |
| **CIP** |  | 100 | 2 | 71 |
| 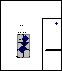 |  | 10 | 1 | 41 |
|  |  | 1 | 0 | 7.9 |
|  |  | 0.1 | -1 | 4.4 |
|  |  | 0.01 | -2 | 0.6 |
| EC |  |  |  | 0 |
